# Anisotropic core–shell ceramic nanofibrous membrane with improved optical reflectivity and thermal insulation for high-energy laser protection

**DOI:** 10.1038/s41467-026-73159-0

**Published:** 2026-07-23

**Authors:** Huihuang Ma, Yikun Liu, Jianfei Gao, Xia Yang, Luo Luo, Yunfeng Tang, Xiaodong Zhou, Liangshun Zhang

**Affiliations:** 1 Key Laboratory of Specially Functional Polymeric Materials and Related Technology (Ministry of Education), School of Chemical Engineering, East China University of Science and Technology, Shanghai, China; 2 Shanghai Key Laboratory of Multiphase Materials Chemical Engineering, School of Chemical Engineering, East China University of Science and Technology, Shanghai, China; 3 School of Chemisty and Chemical Engineering, Henan University of Technology, Zhengzhou, China; 4 Institute for Engineering and Technology (Shanghai), Xinxing Cathay International Group, Shanghai, China; 5 Shanghai Engineering Research Center of Hierarchical Nanomaterials, East China University of Science and Technology, Shanghai, China; 6 Shanghai Key Laboratory of Advanced Polymeric Materials, School of Materials Science and Engineering, East China University of Science and Technology, Shanghai, China

**Keywords:** Nanowires, Structural properties

## Abstract

The development of multifunctionally coupled materials that can adapt to complex thermophotonic environments is increasingly critical for next-generation aerospace, defense, and high-energy laser applications. Ceramic nanofibers, though inherently robust, often struggle to reconcile optical reflectivity with directional thermal management under extreme conditions. Here, we present a core–shell ceramic nanofibrous membrane, comprising a mesoporous silica shell and a crystalline boron nitride core, which exhibits dynamic thermal-optical functionality tailored to high-flux laser exposure. By virtue of dual-channel electrospinning and calcination, the structure achieves high reflectivity (≈ 100% at 1064 nm) while enabling anisotropic thermal conduction—rapidly dissipating heat in the membrane plane while insulating through its thickness. This functional decoupling at the single-fiber level empowers the membrane to maintain mechanical and structural integrity above 1500 °C, outperforming conventional ceramic or polymer systems in both laser shielding and thermal resilience. Moreover, the membrane demonstrates a compressive elasticity of 95% strain, a tensile strength of ≈ 20 MPa, and structural stability under a real-time laser impact. By introducing a design strategy that couples optical scattering with spatially controlled heat flow, this work provides an effective pathway for designing adaptive ceramic nanofiber systems engineered to thrive in complex, coupled-mechanism environments.

## Abstract (web summary)

Ceramic nanofibers often struggle to reconcile optical reflectivity with thermal management. Here, the authors design a core-shell ceramic nanofiber membrane that achieves high laser shielding and directional heat dissipation

## Introduction

Ceramic nanofibers (CNFs) are characterized by distinct nanoscale architectures that endow them with robust thermal stability, high specific surface areas, and high mechanical strength, collectively underpinning their broad potential in aerospace thermal insulation, protective barriers, and advanced thermal management systems^1–10^. Recent advances have yielded ceramic nanofiber materials with enhanced functionalities. For example, super-white boron nitride (BN) aerogels exhibit high broadband optical reflectivity (>98%) and low thermal conductivity, providing advantages in thermal insulation and high-energy laser protection^11^. Similarly, zirconia (ZrO_2_) nanofiber aerogels with hypocrytalline structures show near-zero thermal expansion and low thermal conductivity (≈ 0.01 W m⁻¹ K⁻¹), highlighting their promise for managing extreme thermal stresses^12^. Nevertheless, engineering ceramic nanofibers to withstand the complex extreme conditions encountered during high-energy laser irradiation remains a challenge, owing primarily to their inherent limitations in thermal management and structural resilience under severe thermal loads^13–15^. Consequently, realizing multi-energy coupling scenarios (e.g., photoelectric, thermoelectric, and photothermal coupling) with single-component ceramic nanofibers is difficult. Therefore, functionalization of ceramic nanofibers emerges as a crucial strategy to overcome these limitations and enable complex multi-energy-coupling functionalities.

High-energy laser irradiation, known for its photothermal coupling effects, imposes stringent performance requirements on protective materials. The concentrated energy deposition rapidly elevates surface temperatures, inducing structural damage characterized by thermal shock, phase transformations, material ablation, and mechanical fracture. Traditional protective materials—including dense ceramics^16–21^ and polymer-based composites^22–25^—frequently fail under these conditions due to inadequate reflectivity, limited thermal dissipation capabilities, and insufficient mechanical stability, underscoring the necessity for material systems optimized for extreme laser protection.

Recent advancements in anisotropic thermal materials offer promising solutions to these challenges. Materials such as oriented polymer composites, aligned graphene architectures, and boron nitride-based scaffolds are being developed for their enhanced thermal management^26^. These materials leverage their anisotropic properties to direct heat flow effectively, enhancing thermal dissipation. Oriented polymer composites spread heat in-plane, while aligned graphene structures use their high conductivity for rapid heat diffusion^27^. Boron nitride-based scaffolds provide a balanced solution, combining high thermal conductivity with effective insulation properties for both heat dissipation and thermal shielding^28^. These innovations highlight the potential for optimizing materials for extreme laser protection^29,30^.

Among promising candidates, SiO_2_ nanofibers have garnered substantial interest for high-energy laser protection applications, attributed largely to their inherently high optical reflectivity resulting from enhanced Rayleigh and Raman scattering effects intrinsic to their nanoscale fibrous networks^31–35^. However, pure SiO_2_ nanofibers inherently exhibit limited thermal insulation capabilities, leading to significant thermal accumulation and rapid temperature rise during continuous laser exposure^36,37^. Thus, integrating effective thermal management functionalities within these fibers is crucial to fully exploit their reflective advantages while preventing structural degradation under prolonged high-energy laser irradiation.

While functional ceramics can enhance the thermal management of SiO_2_ nanofibers, their high laser energy absorption often induces detrimental thermal expansion and phase transitions, leading to structural failure. Illustrating a direction for improvement, Pan et al. recently developed silica-based core-shell nanofibers that achieve dual-functional thermal management by combining low thermal conductivity with high reflectivity and emissivity^38^. Despite such advances, maintaining structural integrity and high optical reflectivity under high-energy laser irradiation remains a challenge for practical laser shielding applications. The core bottleneck, therefore, lies in integrating thermally conductive phases into SiO_2_ nanofibers while ensuring absolute chemical inertness and minimal surface energy absorption. This necessitates rational material design and fabrication strategies to achieve robust integration of these functional phases without compromising the structural or optical properties of the nanofiber matrix.

To address these bottlenecks, we have developed a core–shell nanofiber membrane that integrates high-thermal-conductivity BN within SiO_2_ fibers via precision electrospinning and controlled calcination. This core–shell architecture endows the otherwise highly reflective SiO_2_ membrane with anisotropic thermal conductivity, preserving its structural integrity and keeping an underlying substrate below critical temperature thresholds even under sustained high-intensity laser irradiation. Comprehensive optical and thermal measurements have confirmed that this design achieves an optimal balance between reflectivity and directed thermal dissipation, resulting in effective overall laser shielding performance. Furthermore, mechanical tests have shown that the membrane maintains robust interfacial bonding and structural stability under extreme thermal stress. Collectively, these results provide valuable insights for the design of next-generation high energy laser (HEL) protective materials and mark a step toward their practical deployment. This study addresses these scientific and technological challenges by proposing and validating strategies for integrating inert SiO_2_ nanofibers with thermally conductive functional ceramics, providing insights into high-performance thermal protection materials suitable for complex photothermal environments.

## Results and discussion

### Design rationale

**Fig. 1:**
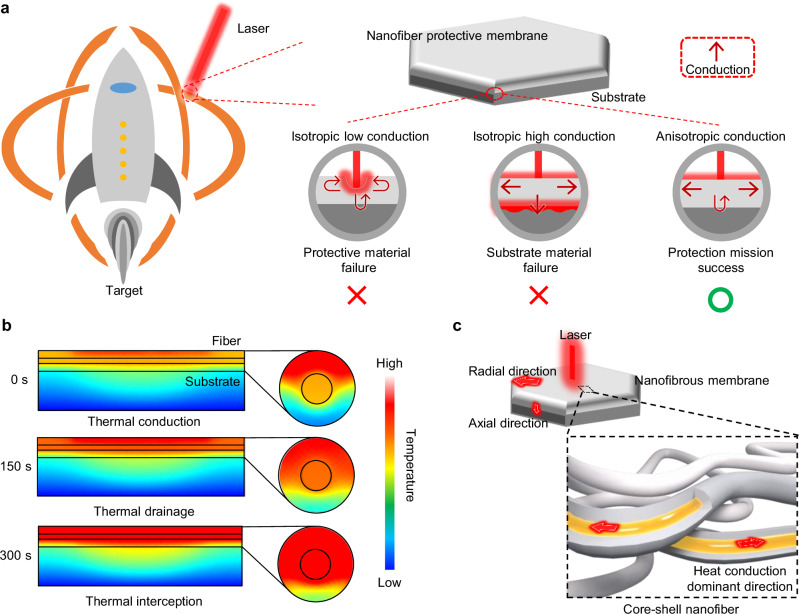
Design concept of core–shell structured nanofibers. **a** Material design considerations derived from the ideal objective of effective laser protection. Light and dark gray indicate the protective and substrate materials, respectively. **b** Simulated thermal conduction behavior in core–shell structured nanofibers. **c** Schematic illustration of the thermal transfer principle in core–shell structured nanofibers, where the yellow region indicates the core material.

In extreme high-energy laser irradiation scenarios (such as those encountered in aircraft protection), it is critical that both the protective layer and the underlying substrate remain intact and free of thermal damage during exposure (Fig. 1a). Conventional protective materials present a dilemma: those with low thermal conductivity (e.g., thermal-resistant resins or aerogels) are prone to localized thermal buildup, whereas those with high thermal conductivity (e.g., ceramics or metals) readily transmit thermal to the substrate. In both cases, the protective function is undermined. An optimal solution is to engineer materials with anisotropic thermal conductivity. By biasing thermal flow predominantly in the radial direction (Throughout this work, radial and axial refer to directions within the macroscopic membrane plane and thickness, respectively), most incident thermal energy is dissipated laterally, while only a minimal fraction conducts axially—ensuring the substrate remains safely below its damage threshold. One practical approach is to modify high-reflectivity SiO_2_ nanofibers with a high-conductivity core—an architecture that effectively imparts anisotropic thermal conduction.

We performed finite-element thermal-transfer simulations (COMSOL Multiphysics) to compare thermal conduction in pure SiO_2_ nanofibers with that in core–shell nanofibers containing a high-conductivity core. It should be noted that the interfacial thermal resistance (ITR) between the BN core and the SiO₂ shell was not explicitly included in our model, as it can be negligible at the micrometer scale^39–43^. (Details on ITR, heat conduction, thermal radiation, and convective effects are provided in Supplementary note). The simulated thermal evolution reveals a fundamental difference between the two architectures as shown in Fig. 1b and Supplementary Fig. 1a (see Supplementary Fig. 2 for more detailed Heat Transfer Simulation). This macroscopic anisotropy originates from the fiber-level architecture. Within the membrane, the individual core-shell fibers are predominantly oriented in-plane. Phonon transport along the high-conductivity BN core therefore occurs primarily along the fiber axis, which collectively establishes efficient in-plane (radial) heat dissipation. Conversely, heat transfer through the membrane thickness (axial direction) is impeded both by the intrinsically low thermal conductivity of the SiO_2_ shell and, more critically, by the inter-layer pores and interfaces within the stacked fibrous network. The core–shell architecture channels heat primarily in the radial direction and significantly impedes its axial propagation. In pure SiO_2_ fiber mats, thermal distribution remains relatively uniform due to silica’s intrinsically low thermal conductivity. This homogeneous behavior is further governed by structural factors such as the fiber in-plane packing density and interlayer spacing^44^ (see Supplementary Fig. 1b for a schematic illustration of solid nanofibers). With the addition of a high-conductivity core, however, radial heat flux is greatly enhanced, ensuring more effective interception of the laser energy before it can reach the substrate (Fig. 1c). In summary, the core–shell nanofiber design simultaneously suppresses axial thermal conduction and promotes rapid radial dispersion of incident laser energy, thereby minimizing the risk of substrate damage. This anisotropic thermal management strategy meets the stringent requirements of high-energy laser environments and opens new avenues for advanced protective coatings.

### Core-shell structural features

**Fig. 2:**
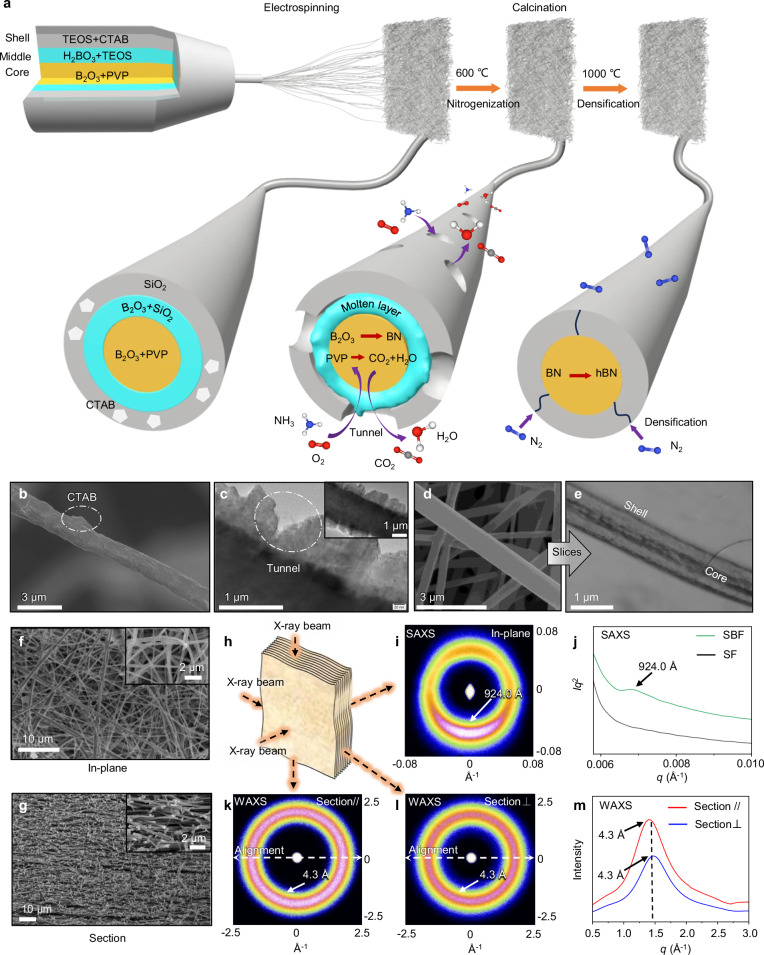
Fabrication and characterization of the core–shell structured nanofiber membrane (SBF). **a** Schematic illustration of the fabrication process of core–shell structured fibers, highlighting major chemical reactions and structural changes in the nanofibers. **b** SEM image of the rough fiber surface prior to calcination. **c** TEM image showing surface channels after preliminary calcination. **d** SEM image of fiber surface following high-temperature calcination. **e** TEM image of cross-sectioned fibers after high-temperature calcination. **f**–**g** SEM images illustrating the in-plane and cross-sectional structures of the SBF membrane, respectively. **h** Schematic diagram of the X-ray scattering experimental setup. **i**–**j** In-plane SAXS patterns and corresponding peak positions of the SBF membrane; for comparison, the scattering pattern of a pure SiO_2_ fiber membrane is included in **j**. **k**–**m** WAXS patterns from two different interface orientations, showing peaks within this scattering range. All fibers shown in Fig. 2 were fabricated under the optimal precursor feed ratio of S (silica) to B (boron nitride) = 0.6:0.4.

To incorporate a high-thermal-conductivity BN core within SiO_2_ nanofibers while achieving a stable core–shell architecture, we developed a tri-channel electrospinning technique that produces fibers with distinct shell, intermediate, and core layers in one step. The as-spun composite fibers were then subjected to a carefully designed multi-stage calcination under different atmospheres to convert the core material into a crystalline BN phase and finalize the core–shell structure. As illustrated in Fig. 2a, three precursor solutions were used: a silica sol (tetraethyl orthosilicate, TEOS) containing cetyltrimethylammonium bromide (CTAB) as a porogen for the outer shell; a mixture of boric acid (H₃BO₃) and TEOS for the intermediate layer to promote chemical compatibility between core and shell; and a solution of boron oxide (B₂O₃) with poly(vinylpyrrolidone) (PVP) to form the core (the polymer aids in viscosity control and uniform fiber drawing). This configuration yielded as-spun fibers consisting of a SiO₂/CTAB shell, a B₂O₃–SiO₂ transitional layer, and a B₂O₃/PVP core. Because of the high organic content (CTAB and PVP) in these composite fibers, they required calcination to remove organics and render the structure fully inorganic. In the initial low-temperature pre-calcination step (≈ 600 °C in a mixed ammonia/oxygen atmosphere), the CTAB decomposed, creating porosity in the SiO_2_ shell by forming open channels that connect the fiber core to the exterior. Simultaneously, the boron species in the intermediate and core layers began to convert to BN, while the PVP in the core pyrolyzed; the gaseous byproducts (H₂O, CO₂ and NH_3_) escaped through the shell’s new channels. Notably, the transitional B₂O₃–SiO₂ layer, which becomes molten at these temperatures, acted as a temporary barrier around the core—its permeability could be tuned by adjusting the H₃BO₃:TEOS ratio, thereby controlling gas release and preventing structural collapse of the core. In the subsequent high-temperature calcination (1000–1200 °C under N₂), the boron nitride fully crystallized into hexagonal BN within the fiber core. At these elevated temperatures, the core also densified and shrank, causing the earlier-formed shell channels to close (concurrent with partial crystallization of the SiO_2_ matrix). Through this controlled sequence, we obtained core–shell nanofibers with a well-crystallized BN core and a robust SiO_2_ shell (SBF).

We conducted extensive structural characterization at each stage of this process to validate the core–shell architecture. Scanning electron microscopy (SEM) of the as-spun fibers (Fig. 2b and additional SEM images in Supplementary Fig. 3) revealed a rough, non-uniform surface arising from the dense CTAB coating on the fiber exterior. This roughness reflects the presence of abundant porogen, which is critical for generating high shell porosity and subsequent channel formation. After the low-temperature calcination, transmission electron microscopy (TEM) images of fibers treated at 600 °C (Fig. 2c) show numerous nanochannels perforating the SiO_2_ shell, confirming that CTAB decomposition successfully introduced an interconnected pore network. To further validate the porosity, N₂ physisorption measurements were conducted (see Supplementary Fig. 4 for BET evolution), showing the development of well-defined mesopores (1-3 nm) with a high specific surface area of ≈ 400 m² g^−1^. These results support the TEM observations and confirm the successful formation of mesopores following CTAB removal. Following the high-temperature calcination, the fiber surfaces became very smooth (SEM, Fig. 2d), indicating that the SiO_2_ shell had densified and the surface pores closed. The fiber diameter contracted by ≈ 15–20% during the calcination process, primarily due to the removal of organic components and densification of the shell, resulting in a more compact and stable core-shell structure. Cross-sectional TEM imaging of the fully calcined fibers (Fig. 2e) clearly verifies a distinct core region encased by the SiO_2_ shell, demonstrating that the intended core–shell structure was achieved without collapse or voids in the core.

To further substantiate the core-shell morphology and confirm the crystallinity and structural integrity of the BN core, we performed high-resolution TEM (HRTEM) and energy-dispersive X-ray spectroscopy (EDS) analysis on samples with different core-shell ratios. Cross-sectional HRTEM images (Supplementary Fig. 5a) reveal a well-defined core-shell structure, with the core region showing regularly aligned lattice fringes indicative of the highly crystalline nature of the BN phase. A magnified view of the core (Supplementary Fig. 5b–c) shows an interplanar spacing of ≈ 0.33 nm, corresponding to the (0002) lattice plane of hexagonal boron nitride. The selected-area electron diffraction pattern (SAED, Supplementary Fig. 5d) further confirms the high crystallinity and structural integrity of the BN core. EDS elemental mapping (Supplementary Fig. 5e–h) clearly reveals that the core is predominantly composed of boron (B) and nitrogen (N), confirming the presence of a BN-rich phase, while the shell consists primarily of silicon (Si) and oxygen (O), consistent with SiO_2_. Importantly, no detectable boron or nitrogen signals are observed in the shell region, confirming the sharp compositional transition between the BN core and SiO_2_ shell and ruling out the presence of boron or boron-rich non-crystalline phases in the shell. These results provide definitive evidence that the core-shell structure is well-formed, with the BN core and SiO_2_ shell clearly distinguished and free from contamination.

In parallel, complementary spectroscopic analyses corroborated the compositional transformations of the fibers during calcination. Fourier transform infrared (FTIR) spectra (see Supplementary Fig. 6a for FTIR evolution) show the disappearance of peaks associated with organic functional groups (from CTAB and PVP) and the emergence of new bands attributable to B–N bonds as the calcination progressed, indicating the formation of BN inside the fibers. Thermogravimetric analysis (TGA, see Supplementary Fig. 6b for TGA evolution) further supports this evolution, showing weight loss steps corresponding to the decomposition of CTAB and PVP in the early calcination stages. Likewise, X-ray photoelectron spectroscopy (XPS, see Supplementary Fig. 7 for detailed XPS data) shows a clear reduction of carbon- and oxygen-containing species and the appearance of boron and nitrogen signals in the final calcined fibers. Collectively, these observations confirm the successful removal of organic components and the formation of a purely inorganic SiO_2_/BN core–shell structure.

The SBF membrane itself exhibits a hierarchical multilayer morphology. In-plane SEM views (Fig. 2f) reveal that individual core–shell fibers are randomly oriented and interwoven without any binder or cross-linking, forming a planar fibrous network. Cross-sectional SEM (Fig. 2g) shows that these fibers are densely stacked through the thickness of the membrane, creating a tightly intertwined multi-layer structure. In essence, the membrane behaves as a cohesive laminate of nanofiber layers. This architecture greatly enhances interlayer shear strength, ensuring robust mechanical cohesion and strong adhesion of the membrane to its substrate.

Finally, we quantitatively probed the membrane’s structural ordering using small- and wide-angle X-ray scattering (Fig. 2h). The in-plane small-angle X-ray scattering (SAXS) pattern (Fig. 2i) of the SBF membrane exhibits a pronounced correlation peak corresponding to a real-space distance of ≈ 9 μm. This feature is absent in pure SiO_2_ nanofiber membranes (Fig. 2j and see Supplementary Fig. 8 for further interpretation of SAXS results of SF), confirming that it arises from the presence of the crystalline BN cores and reflecting an average in-plane fiber spacing on the order of a few micrometers. Consistently, powder X-ray diffraction (PXRD, see Supplementary Fig. 6c for PXRD evolution) of fibers at different calcination stages shows the progressive appearance of characteristic hexagonal BN peaks, verifying the formation of the crystalline BN phase in the core. When the X-ray beam was directed edge-on to the membrane (side-view), the 2D scattering pattern appeared as a uniform ring, indicating an isotropic in-plane fiber orientation with no preferred alignment. Moreover, wide-angle X-ray scattering (WAXS) patterns collected from two orthogonal directions (Fig. 2k, l) show identical diffraction features (differing only in intensity) – notably, both exhibit a reflection corresponding to a periodicity of ≈ 43 μm. The presence of this ≈ 43 μm structural feature in all orientations suggests a consistent interlayer spacing throughout the multilayered membrane. Taken together, these scattering results conclusively validate the designed core–shell multilayer architecture and demonstrate the enduring crystalline integrity of the BN core within the SiO_2_ nanofibers.

### Optical performance characteristics

**Fig. 3:**
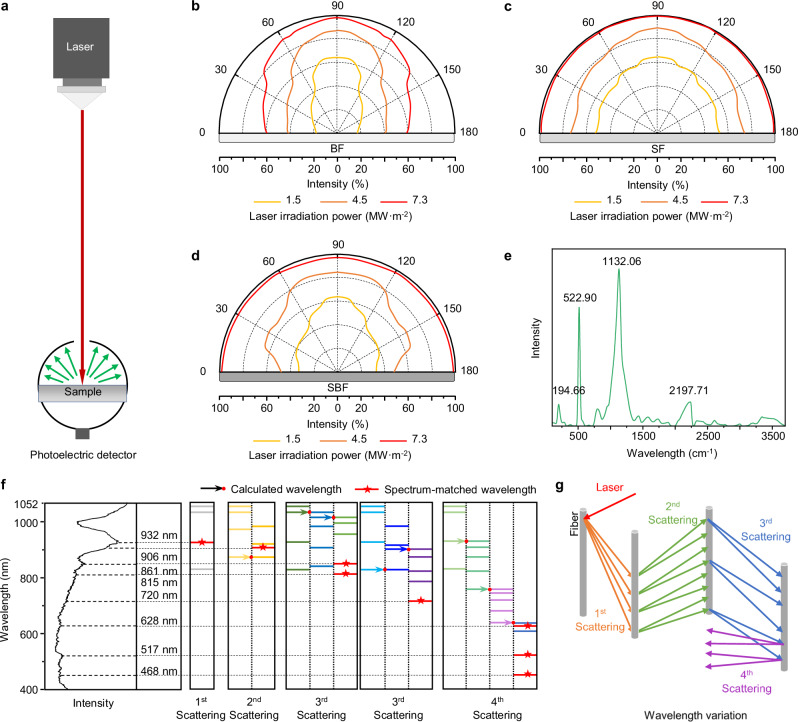
Optical characterization of the core–shell structured nanofiber membrane. **a** Schematic diagram of the optical performance testing setup. **b**–**d** Front-side scattering intensity results for BF membrane, SF membrane, and SBF membrane, respectively. **e** Raman spectrum of the SBF membrane. **f** Spectral analysis and wavelength shift calculations for the SBF membrane under laser irradiation. **g** Schematic illustration demonstrating the enhanced scattering capability of the SBF membrane. Different colors indicate different numbers of scattering in **f**, **g**.

The SBF membrane is derived from a conventional SF membrane by incorporating a BN core. This dual-phase architecture, intended primarily for improved thermal management, also modifies the membrane’s optical response. To elucidate SBF’s laser attenuation mechanisms, we characterized its optical performance via front-side scattering and Raman spectroscopy. Specifically, front-face scattering intensity was quantified using an integrating sphere, while the spectrum of the scattered light was recorded with a spectrometer (Fig. 3a).

The first laser attenuation mechanism arises from the disruption of beam directionality, which effectively reduces specular reflection. To quantify this effect, each membrane was positioned at the entrance of an integrating sphere and irradiated with a fixed-power laser (Fig. 3a). The intensity of light scattered into the sphere interior was subsequently recorded. As shown in Fig. 3b, the BF membrane exhibits predominantly specular reflection, producing a highly directional scattering pattern that limits lateral energy dispersion—even at elevated power levels—despite its inherently high reflectivity. In contrast, the SF membrane displays a largely diffuse scattering profile that becomes increasingly isotropic with rising laser power (Fig. 3c). The SBF membrane exhibits an intermediate, power-dependent behavior (Fig. 3d): at low power, its scattering is largely specular, but transitions to diffuse as the incident power increases. This shift is attributed to thermally induced changes in the refractive index of the SiO_2_ shell, which enhance angular energy dispersion and suppress localized absorption in both SF and SBF membranes. Importantly, at high laser power, the SBF membrane achieves near-complete reflection of the incident 1064 nm beam. This reflectivity continues to improve with increasing power, highlighting the membrane’s enhanced optical scattering capability and dynamic stability under high-flux conditions.

A second attenuation mechanism involves Raman scattering in the SiO_2_ shell, which shifts photon wavelengths by fixed energy offsets. Using a 1064 nm excitation, we recorded the Raman spectrum of SBF (Fig. 3e) and identified multiple cascaded scattering orders. These cascades produce photons at various wavelengths whose intensities overlap to give the observed spectral distribution (Fig. 3f, left). The right panel of Fig. 3f provides a clearer demonstration of the multi-order Raman scattering process by showing how the experimentally detected wavelengths correspond to calculated Raman transitions. Specifically, the 932 nm wavelength observed in the first-order Raman scattering spectrum serves as the input for calculating the second-order Stokes and anti-Stokes shifts, yielding the 906 nm wavelength that is experimentally verified. This calculation-experiment correlation continues through the third and fourth order transitions, with all predicted wavelengths consistently detected in the laser-irradiated scattering spectrum. 1064 nm light first generates first-order anti-Stokes photons at 1049, 1024, 932, and 812 nm; the 812 nm photons then generate second-order Stokes photons at 884, 912, and 967 nm; finally, 884 nm photons produce third-order anti-Stokes light at 872, 846, 803, and 739 nm. This multi-step Raman process effectively broadens the spectrum of scattered light. Notably, this wavelength-modulating behavior is seen in SBF (via its SiO_2_ shell) and in pure SF, but not in BF (see Supplementary Fig. 9 for reflectance spectra of SF, SBF, and BF), confirming that the SiO_2_ component is responsible for the effect.

In summary, these results indicate that incident laser energy is dissipated through complex, multi-order scattering within the fibrous network, altering both the beam’s propagation direction and its spectral content (Fig. 3g). A portion of the energy is also converted to heat in the surroundings, facilitated by the Raman scattering mechanism within the SiO_2_ shell, which converts absorbed photon energy into lattice vibrations, enhancing thermal radiation to the environment.

To quantitatively assess this energy conversion, we estimated the contribution based on the well-established Raman scattering cross-section of silica, which is on the order of 10^−30 ^cm^2^ steradian^−1^ molecule^−1^ as confirmed by experimental measurements^45^. In our work, the high surface area and mesoporous network of the SiO₂ shell provide a large effective scattering volume. Based on the relative intensities of the pump (1064 nm) and the first-order Stokes (∼932 nm) peaks in our recorded spectra (Fig. 3e, f) and by applying the standard relationship for Raman scattering intensity to the aforementioned cross-section and our experimental parameters we estimate that ≈ 1–5% of the incident laser energy is channeled into optical phonons of the SiO_2_ network via the multi-order Raman scattering process. Although this fraction is modest, this inelastic pathway provides a direct, quantitative mechanism for the initial photothermal conversion.

Importantly, this behavior underscores the role of the core-shell architecture, where the SiO₂ shell and fibrous structure govern optical reflection, while the embedded BN core provides complementary thermal management. Compared to an SF membrane, the SBF retains its efficient diffuse scattering and Raman-based wavelength-shifting capabilities, which underlie its superior performance in high-energy laser protection. This synergistic effect between the optical properties of the SiO_2_ shell and the thermal dissipation functionality of the BN core ensures exceptional performance under high-energy laser exposure, combining both efficient optical shielding and enhanced heat dissipation.

### Thermal performance characteristics

**Fig. 4:**
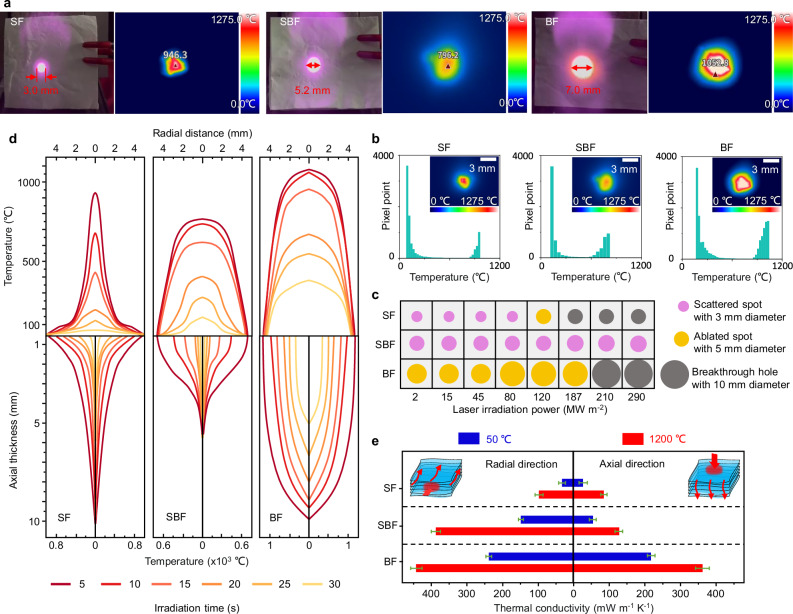
Thermal management performance of core–shell structured nanofiber membrane. **a** Laser spot sizes and central temperatures for SF membrane, SBF membrane, and BF membrane under high-energy laser irradiation. **b** Statistical analysis of temperature distribution across 4000 pixels for the three fiber membranes, where the pixel value indicates the number of pixels at the same temperature. **c** Comparative laser spot size data for three membranes under different high-energy laser power levels. **d** Temporal evolution of temperature distribution in radial and axial directions for the three fiber membranes under laser irradiation. **e** Thermal conductivity comparison of the three fiber membranes at high and low temperatures in both radial and axial directions. Data are presented as mean ± standard deviation, derived from *n* = 12 independent measurements for each condition.

To evaluate the thermal management afforded by the core–shell architecture, the spot size and temperature distribution were measured under controlled high-energy laser irradiation. Under identical irradiation conditions (14 kW cm⁻², fixed focus), the SBF membrane produces an irradiated spot of 5.2 mm, versus 3.0 mm for the SF membrane and 7.0 mm for the BF membrane (Fig. 4a). The enlarged thermal emission area for SBF reflects the combined action of surface thermal diffusion and solid conduction, which enhances overall scattering and visually broadens the irradiated spot.

Infrared thermography further shows that the SBF surface remains cooler under irradiation than either SF or BF, indicating more effective radial thermal spreading. The enhanced Raman scattering in the SiO_2_ shell contributes to more efficient heat rejection, which is reflected in the lower front-surface temperature of SF and SBF under identical irradiation conditions, as shown in Fig. 4a. This improved thermal dissipation arises because SiO_2_ inherently reflects more incident laser energy than BN; thus, the embedded BN core preferentially conducts thermal radially away from the hotspot, limiting the central temperature rise in the SBF structure.

Statistical analysis of infrared thermal images (Fig. 4b) reveals distinct temperature distributions for each membrane. The SF membrane exhibits a sharp high-temperature peak, indicative of localized thermal accumulation; by contrast, the BF membrane shows a broader high-temperature distribution, indicating more extensive lateral thermal spread. Notably, the SBF membrane lacks a sharp peak, instead showing a flattened high-temperature plateau with a lower maximum temperature, which confirms its superior thermal dissipation capability.

We also examined the effect of increasing laser power on thermal stability (Fig. 4c). The SBF membrane maintains a nearly constant spot size (≈ 3 mm) as power increases, showing only a slight expansion to 3.4 mm even at a power density of 29 kW cm⁻², highlighting its exceptional thermal resilience. In contrast, the SF membrane maintains a 3 mm spot only below ≈ 8 kW cm⁻²; beyond this threshold the spot rapidly expands to about 5 mm with burning and eventual perforation. The BF membrane’s ablation area gradually widens from 7 mm to 10 mm before abrupt failure at ≈ 21 kW cm⁻². These observations underscore that the SBF retains structural integrity under intense thermal shock, a critical feature for effective protective materials.

Time-resolved thermal mapping along the radial and axial directions (Fig. 4d and temperature evolution curves in Supplementary Fig. 10) further delineates the heating profiles. Under 1064 nm irradiation (0.8 kW cm⁻²), the SF membrane exhibits a star-like profile: radially, temperature drops sharply toward the periphery before rising over time, while axially it falls rapidly away from the heated face and then slowly recovers. This convergent profile promotes internal heat accumulation, which can be detrimental to insulation performance. By contrast, the BF membrane shows a disc-like profile: temperatures decrease gradually with both radius and depth, remaining above 1000 °C even 5 mm below the surface. Although this expansive profile ensures high-temperature stability of the BF membrane itself, it is less desirable for protecting substrates with limited thermal tolerance. The SBF membrane, however, exhibits a unique seed-like profile: radially it resembles the BF (extended hot region), but axially the temperature plunges quickly to near ambient by 5 mm depth. This hybrid distribution preserves the SBF’s structural stability while providing effective thermal protection to the underlying substrate.

The exceptional thermal behavior of the SBF derives from its anisotropic fibrous structure. In densely packed membranes, fibers form continuous solid-state conduction pathways. Consequently, the radial thermal conductivity of SBF approaches that of pure BN (increasing with core diameter), reflecting efficient heat transport along the high-conductivity BN core (Fig. 4e). By contrast, axial heat flow is dominated by the low-conductivity SiO_2_ shell, rendering the axial thermal conductivity similar to that of pure SiO_2_. Directional thermal diffusivity measurements performed via laser flash analysis (LFA) confirm that SBF exhibits superior radial heat dissipation, with radial thermal diffusivity increasing at high temperatures compared to SF and BF (see Supplementary Fig. 11 for thermal diffusivity data). This anisotropic conduction is mirrored in thermal expansion (see Supplementary Fig. 12 for Thermal Conductivity and Expansion Coefficient evolution): the SBF’s radial expansion coefficient remains low (comparable to SF), whereas its axial expansion coefficient is higher (comparable to BF). Collectively, these properties bestow the SBF membrane with outstanding intrinsic thermal stability and insulation performance.

### High-energy laser protection performance characteristics

**Fig. 5:**
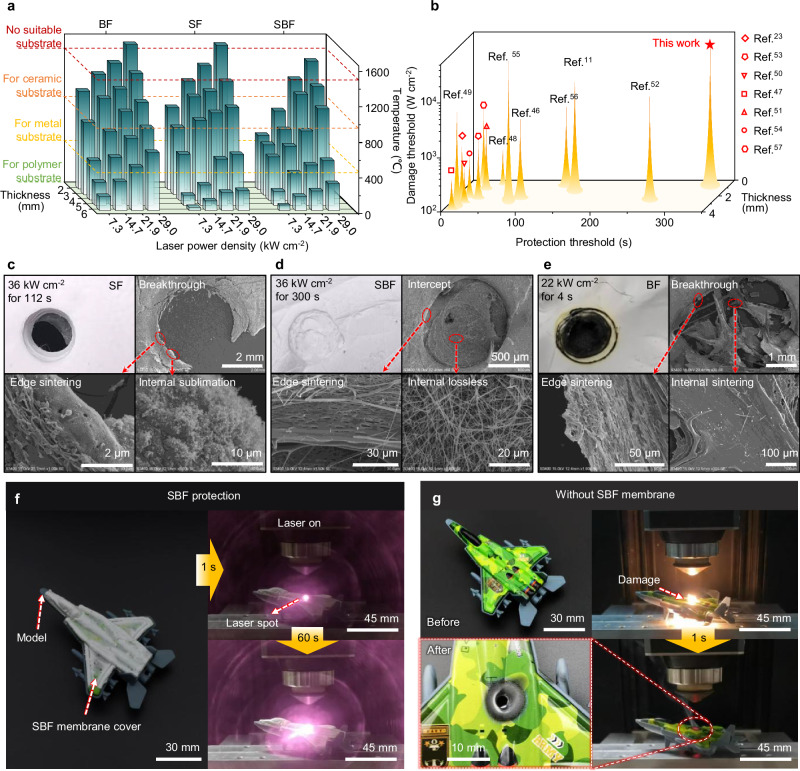
High-energy laser protection performance of core–shell structured nanofiber membrane. **a** Back-surface temperature profiles of fiber membranes with varying thicknesses under different laser power conditions, and corresponding suitable substrate materials. **b** Comparative analysis of laser protection thresholds between this study and previously reported materials at different membrane thicknesses. Laser damage threshold and protection threshold of super white boron nitride aerogel (BN aerogel)^11^, tantalum disilicide and zirconium disilicide-modified carbon fabric-reinforced phenolic composite (TaSi_2_/ZrSi_2_/C-Ph)^51^, graphite/SiO_2_ (GA/SiO_2_)^52^, plasma-sprayed La_1-*x*_Sr_*x*_TiO_3+*δ*_ coating (LST)^53^, glass fiber-reinforced phenolic composites (GF/Ph)^23^, zirconium diboride particulates and silicon carbide whiskers with zirconia composite coating (ZrO_2_/ZrB_2_/SiC)^54^, zirconium carbide-modified short-carbon-fiber-reinforced phenolic-resin (ZrC/CF/BPF)^55^, laminated SiO_2_-graphite/SiO_2_/Cu composites (SGS-Cu)^56^, ZrB_2_/Cu composites (ZrB_2_/Cu)^57^, boron-modified phenolic resin (BPF)^58^, glass fiber-reinforced epoxy metamaterials (GF/EP)^59^, three-dimensional carbon fiber-reinforced zirconium boride and silicon carbide composite (C_f-PyC_/ZrB_2_-SiC)^60^, a rare earth titanate (Y, La) ceramic coating (RE_2_Ti_2_O_7_)^61^ and aluminum-based composite coating reinforced with aluminum boron carbide particles (Al_3_BC/Al)^62^. Reproduced with permission from refs. ^11, 61^ (copyright 2023, 2025 John Wiley and Sons), refs. ^51, 52, 54–60, 62^ (copyright 2020, 2017, 2021, 2019, 2022, 2014, 2019, 2023, 2024, 2025 Elsevier), ref. ^53^ (copyright 2017 American Chemical Society), ref. ^23^ (copyright 2022 Multidisciplinary Digital Publishing Institute). **c**–**e** SEM micrographs showing microscopic damage morphology of SF, SBF, and BF membranes, respectively, after high-energy laser irradiation. **f** Performance of a model protected by a SBF membrane under high-energy laser irradiation. **g** High-energy laser penetration process observed after removal of SBF membrane protection.

The high-energy laser protection performance of the SBF membrane was evaluated by taking advantage of its strong optical scattering and anisotropic thermal conduction. Substrate protection was assessed by monitoring the back-surface temperature of membranes of defined thickness under varying laser power densities (Fig. 5a). At 29 kW cm⁻², a 2 mm-thick BF membrane reached ≈ 1647 °C on the rear surface—far above the tolerance of most structural materials. In contrast, a 2 mm SBF membrane held the back-surface temperature to ≈ 1350 °C, a level within the continuous service limit of advanced ceramics (e.g., silicon carbide). Increasing the SBF thickness to 5 mm under identical irradiation further reduced the rear temperature to ≈ 312 °C, safely within the operating range of high-performance polymers (e.g., PEEK, PPS). These results demonstrate that by tuning the SBF membrane thickness, a wide range of substrate materials can be effectively protected.

A comparative analysis of damage thresholds versus thickness was then performed for various protective materials (Fig. 5b, Supplementary Table 1, Supplementary Movies 1 and 2). The SBF membrane achieved notably longer protection durations and higher damage thresholds at a given thickness. Remarkably, even under the maximum laser power available in our setup (36 kW cm⁻²), the SBF membrane incurred only superficial surface ablation, suggesting its true damage threshold lies beyond this limit. By contrast, many reported composite protectors require much greater thickness (and hence higher density) to achieve comparable protection and typically exhibit lower threshold energies, highlighting the performance advantage of the SBF design.

SEM was used to study the failure morphologies of the SF, SBF, and BF membranes after laser irradiation (Fig. 5c–e). Under 36 kW cm⁻², the SF membrane failed catastrophically in ∼112 s. SEM reveals globular molten SiO_2_ and fine sublimation residues along the crater rim, characteristic of abrupt melting and vaporization. In contrast, after 300 s at 36 kW cm⁻², the SBF membrane exhibited only shallow surface pitting; SEM images show that melting was confined to the pit edges, with the core–shell fibers remaining intact beneath. This suggests a gradual, self-limiting failure mode, possibly because initial surface ablation enhances scattering and insulation for continued exposure. This superior performance is attributed to the synergistic effects of the core-shell design. The SiO₂ shell provides effective optical shielding, the BN core enables efficient radial heat dissipation, and the fiber network offers mechanical buffering. These combined mechanisms help SBF outperform SF (lacking heat spreading) and BF (lacking effective optical shielding). The BF membrane failed most rapidly (∼4 s at 22 kW cm⁻²), with SEM showing pervasive melting at both the pit edge and base, consistent with an uncontrolled melt-through failure. In contrast, the SBF membrane resists catastrophic breakdown during surface melting, with the softened SiO_2_ shell acting as a self-sealing barrier, while the BN core maintains thermal transport, preventing thermal runaway and preserving structural integrity.

Finally, the practical efficacy of the SBF membrane was demonstrated by shielding a polymer substrate under high-energy laser exposure (Fig. 5f, Supplementary Movie 3 and Fig. 5g). A 2 mm SBF membrane was applied to an ABS plastic model and irradiated at 15 kW cm⁻². A pronounced diffuse scattering was observed on the membrane surface, and notably the ABS substrate remained undamaged even after 60 s of continuous exposure. In sharp contrast, removing the SBF protection led to immediate penetration of the ABS under the same conditions, with through-holes appearing in the material (Fig. 5g). These observations confirm that the SBF membrane’s exceptional reflectivity and thermal insulation effectively protect polymer substrates from intense laser irradiation.

To simulate more realistic operational conditions, we also conducted pulsed laser irradiation tests. As shown in Supplementary Fig. 13, the SBF membrane exhibits excellent resistance to pulsed laser irradiation, with the BFs being penetrated at 1600 W, SFs at 2500 W, and the core-shell SBFs demonstrating only a ≈ 2.4 mm deep damage crater at 3000 W and requiring 3600 W for full penetration. This highlights the membrane’s superior defensive capability against intense pulsed laser attacks. These pulsed laser experiments demonstrate that the SiO_2_@BN core-shell membrane retains its critical properties, including high reflectivity, strong thermal anisotropy, and reliable laser protection, under extreme pulsed laser conditions. This confirms the material’s potential for advanced laser protection applications in real-world high-energy laser environments.

In addition to the exceptional optical, thermal, and laser protection properties demonstrated by our SiO_2_@BN core-shell nanofibers, the structural design of the fibers was further optimized by adjusting the S (silica):B (boron nitride) precursor feed-rate ratio during electrospinning. This modification allowed us to systematically vary the core-shell ratio and investigate its effect on the material’s performance in terms of reflectivity, thermal conductivity, and laser damage tolerance. As shown in Supplementary Fig. 14a, the hemispherical reflectivity increases with the SiO_2_ shell thickness, reaching nearly 100% at a S:B ratio of 0.5:0.7, beyond which it plateaus, indicating complete coverage by the SiO_2_ shell. Thermal conductivity measurements reveal that the optimal thermal anisotropy occurs at a S:B ratio of 0.6:0.4, where radial conductivity is maximized while axial conductivity remains low (Supplementary Fig. 14b). This ratio enables effective in-plane heat dissipation, with the BN core playing a key role in radial heat transport. As the BN core diameter decreases, the back-side temperature increases due to reduced radial heat dissipation, highlighting the critical role of the BN core in thermal management (Supplementary Fig. 14c). The laser damage threshold shifts with the core-shell ratio: at lower power densities (<20 kW cm^−2^), thicker SiO_2_ shells (S:B = 0.5:0.7) offer better protection due to enhanced reflectivity, while at higher power densities (>30 kW cm^−2^), the S:B ratio of 0.6:0.4 provides the best damage tolerance, offering a balance of reflective shielding and efficient heat dissipation (Supplementary Fig. 14d).

These results confirm that the S:B ratio of 0.6:0.4 used in Figs. 1–5 is optimal, balancing optical reflectivity, thermal conductivity, and laser damage tolerance. Radial conductivity measurements showing preferential heat flow through the BN core over the SiO_2_ shell directly demonstrate the dominant role enabled by its continuous, percolating structure, which minimizes interfacial thermal resistance and enhances heat dissipation. Additionally, the percolating nature of the BN core, as opposed to segmented or interrupted cores, minimizes interfacial thermal resistances, further enhancing heat dissipation. By tuning the core-shell ratio, we have optimized both the thermal and optical properties of the material, making it highly suitable for high-power laser protection applications.

### Mechanical property characteristics

**Fig. 6:**
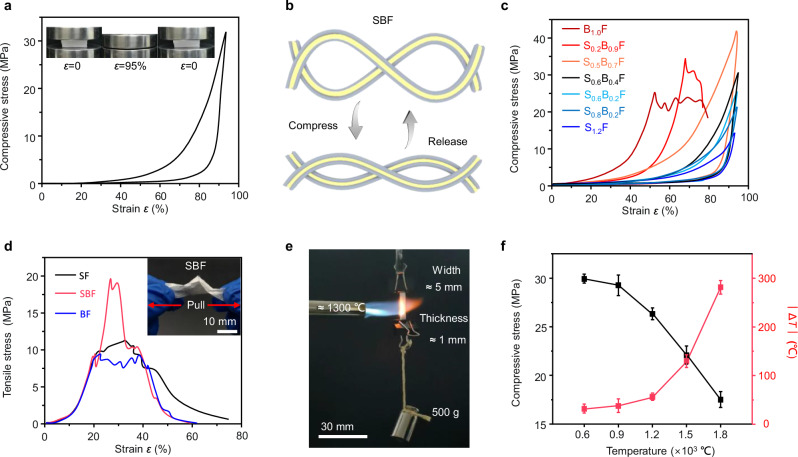
Mechanical properties of core–shell structured nanofiber membrane. **a** Compressive stress-strain curves for SBF membranes; inset shows the membrane morphology at 95% compression strain. **b** Schematic illustration showing morphological changes of core–shell structured fibers under compression. **c** Comparative compressive strength analysis for core–shell structured fibers with varying core diameters and shell thicknesses. **d** Tensile stress-strain curves for SF, SBF, and BF membranes; inset highlights excellent tensile strength of core–shell fibers. **e** Tensile strength of SBF membrane under high-temperature conditions. **f** Mechanical and thermal insulation property balance of SBF membrane with increasing temperature. Data are presented as mean ± standard deviation derived from *n* = 5 independent measurements.

Compression tests reveal that a 4 mm-thick SBF membrane exhibits high compressibility, sustaining up to 95% strain with complete thickness recovery upon unloading (Fig. 6a, inset). This behavior originates from the synergistic interaction between the core and shell components. In the initial linear elastic regime (up to ≈ 40% strain), the porous amorphous SiO_2_ shell acts as a compliant matrix that absorbs and distributes the compressive load^46^. Simultaneously, the continuous crystalline BN core functions as an internal scaffold, progressively bearing load during densification and providing the restoring force necessary for elastic recovery^47^. This core–shell synergy yields exceptional tolerance to extreme strain^41,48^. Unlike monolithic crystalline fibers, the SBF sustains ≈ 95% compression without fracture, a behavior attributed to the rigid BN core, which provides robust mechanical support and enables recovery through synergistic core-shell interaction (Fig. 6b). The macroscopic deformation observed during compression primarily results from network-level structural rearrangement, including pore closure and slippage at fiber junctions, with minimal change in the length of individual fibers. This mechanism allows the SBF membrane to accommodate extreme strain without fracture, highlighting the unique synergy between the BN core and the SiO_2_ shell. As shown in Fig. 6c, increasing the SiO_2_ shell thickness extends the elastic region and lowers the compressive stress at a given strain, whereas enlarging the BN core increases the stress. Beyond a critical core diameter, brittle fracture occurs, underscoring the trade-off between silica’s compliance and BN’s rigidity.

Impact tests demonstrate the membrane’s excellent cushioning performance. A 1 mm-thick SBF membrane withstands the drop impact of a 50 g mass from 1.5 m (see Supplementary Fig. 15 for impact resistance test diagram) without damage, highlighting its superior shock absorption capability and ability to protect underlying substrates from sudden impacts.

Under tensile loading, the SBF membrane demonstrates a distinctive two-stage failure process that enhances its damage tolerance (Fig. 6d). The initial stress drop at ≈20% strain corresponds to the sequential fracture of the rigid BN core filaments, while the subsequent stress recovery plateau (≈20 MPa) results from effective load transfer to the continuous SiO_2_ shell network^49^. This staged failure mechanism, analogous to fiber bridging in composite materials, allows the membrane to maintain structural integrity and dissipate energy progressively through shell deformation and fiber pull-out, ultimately achieving ≈ 25% failure strain^50^. The coordinated response of core and shell during tensile deformation significantly prolongs the load-bearing phase and improves the effective toughness compared to homogeneous ceramic materials.

At elevated temperatures, the SBF membrane maintains robust mechanical integrity. In a 1300 °C flame test, a 5 × 1 mm membrane strip supports a 500 g load for 2 min without damage (Fig. 6e and Supplementary Movie 4). Prolonged heating does not significantly reduce the tensile strength of the fibers. Figure 6f shows compressive strength and thermal insulation as functions of temperature: compressive strength gradually decreases with increasing temperature, whereas thermal insulation remains largely unchanged. These results indicate that an optimal balance of mechanical strength and insulation is reached near ∼1500 °C, beyond which both properties rapidly degrade.

### Durability characteristics

**Fig. 7:**
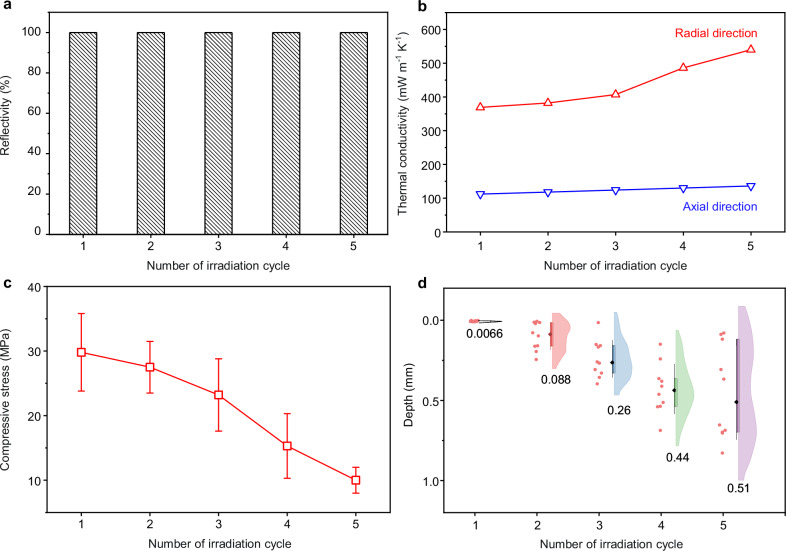
Durability properties of core–shell structured nanofiber membrane. **a** reflectivity,b thermal conductivity along two principal directions, **c** compressive stress, and **d** depth of laser-induced damage. Data in **c** and **d** are presented as mean ± standard deviation derived from *n* = 5 and *n* = 10 independent measurements, respectively.

In addition to the impressive optical and thermal performance, the long-term durability of the SiO_2_@BN core-shell nanofiber membrane under various environmental conditions is critical for its practical applications in high-energy laser protection. Beyond the evaluations under environmental aging, the durability of the SBF membrane under repeated high-energy laser exposure is also essential for assessing its long-term performance in practical applications. To evaluate its resilience, we subjected the SBF membrane to cyclic laser irradiation tests, simulating the repeated thermal stress encountered in high-energy laser environments. As shown in Fig. 7, the cyclic laser irradiation tests further reveal the material’s excellent durability and functional stability. Throughout the repeated thermal cycles, the SBF sample maintains its original high reflectivity, confirming the structural integrity of the silica shell (Fig. 7a). The thermal transport properties evolve favorably: the radial thermal conductivity at 1000 °C increases gradually (Fig. 7b), while the axial value remains stable, thereby enhancing the overall thermal anisotropy—a beneficial trait for in-plane heat spreading under repeated thermal loading. A degradation in compressive properties is observed after repeated laser cycling, arising from the accelerated exacerbation of structural rigidity defects likely caused by boron nitride crystal degradation at high temperatures (Fig. 7c). Notably, despite the progressive increase in laser-induced damage depth with each irradiation cycle, the absolute depth remains comparatively low overall, confirming that the material retains excellent laser protection performance even after repeated exposure (Fig. 7d).

Beyond the evaluations under repeated high-energy laser exposure, the durability of the SBF membrane under environmental aging is also essential for assessing its long-term performance in practical applications. To further evaluate its resilience, we subjected the SBF membranes to accelerated aging tests under high-humidity, oxidative, and thermocyclic conditions. As shown in the Supplementary Fig. 16, the SBF membranes demonstrate remarkable stability across all tested environments. The reflectivity of the SBF membrane remained unchanged at nearly 100% after prolonged exposure to high humidity, a high-oxygen atmosphere, and multiple thermal cycles. This stability is attributed to the inherent chemical inertness of the silica shell (see Supplementary Fig. 16a for reflectivity data). The thermal conductivity of the SBF membrane at 50 °C showed no significant change after aging in humid or oxidative environments. Conversely, a slight increase was observed following five thermal cycles at 800 °C, which is likely due to a phase transformation of the boron nitride crystals at high temperature (see Supplementary Fig. 16b for specific thermal conductivity data). The compressive stress of the SBF membrane was largely unaffected by high humidity and oxidation. However, a minor reduction was noted after the thermal cycling, again possibly linked to alterations in the boron nitride crystal structure (see Supplementary Fig. 16c for specific compressive stress data). Crucially, even after extended exposure to these demanding conditions, the SBF membrane remained intact and without damage when subjected to high-energy laser irradiation at 30 MW m^−2^ (see Supplementary Fig. 16d for specific laser threshold test data). These results demonstrate the material’s resilience and long-term functional reliability under demanding environmental stressors. These results collectively demonstrate the superior durability and long-term functional reliability of the SiO_2_@BN core-shell membrane, highlighting its promising potential for advanced high-energy laser protection applications.

In conclusion, we have successfully developed a core–shell nanofiber membrane composed of a highly reflective SiO_2_ shell and a thermally conductive BN core via multichannel electrospinning and carefully controlled calcination processes. This innovative architecture effectively addresses the persistent challenge of simultaneously achieving high optical reflectivity and efficient thermal management for high-energy laser protection. Experimental results demonstrate the membrane’s exceptional optical reflectivity (≈100% at 1064 nm), robust anisotropic heat conduction, and outstanding structural stability under continuous laser irradiation up to 36 kW cm⁻². Furthermore, mechanical evaluations indicate superior resilience and thermal stability, maintaining integrity even under extreme stress conditions.

Compared to BN aerogels with poor mechanical stability and ZrO_2_-based materials limited by isotropic thermal conduction, our SiO_2_@BN core–shell membranes achieve a unique combination of near-perfect reflectivity (>99.5%), high thermal anisotropy, and mechanical robustness—withstanding 95% compressive strain with full recovery. This design effectively addresses the challenge of simultaneously managing optical, thermal, and mechanical demands in high-energy laser protection. The specific applicability of this principle to new compound pairs can be inferred based on the difference in thermal conductivity between the selected oxide and nitride, as well as the chemical compatibility between the materials. The laboratory-scale electrospinning approach possesses inherent scalability for larger-area production, as throughput could be further enhanced through established technical optimizations such as multi-needle arrays. Looking forward, scaling the present electrospinning approach to industrial production will require optimizing precursor costs and processing throughput, alongside performance validation under complex multi-physics environments that combine thermal, mechanical, and humidity stresses. Despite these challenges, our work establishes a scalable design paradigm that advances multifunctional protective materials for aerospace and defense, and provides valuable insights for the future development of ceramic nanofiber technologies.

## Methods

### Materials

Unless otherwise specified, the materials and chemicals used in this work were purchased from Sigma-Aldrich, Greagent or Macklin, and were directly used without further purification. For the preparation of core–shell structured nanofiber membranes (SBF) and silica nanofiber membranes (SF), tetraethyl orthosilicate (TEOS, purity 98%), anhydrous ethanol (EtOH, purity ≥99.7%), and hydrochloric acid (HCl, 36–38%) were procured from Sinopharm Chemical Reagent Co., Ltd. Boron oxide (B₂O₃, purity 98%, Macklin Biochemical Technology Co., Ltd.) and polyvinylpyrrolidone (PVP, average molecular weight *M*_w_ = 8000 g mol^−1^, Shandong Keyuan Biochemical Co., Ltd.) were employed in the fabrication of core–shell structured and boron nitride nanofiber (BF) membranes. Additionally, cetyltrimethylammonium bromide (CTAB, purity 99%, Macklin Biochemical Technology Co., Ltd.) and boric acid (purity ≥99.5%, Shanghai Lingfeng Chemical Reagent Co., Ltd.) were respectively utilized in the preparation of the shell-layer and transition-layer electrospinning solutions for the core–shell structured membranes. The sodium meta-aluminate required for preparing the core-layer spinning solution was synthesized via neutralization reaction between sodium aluminate and dilute hydrochloric acid under an ice-water bath. The resulting sodium meta-aluminate was stored at low temperatures and used within 48 h.

### Fabrication of SBF

The SBF was prepared via coaxial electrospinning followed by multi-stage atmospheric calcination. First, a TEOS precursor underwent preliminary hydrolysis (TEOS/ethanol/water = 1:2:0.05 mass ratio, 80 °C, 8 h). The shell-layer solution was prepared by mixing the pre-hydrolyzed TEOS with CTAB (10:3 mass ratio) and vacuum distilling at 40 °C. The transition-layer solution was formulated by blending a boric acid/ethanol dispersion with the pre-hydrolyzed TEOS (2:1 mass ratio). The core-layer solution consisted of B_2_O_3_ and PVP (7 g:3.5 g in 10 ml ethanol), adjusted to pH 3–4 with sodium metaaluminate to elevate its melting point.

Electrospinning was conducted using a coaxial triaxial needle at 21 kV, a 15 cm collection distance, under strictly controlled conditions (<15 °C, <30% relative humidity). The preliminary composite membrane was then calcined in a furnace. Initially, it was heated at 2 °C min^−1^ to 600 °C for 2 h under an NH_3_/O_2_ (2:1 volume ratio) atmosphere to facilitate NH_3_ diffusion and byproduct outgassing through the mesoporous SiO_2_ shell. Subsequently, the atmosphere was switched to N_2_, and the temperature was raised to 1000–1200 °C for 2 h to yield the final SBF, followed by gradual cooling.

### Fabrication of SF and BF

For SF preparation, the shell-layer TEOS solution was directly electrospun and subsequently calcined in air at 800–1000 °C for 6 h. For BF preparation, a homogenized solution of B_2_O_3_ and PVP (1:1 mass ratio) in ethanol was electrospun, pre-calcined at 600 °C in air for 3 h, and ultimately calcined at 1200 °C in an N_2_ atmosphere for 3 h.

### Structural and morphological characterizations

Microstructural morphologies were examined using a field-emission scanning electron microscope (FE-SEM, GeminiSEM 500, 5 kV) on Pt-coated, cryo-fractured specimens. Internal core-shell structures were observed using a transmission electron microscope (TEM, JEOL JEM-2100, 200 kV) on ultramicrotomed cross-sections (≈ 70 nm thick). Small-angle and wide-angle X-ray scattering (SAXS/WAXS) were performed at the SSRF BL16B1 beamline (0.1239 nm) and using a Xeuss 3.0 system (Cu- *K*^α^, 1.54189 Å), respectively. Crystalline phases were identified via X-ray diffraction (XRD, D/max-2550VB, Cu-*K*^α^). Chemical compositions and states were analyzed using Fourier-transform infrared spectroscopy (FTIR, Thermo Nicolet 6700) and X-ray photoelectron spectroscopy (XPS, Thermo Scientific K-Alpha, Al *K*^α^). Raman spectra were recorded using a LabRAM HR Evolution microscope (532 nm laser, 10 mW).

### Optical and high-energy laser protection measurements

Optical reflection spectra were collected utilizing a 4P3-CUSTOM integrating sphere coupled with a USB2000+ spectrometer (200–850 nm). Laser protection properties were evaluated at 19 °C using a DK-YSM 2000 fiber laser (1064/1080 nm, 3 mm spot size) at power levels of 518, 1040, 1550, and 2050 W, corresponding to power densities of 7.3, 14.7, 21.9, and 29.0 kW cm^−2^. Real-time thermal responses during irradiation were monitored utilizing a FOTRIC 626CH-L25 infrared thermal camera.

### Thermal and mechanical evaluations

Thermal diffusivity was measured from 50 °C to 1200 °C in inert conditions using a laser flash apparatus (NETZSCH LFA 457 MicroFlash). Linear coefficients of thermal expansion (CTE) were determined using a Linseis L75VD1600LT vertical dilatometer (50–1200 °C, 5 °C min^−1^). Mechanical properties were assessed using a CMT4204 universal testing machine. Standard compression and tensile tests were conducted at a strain rate of 1 mm min^−1^. High-temperature in-situ compression tests were performed in an Ar atmosphere at 600–1800 °C (0.5 mm min^−1^). Microscale deformation mechanisms were observed via in-situ tensile testing within an FE-SEM (Merlin, Zeiss).

### Thermal transfer simulation

Simulations were conducted utilizing the steady-state solid thermal transfer module in COMSOL Multiphysics. A 500 °C thermal source was applied for 300 s, adhering to Fourier’s law, with carefully defined thermal conductivity, specific heat capacity, and density parameters for the fiber membrane layers.

## Supplementary information

Supplementary Information

Description of Additional Supplementary File

Supplementary Movie 1

Supplementary Movie 2

Supplementary Movie 3

Supplementary Movie 4

Transparent Peer Review file

## Source data

Source data

## Data Availability

All data to support the findings of this study are present in the paper and its Supplementary Information. Source data are provided with this paper.
