# Supplementary material for: Anisotropic core–shell ceramic nanofibrous membrane with improved optical reflectivity and thermal insulation for high-energy laser protection
Source: Nat Commun. 2026 Jul 23;17:7112. doi: 10.1038/s41467-026-73159-0 (PMC13396369; doi:10.1038/s41467-026-73159-0)
Supplement: Supplementary file 1 — Supplementary Information [file 41467_2026_73159_MOESM1_ESM.pdf]

Supplementary Information:

Anisotropic core–shell ceramic nanofibrous membrane with improved optical reflectivity and thermal insulation for high-energy laser protection

Huihuang Ma[a,b], Yikun Liu[a,b], Jianfei Gao[c], Xia Yang[a], Luo Luo[a], Yunfeng Tang[d], Xiaodong Zhou[a,e] and Liangshun Zhang[f*]*

[a] Key Laboratory of Specially Functional Polymeric Materials and Related Technology (Ministry of Education), School of Chemical Engineering, East China University of Science and Technology, Shanghai, 200237, China

[b] Shanghai Key Laboratory of Multiphase Materials Chemical Engineering, School of Chemical Engineering, East China University of Science and Technology, Shanghai, 200237, China

[c] School of Chemisty and Chemical Engineering, Henan University of Technology, Zhengzhou, 450001, China

[d] Institute for Engineering and Technology (Shanghai), Xinxing Cathay International Group, Shanghai, 400799, China

[e] Shanghai Engineering Research Center of Hierarchical Nanomaterials, East China University of Science and Technology, Shanghai, 200237, China

[f] Shanghai Key Laboratory of Advanced Polymeric Materials, School of Materials Science and Engineering, East China University of Science and Technology, Shanghai 200237, China

S.I. Supplementary experimental details

Fabrication of core–shell structured nanofiber membranes (SBF).

Preparation of electrospinning solutions. To achieve appropriate viscosity for electrospinning, the concentrations of the spinning solutions were carefully maintained below a defined threshold. For spinning solutions containing tetraethyl orthosilicate (TEOS), preliminary hydrolysis was performed under controlled acidic conditions to yield silanol oligomers. Specifically, TEOS, ethanol, and water were mixed in a mass ratio of 1:2:0.05 with a small amount of hydrochloric acid (1 mol L^{-1}), followed by refluxing in a flask at 80 °C for 8 h. The resulting solution was subsequently refrigerated for at least 12 h prior to use.

Shell-layer spinning solution: A measured amount of the pre-hydrolyzed TEOS solution was uniformly blended with cetyltrimethylammonium bromide (CTAB) at a mass ratio of 10:3. The mixture was then subjected to vacuum distillation at 40 °C for 1 h to obtain the final shell-layer spinning solution.

Transition-layer spinning solution: Boric acid was dispersed in ethanol and vigorously stirred for at least 24 h. Subsequently, the boric acid dispersion was carefully combined with the pre-hydrolyzed TEOS solution at a mass ratio of 2:1, with continuous stirring during addition. This mixture underwent identical vacuum distillation conditions (40 °C, 1 h) to yield the transition-layer spinning solution.

Core-layer spinning solution: B_2O_3 and polyvinylpyrrolidone (PVP) were thoroughly mixed at the following composition: 7 g B_2O_3, 3.5 g PVP, and 10 mL ethanol. After homogenization for at least 12 h, a small quantity of sodium aluminate was introduced to adjust the pH to ≈ 3–4, thus yielding the optimized core-layer spinning solution.

Electrospinning process. The shell-layer, transition-layer, and core-layer spinning solutions were separately loaded into an electrospinning apparatus equipped with a coaxial triple-concentric needle. Key electrospinning parameters, including the distance between the needle and the collector, applied voltage, and electric field intensity, were finely adjusted according to the viscosity of each spinning solution. For instance, for a spinning solution viscosity of ≈ 500 cP, optimal electrospinning conditions included an applied voltage of 21 kV, needle-to-collector distance of 15 cm, and needle height of 10 cm. Electrospinning was

conducted under strictly controlled environmental conditions—temperature below 15 °C and relative humidity below 30%. Following a stable and continuous electrospinning process, a preliminary composite membrane was obtained.

Calcination process. The preliminary composite membrane obtained via electrospinning was carefully transferred into a high-temperature atmospheric furnace and secured between quartz sheets. After ensuring airtight sealing of the furnace chamber, vacuum conditions were first established, followed by introducing a mixture of ammonia and oxygen gases at a volume ratio of 2:1 until the chamber was completely filled. During the calcination process, the ammonia (NH_3) was allowed to diffuse through the mesoporous SiO_2 shell, while the gaseous byproducts (H_2O, CO_2, and NH_3) were directed out through the newly formed channels in the shell. Subsequently, the gas flow rate was reduced, and the outlet valve was opened. Heating commenced at a controlled ramp rate of 2 °C min^{-1} until reaching a target temperature of 600 °C, which was maintained for 2 h before allowing the membrane to cool naturally. The gas atmosphere was then switched to nitrogen, and heating was resumed at an increased rate of 4 °C min^{-1} until reaching 800 °C, after which the heating rate was lowered to 2 °C min^{-1} until the maximum temperature of 1000–1200 °C was reached. The membrane was then held at this peak temperature for an additional 2 h, followed by gradual cooling at a controlled rate of 2 °C min^{-1} down to 600 °C, and finally allowed to cool naturally to room temperature. This meticulously controlled thermal profile significantly minimized thermal stress-induced shrinkage, preserving the smoothness and structural integrity of the fiber surfaces. The resulting core–shell structured nanofiber membrane, designated SBF, represented the final product of this sophisticated fabrication process.

Fabrication of silica nanofiber membranes (SF).

The preparation of silica nanofiber membranes similarly comprises three distinct stages: formulation of the spinning solution, electrospinning, and subsequent calcination. The spinning solution is directly obtained by utilizing the pre-hydrolyzed TEOS reflux solution previously described in the shell-layer preparation of the core–shell fibers. This solution undergoes vacuum distillation for 1 h to yield a suitable silica nanofiber spinning solution. Electrospinning

parameters, including electric field intensity, temperature (below 15 °C), and humidity (below 30%), are carefully controlled to ensure stable spinning over 8–12 h, yielding an initial silica nanofiber membrane of the desired thickness. The resultant membrane is calcined in air at temperatures ranging from 800 °C to 1000 °C, maintained for 6 h, producing the final silica nanofiber membrane.

Fabrication of boron nitride nanofiber membranes (BF).

The fabrication of BN nanofiber membranes follows a similar procedure. B_2O_3 and polyvinylpyrrolidone (PVP) are homogeneously mixed at the following ratio: 7 g B_2O_3, 7 g PVP, and 10 mL ethanol. After thorough mixing for at least 12 h, the resulting solution is loaded into a 10 mL syringe. Electrospinning is carried out with optimized electrical parameters; due to polymer incorporation, temperature and humidity adjustments are not strictly necessary, thus enabling the formation of uniform initial BN nanofiber membranes. These membranes are pre-calcined at 600 °C in air for 3 h, followed by additional calcination at 1200 °C under a nitrogen atmosphere for another 3 h, ultimately yielding the final BN nanofiber membranes.

S.II. Supplementary instrumentation and measurements

SEM observation.

To investigate the microstructural characteristics of the SBF, as well as the SF and BF membranes, morphological analyses were conducted using a field-emission scanning electron microscope (FE-SEM; GeminiSEM 500, Carl Zeiss Microscopy GmbH, Germany). Prior to imaging, the samples underwent freeze-drying, followed by cryogenic fracturing in liquid nitrogen to expose their cross-sectional morphologies. The fractured surfaces were mounted onto specimen stubs and subsequently coated with a thin layer of platinum using a Quorum Q150TES ion sputter coater (Quorum Technologies Ltd., UK) to enhance conductivity and mitigate charging effects during electron beam exposure. The coated specimens were then examined under the GeminiSEM 500 at an accelerating voltage of 5 kV and a probe current of 100 pA.

TEM observation.

SBF were prepared for TEM by standard fixation, dehydration, resin embedding, and ultramicrotomy. Samples were fixed with 2.5% glutaraldehyde in 0.1 M phosphate buffer (pH 7.4) for 2 h at room temperature, post-fixed with 1% osmium tetroxide, then dehydrated through a graded ethanol series (30%–100%) and embedded in epoxy resin (Araldite), which was polymerized at 60 °C for 24 h. Ultrathin cross-sections (≈ 70 nm thick) were cut perpendicular to the fiber axis using an ultramicrotome (Leica EM UC7) equipped with a diamond knife (Diatome) and collected on carbon-coated copper grids (300 mesh). Transmission electron microscopy was performed on a JEOL JEM-2100 TEM operated at an accelerating voltage of 200 kV, and bright-field images were recorded using a Gatan CCD camera.

SAXS and WAXS measurements.

To investigate the hierarchical crystalline structure and nanoscale periodicity of the SBF, both small-angle X-ray scattering (SAXS) and wide-angle X-ray scattering (WAXS) measurements were performed, as shown in Figure 2 and Supplementary Figure 5. SAXS experiments were conducted using the BL16B1 beamline at the Shanghai Synchrotron Radiation Facility (SSRF). The beamline was configured with double-crystal monochromator (DCM) to provide a monochromatic X-ray beam at a wavelength of 0.1239 nm (10 keV). The beam size at the sample position was defined by a set of slits to 0.4 mm × 0.4 mm, and the beam flux was ≈ 1×10^{12} photons s^{-1}. Two-dimensional (2D) SAXS patterns were recorded using a Pilatus 2M detector (Dectris, Switzerland) with a resolution of 2048 × 2048 pixels and a pixel size of 172 μm × 172 μm.

During data acquisition, the sample was fixed onto a flat optical table, and the sample-to-detector distances were precisely set to 2003 mm for SAXS and 687.9 mm for WAXS, respectively. The exposure times for SAXS and WAXS frames were 15 s and 10 s per acquisition, respectively. The effective scattering vector (q) range for SAXS was 0.002–0.820 nm^{-1}, while the angular diffraction range (2θ) for WAXS extended from 0° to 50°. To eliminate background contributions, raw intensity profiles were corrected by subtracting

scattering from pure water and air under identical conditions.

Quantitative analysis of SAXS and WAXS patterns was performed in reciprocal space using the scattering vector (q) and azimuthal angle (2θ) as coordinates. SAXS enabled determination of inter-domain spacing and core–shell periodicity within the SBF membranes, whereas WAXS provided insight into the crystalline domain size, interlamellar ordering, and short-range intermolecular arrangements.

XRD measurements.

For crystalline phase analysis via X-ray diffraction (XRD), the SBF samples were first cut into small pieces and ground into a fine powder using an agate mortar and pestle with ethanol as a grinding medium. The resulting suspension was dried to remove the ethanol, and the dried powder was packed into a standard XRD sample holder and gently pressed to create a smooth, flat surface for measurement. XRD measurements were then performed on a D/max-2550VB diffractometer (Rigaku, Japan) with Cu- K^{α} radiation (λ = 0.15406 nm), operated at 40 kV and 100 mA. Diffraction patterns were collected over a 2θ range from 5° to 80° with a step size of 0.02° and a scan speed of 2° min^{-1}.

FTIR measurements.

SBF membranes were cut into small pieces and ground into a fine powder in ethanol using an agate mortar and pestle. The resulting suspension was dried to remove the ethanol, yielding a fine powder, which was then thoroughly blended with spectroscopic-grade KBr at ≈ a 1:100 sample-to-KBr mass ratio. The homogenized mixture (≈ 100 mg) was pressed into a translucent pellet (13 mm diameter, ≈ 1 mm thickness) by applying ≈ 8 tons of pressure for 2 min in a hydraulic pellet press. Fourier-transform infrared (FTIR) spectra were recorded in transmission mode on a Thermo Nicolet 6700 FTIR spectrometer (Thermo Fisher Scientific, USA) equipped with a KBr beam splitter and a DTGS detector. Spectra were collected over the mid-infrared range of 4000-400 cm^{-1} at a resolution of 4 cm^{-1}, with 32 scans co-added per sample spectrum for an improved signal-to-noise ratio. A background spectrum of a blank KBr pellet was measured under the same conditions and automatically subtracted from the sample spectra.

XPS measurements.

XPS analysis was carried out using a Thermo Scientific K-Alpha system (Thermo Fisher Scientific, USA) equipped with a monochromatic Al K^{α} X-ray source (hν = 1486.6 eV). The SBF membranes were cut into small pieces (on the order of a few millimeters) and mounted on standard specimen stubs with conductive carbon tape to ensure good electrical grounding. The mounted samples were introduced via the instrument's load-lock and transferred into the analysis chamber under high vacuum (base pressure ≈ 1×10^{-7} mbar) for surface analysis. X-rays were generated at an accelerating voltage of 15 kV with a filament emission current of 10 mA, and the X-ray spot size was set to 400 μm. Wide-range survey spectra (0–1200 eV binding energy) were acquired at a pass energy of 200 eV and an energy step size of 1.0 eV to identify the elemental composition. High-resolution scans of the principal core-level regions (e.g., Si 2*p*, B 1*s*, N 1*s*, O 1*s*) were then collected with a pass energy of 50 eV (0.1 eV step size) for detailed chemical state examination. The total acquisition time for high-resolution regions was typically 5 min per region. A dual-mode charge neutralization system (low-energy electron flood gun and Ar^{+} ion beam) was utilized throughout the measurements to compensate for surface charging of the insulating samples. All spectral acquisitions were controlled via Thermo Avantage software, and binding energies were referenced to the C 1s peak at 284.8 eV for calibration.

Optical reflection intensity measurements.

To assess the reflectance properties of the SBF, SF, and BF membranes under high-energy laser irradiation, measurements were conducted using a 4P3-CUSTOM integrating sphere (Thorlabs, USA) coupled with a USB2000+ spectrometer (Ocean Insight, USA). Prior to measurement, membrane samples were cut into 10 mm × 10 mm squares and affixed to the sample port of the integrating sphere using a matte black, non-reflective adhesive to minimize edge scattering. The integrating sphere, featuring a 100 mm internal diameter and modular port configuration, was selected for its high reflectance inner coating, ensuring uniform light distribution within the sphere. A high-energy laser source was directed at the sample through

the entrance port, and the diffusely reflected light was collected by the sphere and transmitted via an optical fiber to the spectrometer. The USB2000+ spectrometer, covering a wavelength range of 200–850 nm, was calibrated using a certified reflectance standard prior to measurements. All data acquisition and analysis were performed using Ocean View software (Ocean Insight, USA).

Raman measurements.

The SBF membranes were cut into small sections and mounted flat on clean glass microscope slides for Raman analysis. Raman spectra were recorded at room temperature using a LabRAM HR Evolution confocal Raman microscope (Horiba Scientific, Japan) equipped with a 532 nm laser as the excitation source. The laser power at the sample was set to 10 mW using a series of neutral density filters, and a 100× objective lens (NA = 0.9) was used to focus the laser beam and collect the scattered light. Prior to measurements, the spectrometer was calibrated using the 520.7 cm^{-1} Raman band of a silicon standard to ensure accurate wavenumber readings. Spectra were collected over a wavenumber range of 100-4000 cm^{-1}, with an integration time of 10 s per acquisition, a spectral resolution of ≈ 2 cm^{-1}, and a 600 grooves mm^{-1} grating. Each spectrum was accumulated over 3 acquisitions to improve the signal-to-noise ratio. Data acquisition and instrument control were carried out using Horiba LabSpec 6 spectroscopy software. To eliminate the influence of sample fluorescence and stray light, the spectral background was corrected via a polynomial baseline subtraction algorithm integrated within the LabSpec 6 software.

Infrared thermal imaging measurements.

Thermal imaging was performed to evaluate the laser-induced heating of SBF, SF, and BF membranes. A FOTRIC 626CH-L25 infrared camera (FOTRIC, China; 640 × 480 pixel resolution, maximum temperature range 2000 °C) was aligned perpendicularly to the sample surface to capture real-time thermal images during irradiation. Membrane samples (10 mm × 10 mm squares) were mounted on insulating substrates and irradiated with a DK-YSM 2000 fiber laser (λ = 1080 nm, ≈ 3 mm spot size) at power levels of 518, 1040, 1550, and 2050 W.

The camera's emissivity setting was adjusted to match the sample surface characteristics, and measurements were carried out under controlled ambient conditions (constant room temperature and minimal airflow). The sequence of infrared images recorded over time was processed using FOTRIC's AnalyzIR software to generate temperature–time curves for each membrane, providing a quantitative measure of their heat response under the laser input.

High-energy laser irradiation measurements.

To evaluate the thermal resistance of the SBF, SF, and BF nanofiber membranes under high-power laser irradiation, experiments were conducted using a DK-YSM 2000 continuous-wave fiber laser (Hunan Dake Laser Co., Ltd., China) operating at a wavelength of 1080 nm. Specimens were cut into 10 mm × 10 mm squares and affixed to a thermally insulating substrate to minimize heat conduction during irradiation. The samples were positioned 3 cm from the laser output, ensuring a spot diameter of ≈ 3 mm on the sample surface. Laser power levels were set to 518 , 1040 , 1550 , and 2050 W, corresponding to power densities of 7.3, 14.7, 21.9, and 29.0 kW cm^{-2}, respectively. Each irradiation was performed at an ambient temperature of 19 °C, with exposure durations controlled precisely to assess the thermal stability and ablation resistance of the materials. Post-irradiation, the samples were analyzed for morphological and structural changes using scanning electron microscopy (SEM) and other relevant characterization techniques.

Thermal conductivity measurements.

Thermal diffusivity measurements for the SBF and the SF and BF membranes were performed using a laser flash apparatus (NETZSCH LFA 457 MicroFlash, Germany). Membrane samples were cut into disc specimens of 10 mm diameter (thickness 1–4 mm) and their thickness measured at multiple points to ensure uniformity; each disc was then coated on both faces with a thin graphite layer to enhance laser absorption and infrared emissivity. Thermal diffusivity was recorded over a temperature range from 50 to 1200 °C in a vacuum or flowing nitrogen atmosphere (inert conditions) to prevent oxidation. In each test, a short laser pulse was applied to the front face of the sample, and the temperature rise on the opposite face

was monitored by an infrared detector, allowing the calculation of thermal diffusivity from the time-resolved heat propagation signal. The instrument was operated via NETZSCH Proteus software, which automatically calculated the thermal conductivity (κ) by combining the measured thermal diffusivity (α) with the specific heat capacity (Cp) and density (ρ) of the sample (using $\kappa = \alpha \cdot \mathrm{Cp} \cdot \rho$).

Thermal expansion coefficient measurements.

To determine the linear thermal expansion coefficients (CTEs) of SBF, SF, and BF membranes, measurements were conducted using a L75VD1600LT vertical dilatometer (Linseis, Germany) over a temperature range of 50–1200 °C. Samples were prepared by cutting the membranes into cylindrical specimens with diameters not exceeding 7 mm and lengths up to 50 mm, ensuring flat and parallel end faces to facilitate accurate contact with the pushrod and sample holder. Prior to measurement, the length of each specimen was measured at multiple points using a high-precision caliper, and the average value was recorded. Each specimen was then placed vertically between the alumina pushrod and the sample holder within the instrument's furnace chamber. The chamber was evacuated to a base pressure below 10^{-2} mbar and subsequently purged with high-purity nitrogen gas to establish an inert atmosphere, preventing oxidation during heating. A constant contact force of 0.3 N was applied to maintain consistent contact between the specimen and the pushrod throughout the measurement. The temperature was increased at a controlled rate of 5 °C min^{-1} from 50 to 1200 °C. The change in length (ΔL) of each specimen was recorded continuously, and the CTE was calculated using the formula: $\alpha = \Delta L \ / \ (L_0 \cdot \Delta T)$, where L_0 is the original length and ΔT is the change in temperature. All measurements were controlled and analyzed using the Linseis TA software suite.

Mechanical measurements.

To comprehensively evaluate the mechanical properties of the SBF, SF, and BF membranes, a series of mechanical tests—including compression, tensile, in situ tensile under scanning electron microscopy (SEM), and high-temperature in situ compression tests—were conducted.

Compression measurements: Compression tests were performed using a universal testing machine (CMT4204, Shenzhen Xinsansi Material Testing Co., Ltd., China) at a constant strain rate of 1 mm min^{-1}. Prior to testing, specimens were prepared by cutting the membranes into rectangular samples with dimensions of ≈ 10 mm × 10 mm. The initial thickness of each specimen was measured at multiple points using a digital micrometer to ensure uniformity. During testing, specimens were placed between two compression platens, and load was applied until the desired strain was achieved. Subsequently, the load was removed to allow full recovery, and the final thickness was measured to assess the elastic recovery behavior.

Tensile measurements: Tensile properties were evaluated on the same universal testing machine equipped with appropriate tensile grips. Specimens were prepared in a dog-bone shape according to ASTM D638 Type V standards, with a gauge length of 25 mm. The specimens were clamped securely to prevent slippage, and tests were conducted at a crosshead speed of 1 mm min^{-1}. An extensometer was used to measure strain during the test, and the stress–strain data were recorded for analysis.

In-situ tensile measurements under SEM: To observe the deformation mechanisms at the microscale, in situ tensile tests were conducted within a field-emission scanning electron microscope (FE-SEM, Merlin, Zeiss) using a micro-tensile stage compatible with SEM chambers. Specimens were prepared by cutting the membranes into narrow strips (≈ 5 mm width) and mounting them onto the micro-tensile stage. The stage was then placed inside the SEM chamber, and tensile load was applied incrementally. Real-time imaging was performed to capture the evolution of microstructural features such as crack initiation and propagation during deformation.

High-temperature in-situ compression measurements: High-temperature compression tests were carried out using a high-temperature mechanical testing system equipped with a resistance furnace capable of reaching temperatures up to 1800 °C (CMT4204, Shenzhen Xinsansi Material Testing Co., Ltd., China). Specimens were cylindrical, with dimensions of ϕ10 mm (diameter) × 15 mm (height), and were prepared by pressing the nanofiber membranes into the desired shape. Tests were conducted in an inert argon atmosphere to prevent oxidation, with temperatures ranging from 600 °C to 1800 °C. A constant strain rate of 0.5 mm min^{-1} was

applied, and the deformation behavior was monitored using a high-temperature camera system.

S.III. Supplementary note

Heat transfer simulation.

To simulate the heat transfer behavior of the fiber membrane, the Heat Transfer in Solids interface within COMSOL Multiphysics was employed. The simulation was based on Fourier's law of thermal conduction expressed as:

$$q = -d_z k \nabla T \tag{1}$$

where q represents the heat flux vector, k denotes the thermal conductivity, and ∇T is the temperature gradient. The thermal radiation effect is actually considered in the COMSOL model. Our COMSOL model incorporates the surface-to-ambient radiation as a boundary condition, defined by the Stefan-Boltzmann law with a surface emissivity tied to the temperature-dependent reflectivity of the material. By the combined influence of the Knudsen effect and low Rayleigh number in our confined pore structure, the convective effects are negligible at the microscale of individual fibers. It is noteworthy that convective cooling at the membrane level remains crucial for overall performance, which will be addressed in our future work on system-level applications.

Model setup and parameters

A three-dimensional geometric model of the fiber membrane was constructed, reflecting its actual dimensions. Two architectures were modeled: a pure SiO_2 nanofiber and a core–shell nanofiber with a high-conductivity BN core.

Material properties: Thermal conductivity, specific heat capacity, and density were defined based on experimental measurements or literature values. The thermal conductivity was assumed to be isotropic and constant over the temperature range considered.

Interfacial thermal resistance (ITR): The ITR between the BN core and the SiO_2 shell was not explicitly included. This simplification is justified as the thermal boundary resistance at the micrometer scale is negligible, given the small Kapitza length (≈ tens of nanometers) compared to the fiber dimensions. Heat conduction is thus dominated by the porous SiO_2 matrix and radiative transfer at elevated temperatures.

Boundary conditions, mesh, and solver settings

Boundary conditions: A fixed temperature boundary condition of 500 °C was applied to one surface of the membrane to simulate the heat source. All other surfaces were treated as thermally insulated. Our model also incorporated surface-to-ambient thermal radiation through the Stefan-Boltzmann law, with temperature-dependent surface emissivity. Convective effects were considered negligible at the microscale due to the Knudsen effect and low Rayleigh number in the confined pore structures.

Study configuration: A time-dependent study was conducted over a duration of 300 s to observe the transient thermal response.

Mesh and solver: A physics-controlled mesh with finer elements near the heat source was utilized. The time-dependent solver employed adaptive time stepping to ensure numerical stability.

Simulation results and thermal evolution analysis

The simulated thermal evolution reveals a fundamental difference between the two architectures.

Pure SiO_2 nanofiber: After 300 s of heating at 500 °C, both the pure SiO_2 nanofiber and the underlying substrate reached 500 °C. The thermal distribution remains relatively uniform due to silica's intrinsically low thermal conductivity, a behavior also governed by structural factors such as the fiber in-plane packing density and interlayer spacing.

Core–shell SiO_2@BN nanofiber: In contrast, with a high-conductivity core, heat rapidly diffused into the core during the first ≈ 150 s. The subsequent ≈ 150 s of heating remained dominated by conduction within the core. Thus, the core–shell architecture channels heat primarily in the radial direction and significantly impedes its axial propagation, ensuring more effective interception of the laser energy before it can reach the substrate.

S.IV. Supplementary results.

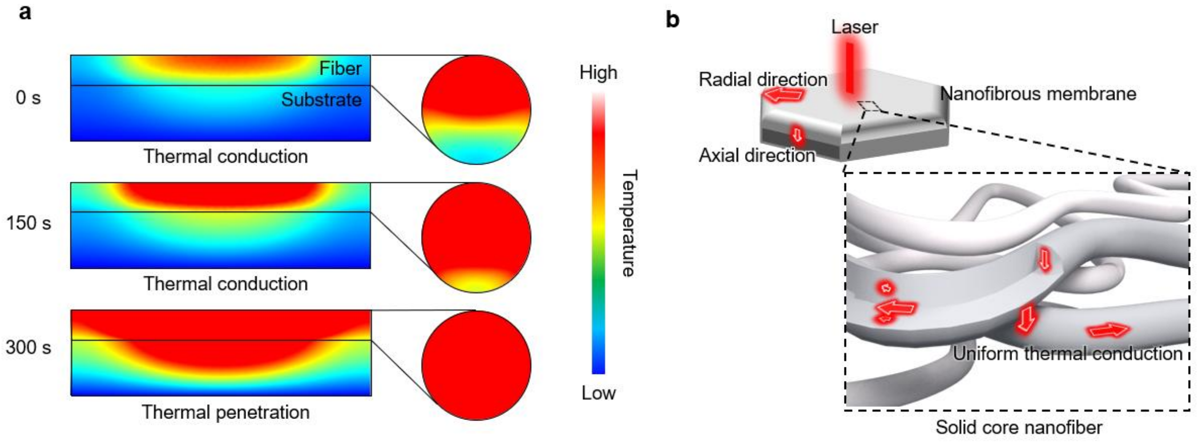

Supplementary Figure 1. Heat transfer simulation for solid nanofibers. a, Simulated thermal conduction behavior in solid nanofibers. **b**, Schematic illustration of the thermal transfer principle in solid nanofibers. After 300 s of heating at 500 °C, both the pure SiO2 nanofiber and the underlying substrate reached 500 °C. This homogeneous behavior is further governed by structural factors such as the fiber in-plane packing density and interlayer spacing.

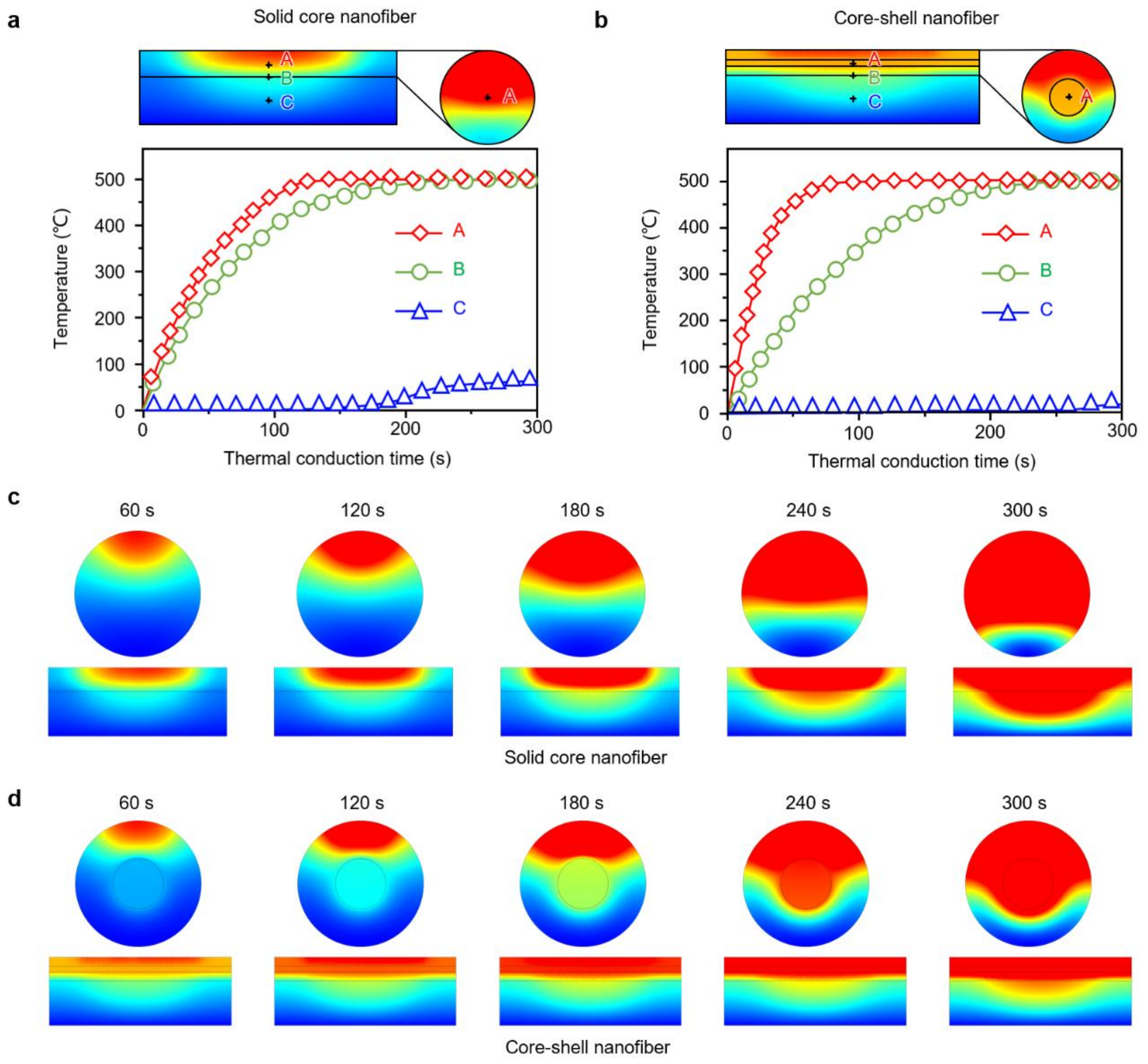

Supplementary Figure 2. Details of heat transfer simulation. a, b, Temperature evolution curves at points A, B, and C for solid fibers (SF) and core-shell fibers (CSF) as a function of heat-transfer duration. **c, d,** Cross-sectional and profile thermal distribution maps of SF and CSF captured every 60 s. The temperature evolution clearly indicates that the core-shell structure significantly slows the heat conduction toward the substrate surface, offering superior protective capability for the underlying materials. Supplemental heat distribution visuals further elucidate the directional heat conduction mechanism in CSF compared to SF.

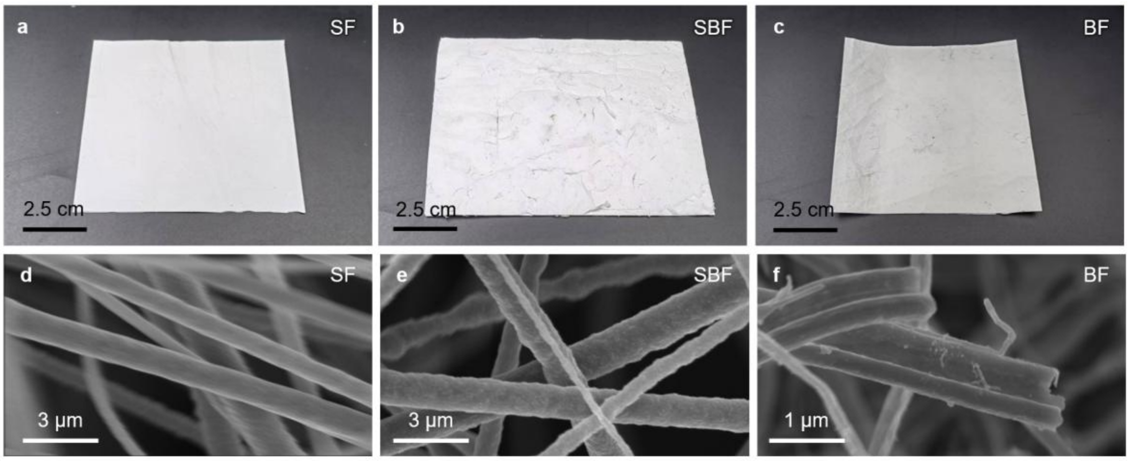

Supplementary Figure 3. Additional SEM images of SF, SBF, and BF samples. a–c, Macrostructural appearances of SF, SBF, and BF samples, respectively. **d–f**, Corresponding microscopic morphologies revealed by scanning electron microscopy (SEM).

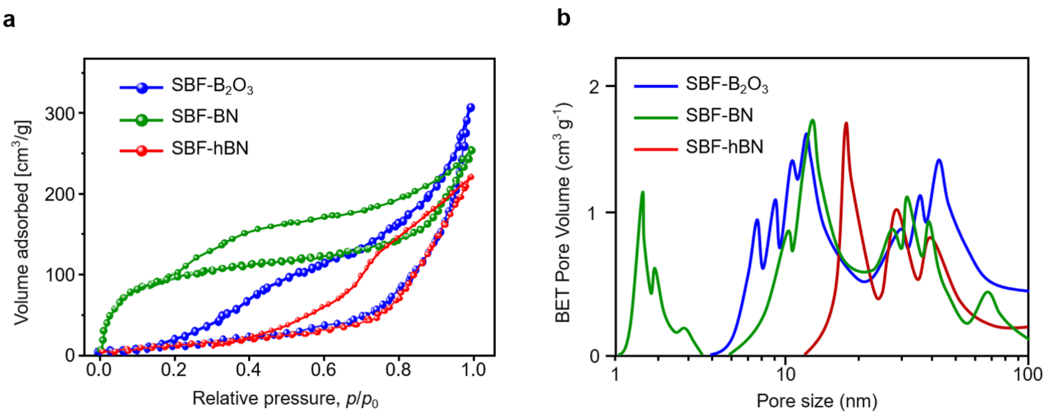

Supplementary Figure 4. The quantitative characterization of pore variations during the calculation process of SBF. a, N_2 adsorption-desorption isotherms and **b**, corresponding pore-size distribution curves of core-shell fibrous membranes at three key stages of fabrication process. The quantitative data establish a clear correlation between the template removal and mesopore formation, with subsequent densification eliminating the porosity as designed.

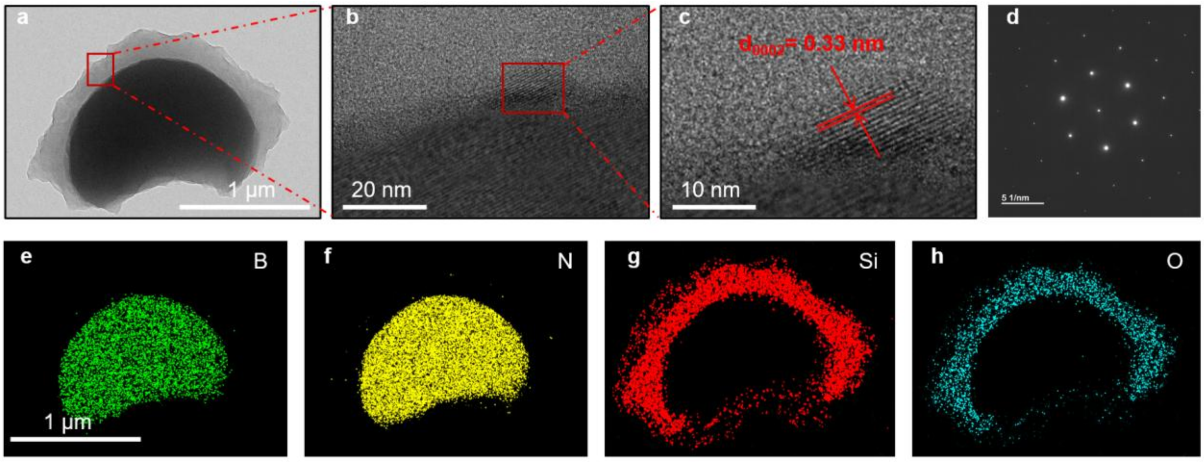

Supplementary Figure 5. HRTEM images and EDS of SF, SBF, and BF samples. a-c, High-resolution transmission electron microscopy images of the core-shell fiber at different magnifications. **d,** Corresponding selected-area electron diffraction pattern. **e–h,** EDS elemental maps of the core–shell nanofiber showing the spatial distribution of B, N, Si, and O. These results provide direct evidence of the well-defined BN-core/SiO_2-shell structure.

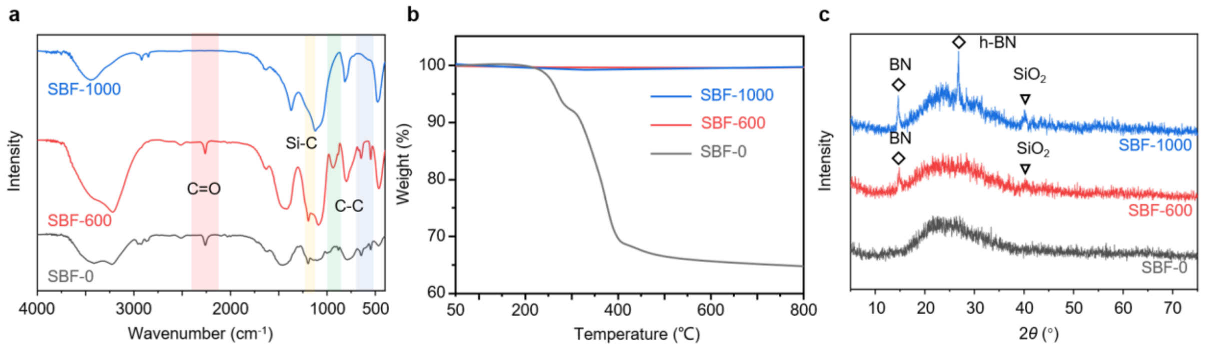

Supplementary Figure 6. Additional structural characterization of SBF at various calcination stages. a, Fourier-transform infrared spectroscopy (FTIR) spectra demonstrating variations in boron- and carbon-containing functional groups at different calcination stages. **b,** Thermogravimetric analysis (TGA) curves showing the complete removal of decomposable carbonaceous components starting from the second calcination stage. **c,** X-ray diffraction (XRD) profiles revealing the formation of BN crystals, amorphous silica changes, and minor crystalline SiO_2 phases through peak evolution.

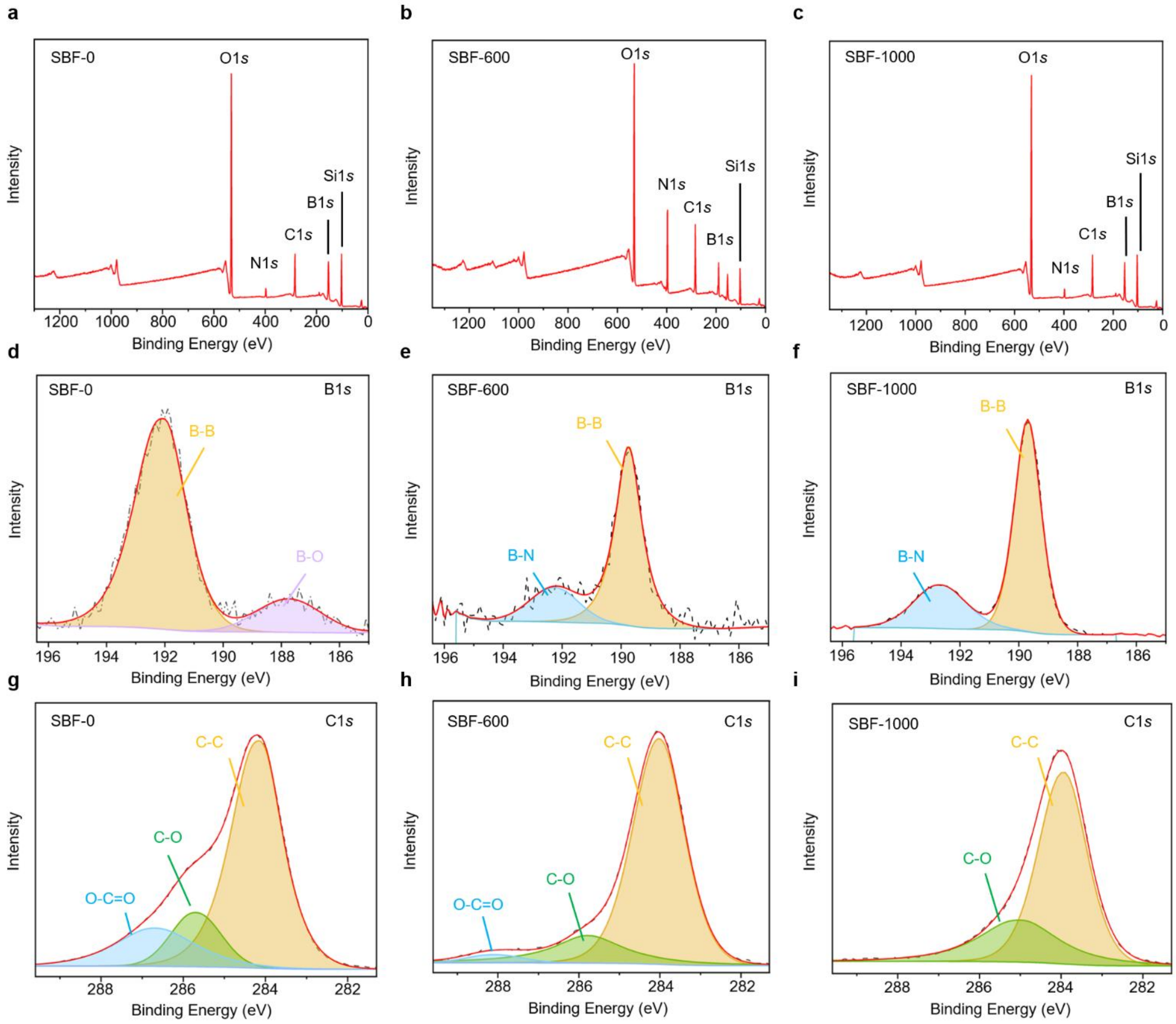

Supplementary Figure 7. X-ray photoelectron spectroscopy (XPS) results of SBF at various calcination stages. a–c, Evolution of characteristic XPS peaks during three calcination stages. **d–f,** Changes in boron-containing groups across these stages. **g–i,** Variations in carbon-based groups. XPS results conclusively support the transformation from B_2O_3 to BN, along with the gradual elimination of carbonaceous residues throughout the calcination process.

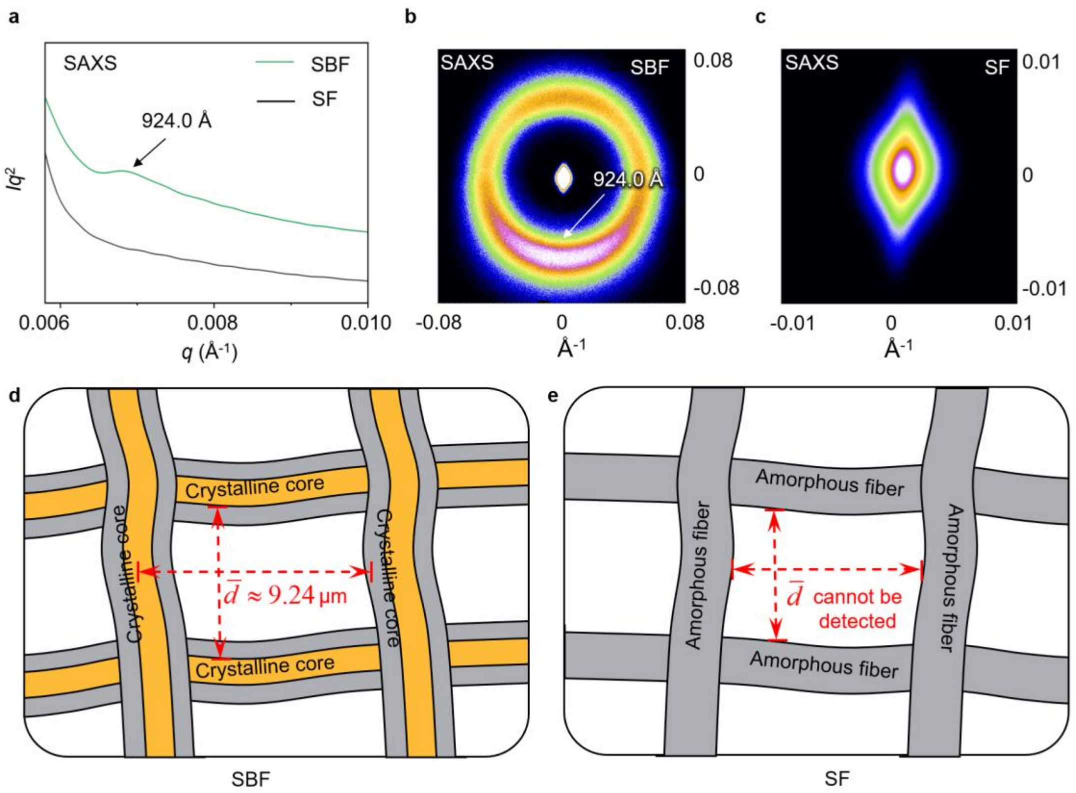

Supplementary Figure 8. Further interpretation of SAXS results. a, Comparative peaks in small-angle X-ray scattering (SAXS) profiles of SBF and SF within the in-plane direction. **b, c,** SAXS patterns for SBF and SF, respectively. **d, e,** Illustrations interpreting the peak occurrences. The presence of SAXS peaks in the low-angle region for SBF is primarily attributed to X-ray scattering induced by crystalline components in the fiber core. In contrast, SF, being amorphous, exhibits no detectable peaks at corresponding positions.

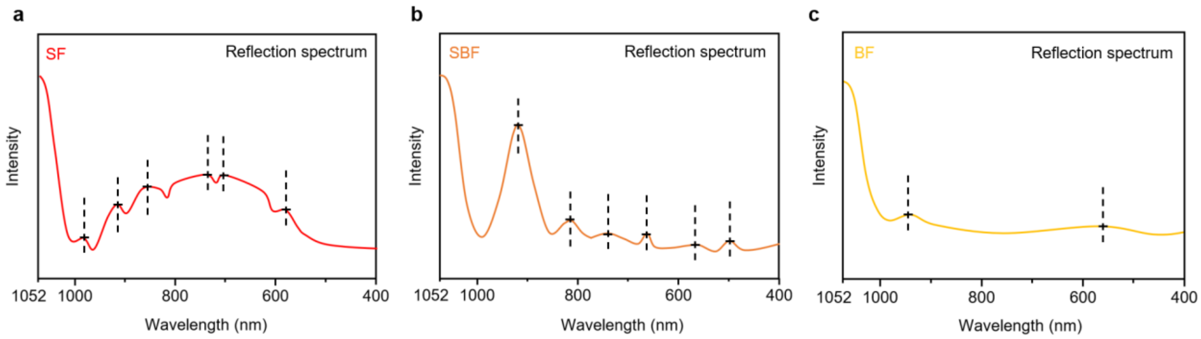

Supplementary Figure 9. Reflectance spectra of SF, SBF, and BF. Reflectance spectra of three fiber membranes demonstrate silica's pronounced capability to alter incident light wavelengths, whereas BN primarily provides strong reflectance without significant wavelength modulation.

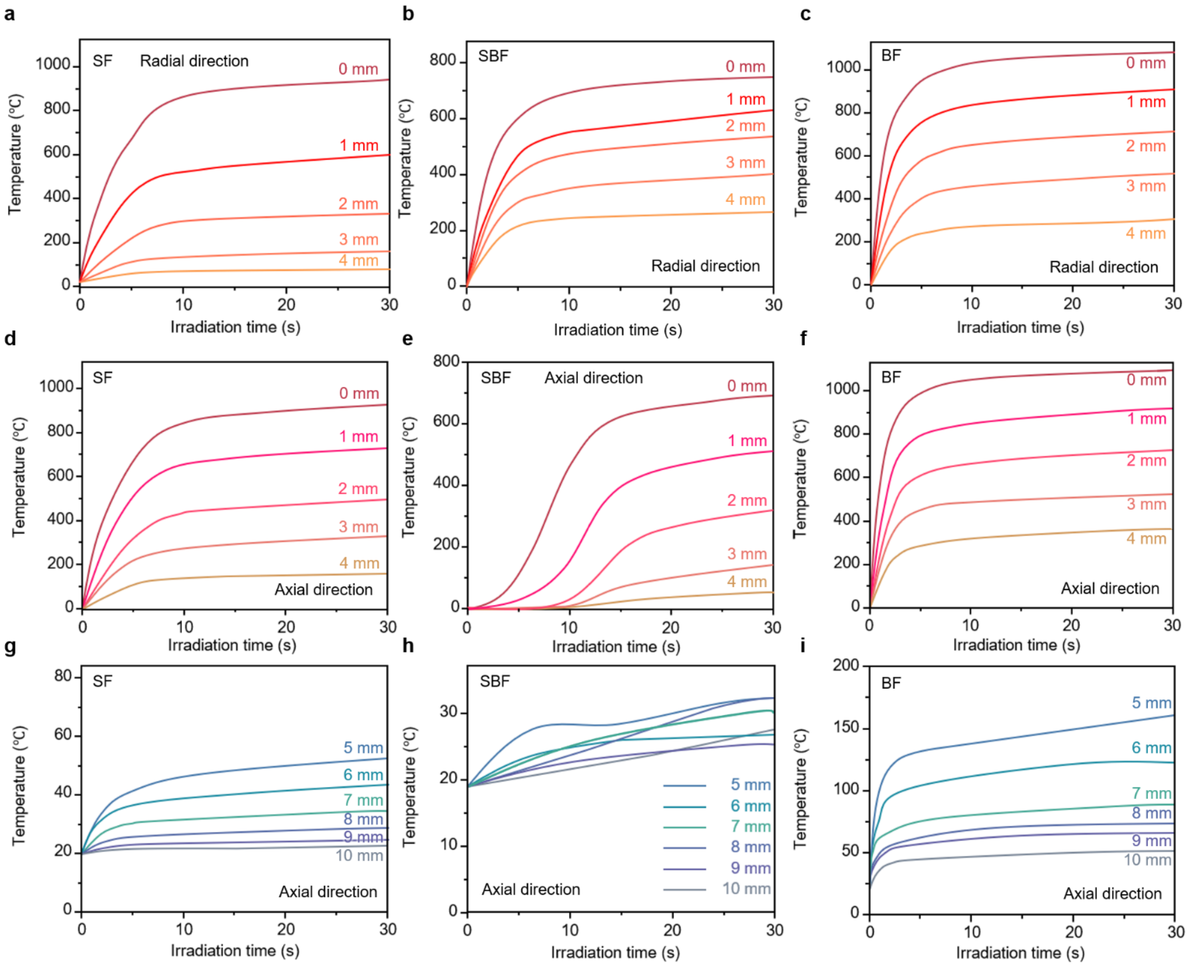

Supplementary Figure 10. Temperature evolution curves under high-energy laser irradiation for SF, SBF, and BF with varying thicknesses. a–c, Radial temperature variation with irradiation time at different measurement distances for SF, SBF, and BF. **d–i,** Axial temperature variations for these fibers. These datasets, serving as original source data for Figure 4d, further confirm that SBF displays anisotropic heat-transfer characteristics, providing superior thermal insulation performance in the axial direction compared to the other two samples.

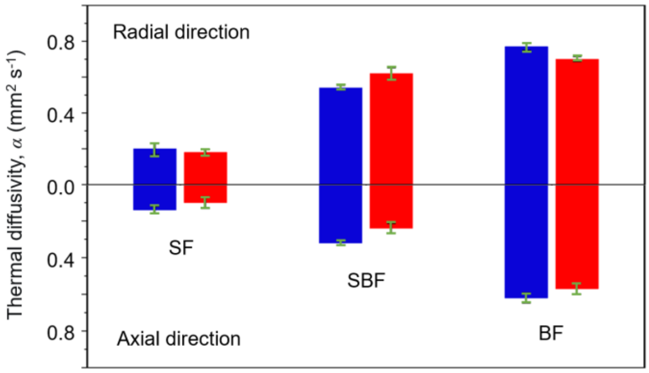

Supplementary Figure 11. Radial and axial thermal diffusivity of SF, SBF, and BF samples. The thermal diffusivity (α) are combined with specific heat capacity (Cp) and density (ρ) to derive the thermal conductivity values through using the relationship $\kappa = \alpha \cdot Cp \cdot \rho$. The excellent agreement between the calculated thermal conductivity and our previously reported data confirms the reliability of our thermal characterization. Data are presented as mean ± standard deviation, derived from n = 12 independent measurements for each condition.

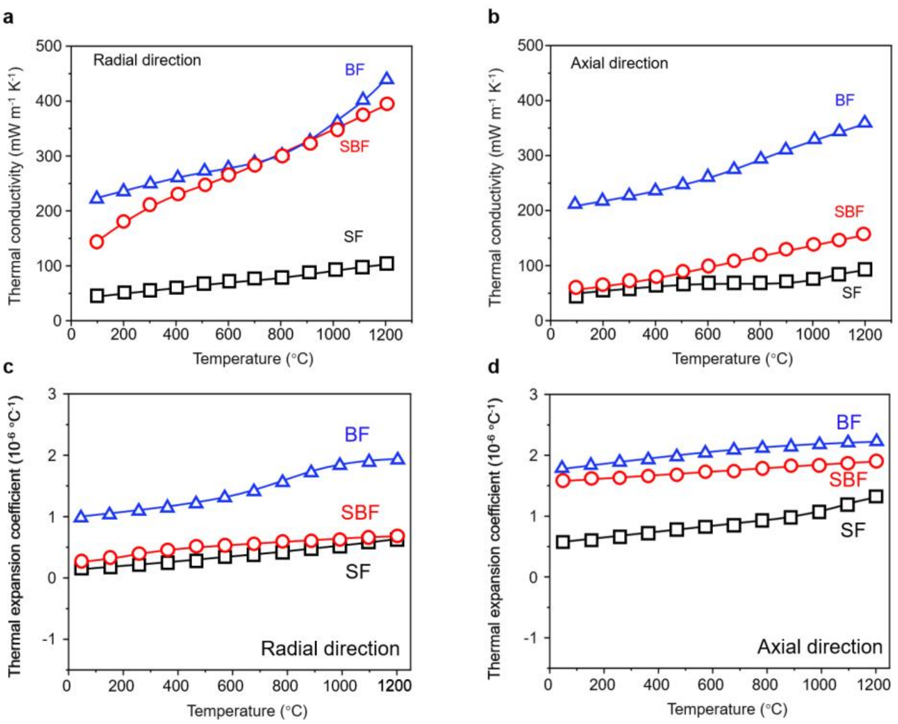

Supplementary Figure 12. Thermal conductivity and expansion coefficient variations of SF, SBF, and BF at different temperatures and orientations. a, b, Thermal conductivity variations as a function of temperature for SF, SBF, and BF in radial and axial orientations, respectively. The data indicate isotropic low thermal conductivity for SF, isotropic high conductivity for BF, and significantly anisotropic conductivity for SBF—demonstrating directional heat transfer capability crucial for substrate protection in laser-defense applications. c, d, Corresponding thermal expansion coefficient variations, revealing that SBF exhibits a low thermal expansion similar to SF in the radial direction, and a higher thermal expansion similar to BF axially, owing to its unique core-shell crystalline structure.

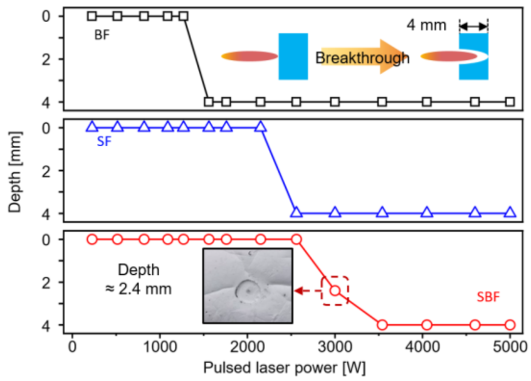

Supplementary Figure 13. Damage assessment of BF, SF and SBF samples under the pulsed laser irradiation at varying power levels. The inset is a schematic illustration of the ablation depth induced by high-energy laser irradiation and the surface morphology of SBFs after irradiation.

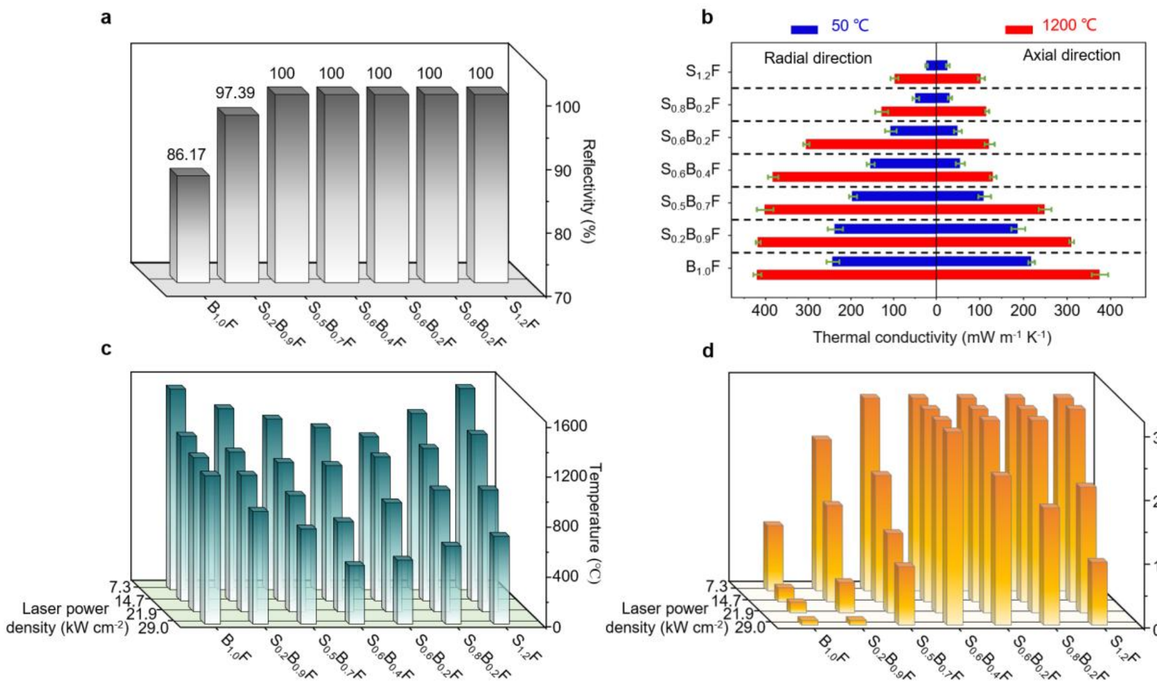

Supplementary Figure 14. a, Laser reflectivity, **b,** thermal conductivity measured along the axial versus radial directions, **c,** back-side temperature of a 2-mm-thick core-shell fibrous membranes and **d,** protection threshold of the fiber membrane under different laser output powers for silica (S)-boron nitride (B) core–shell fibers with varying core–shell ratios. Insets illustrate the two measurement orientations of thermal conductivity. Data in **b** are presented as mean ± standard deviation, derived from n = 12 independent measurements for each condition.

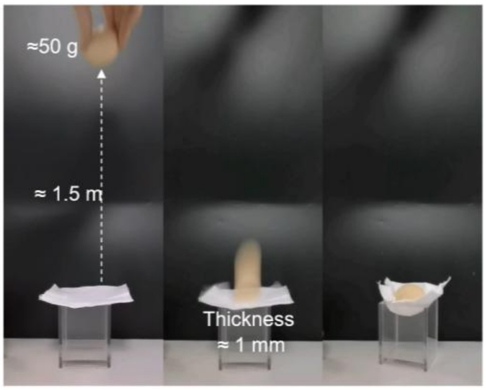

Supplementary Figure 15. Impact resistance test demonstrating superior cushioning capability of core–shell fiber membranes. The impact test can intuitively reflect that the SBF membrane has a certain buffer effect on the impact force.

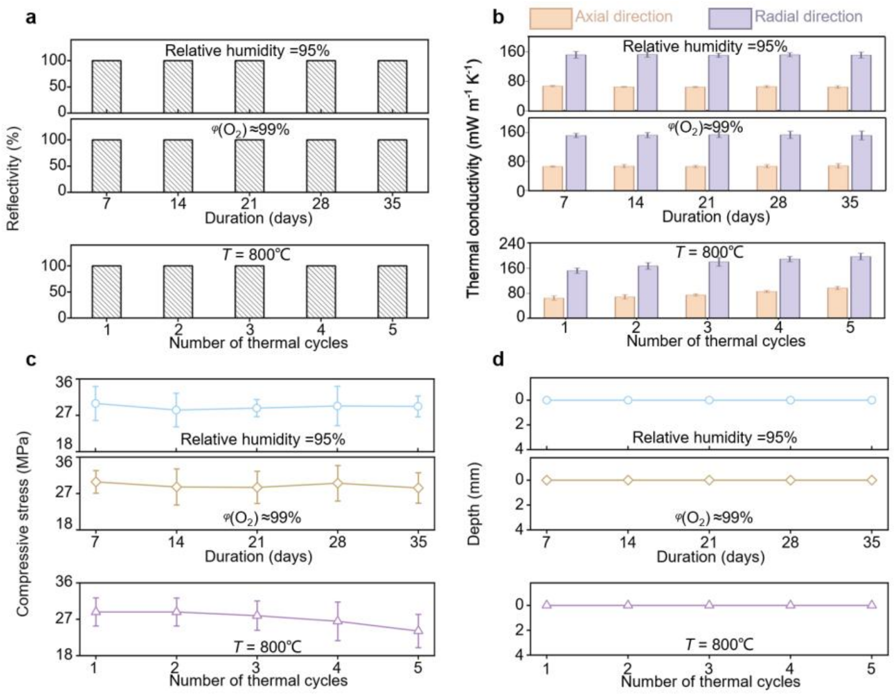

Supplementary Figure 16. Additional durability performance of SBF. Evolution of SBF sample's properties under high-humidity, high-oxygen, and thermal cycling conditions: **a,** reflectivity, **b,** axial and radial thermal conductivity, **c,** compressive stress, and **d,** laser-induced damage depth. Data in **b**, **c** are presented as mean ± standard deviation derived from n = 5 independent measurements.

Supplementary Table 1. Comparative data of protection threshold and performance of different laser protective materials. Corresponding to Figure 5b.

Materials	Laser protection threshold [$kW \cdot cm^{-2}$]	Laser protection time [s]	Thickness [mm]	References
GF/Ph	0.733	5	5	Ref. S1
BN aerogel	21	100	4	Ref. S2
$TaSi_2/ZrSi_2$/C-Ph	1	70	10	Ref. S3
GA/SiO_2	0.5	10	8	Ref. S4
LST	1	5	0.2	Ref. S5
ZrO_2/ZrB_2/SiC	6.3	10	2	Ref. S6
ZrC/CF/BPF	0.5	19	2.5	Ref. S7
SGS-Cu	2	48	2.5	Ref. S8
ZrB_2/Cu	2	180	3	Ref. S9
BPF	1	11	2.5	Ref. S10
GF/EP	0.5	5	2.5	Ref. S11
SBF	36	300	0.8	--

Supplementary references

S1. Zhang, J. *et al.* Laser Ablation Mechanism and Performance of Carbon Fiber-Reinforced Poly Aryl Ether Ketone (PAEK) Composites. *Polymers* **14**, 2676 (2022).

S2. Chai, Y., Li, G., Ji, X. & Zhang, X. Super-White Boron Nitride Aerogel-Enabled High-Energy Laser Irradiation Protection. *Adv. Funct. Mater.* **33**, 2304355 (2023).

S3. Xu, F. *et al.* Effect of TaSi2/ZrSi2 on ablation properties of carbon-phenolic composite irradiated by high-intensity continuous laser. *Ceram. Int.* **46**, 28443–28450 (2020).

S4. Li, W., Gao, L., Ma, Z. & Wang, F. Ablation behavior of graphite/SiO2 composite irradiated by high-intensity continuous laser. *J. Eur. Ceram. Soc.* **37**, 1331–1338 (2017).

S5. Zhu, J. *et al.* Ablation Behavior of Plasma-Sprayed $La_{1-x}Sr_xTiO_{3+\delta}$ Coating Irradiated by High-Intensity Continuous Laser. *ACS Appl. Mater. Interfaces* **9**, 35444–35452 (2017).

S6. Yang, M., Wang, T. & Wu, M. Ablation behavior of SiC whisker and ZrB2 particle-filled ZrO2 sol-gel composite coating under high-intensity continuous laser irradiation. *Ceram. Int.* **47**, 26327–26334 (2021).

S7. Ma, C. *et al.* Zirconium carbide-modified polymer-matrix composites with improved reflectivity under high-energy laser ablation. *Ceram. Int.* **45**, 17681–17687 (2019).

S8. Luo, C., Wang, S., Li, W., Zhang, L. & Pan, L. Mechanical properties of composite T-joints subjected to laser ablation. *Compos. Struct.* **294**, 115791 (2022).

S9. Yan, Z., Ma, Z., Liu, L., Zhu, S. & Gao, L. The ablation behavior of ZrB2/Cu composite irradiated by high-intensity continuous laser. *J. Eur. Ceram. Soc.* **34**, 2203–2209 (2014).

S10. Ma, C. *et al.* Ablation behavior of boron-modified phenolic resin irradiated by high-energy continuous-wave laser and its evolution of carbon structure. *Mater. Des.* **180**,

107954 (2019).

S11. Zhen, Y. *et al.* Ablation behavior and microwave absorption performance of metamaterials irradiated by high-energy continuous-wave laser. *Mater. Des.* **225**, 111502 (2023).
