# Supplementary material for: Anisotropic core–shell ceramic nanofibrous membrane with improved optical reflectivity and thermal insulation for high-energy laser protection
Source: Nat Commun. 2026 Jul 23;17:7112. doi: 10.1038/s41467-026-73159-0 (PMC13396369; doi:10.1038/s41467-026-73159-0)
Supplement: Supplementary file 2 — Description of Additional Supplementary File [file 41467_2026_73159_MOESM2_ESM.pdf]

The Description of Additional Supplementary Files

Supplementary Movie 1. Demonstration of the laser damage threshold of SBF membrane. SBF membrane placed atop several iron sheets and a silicon carbide (SiC) substrate withstands high-energy laser irradiation at 36 $kW \cdot cm^{-2}$ without damage. Upon removal of SBF membrane, the SiC substrate immediately shatters, and the iron sheets are penetrated, confirming that the protective threshold of SBF membrane surpasses 36 $kW \cdot cm^{-2}$, well above that of typical ceramic and metallic materials.

Supplementary Movie 2. Demonstration of sustained laser protection by SBF membrane. SBF membrane is exposed to a high-energy laser (36 $kW \cdot cm^{-2}$) for 5 min, measured with a quartz stopwatch. The experiment verifies the long-term stability and high-energy laser protective capability of SBF membrane.

Supplementary Movie 3. Demonstration of substrate protection and thermal insulation by SBF membrane. A model substrate covered with SBF membrane is irradiated with a highenergy laser. After 2 min of continuous exposure, the substrate remains undamaged, confirming the membrane's exceptional ability to prevent indirect heat-induced damage and effectively shield the underlying substrate from intense laser irradiation.

Supplementary Movie 4. Demonstration of mechanical integrity of SBF membrane at high temperature. SBF membrane supporting a suspended 500 g weight is continuously heated by a butane torch flame. The membrane does not fracture during heating and remains structurally intact after the flame is removed, highlighting its outstanding mechanical performance under extreme thermal conditions.
