# Supplementary material for: Anisotropic core–shell ceramic nanofibrous membrane with improved optical reflectivity and thermal insulation for high-energy laser protection
Source: Nat Commun. 2026 Jul 23;17:7112. doi: 10.1038/s41467-026-73159-0 (PMC13396369; doi:10.1038/s41467-026-73159-0)
Supplement: Supplementary file 7 — Transparent Peer Review file [file 41467_2026_73159_MOESM7_ESM.pdf]

nature portfolio

Peer Review File

Anisotropic core–shell ceramic nanofibrous membrane with improved optical reflectivity and thermal insulation for high-energy laser protection

Corresponding Author: Professor Xiaodong Zhou

This file contains all reviewer reports in order by version, followed by all author rebuttals in order by version.

Version 0:

Reviewer comments:

Reviewer #1

(Remarks to the Author)
This manuscript presents a novel core–shell nanofibrous membrane composed of a mesoporous SiO_2 shell and a crystalline BN core, developed via tri-axial electrospinning and multi-step calcination. The authors claim that the resulting material exhibits near-perfect optical reflectivity, anisotropic thermal conductivity, and superior mechanical robustness, offering a new paradigm for high-energy laser (HEL) protection materials. While the concept is innovative and the initial results are promising, there are several critical issues that must be addressed before the manuscript can be considered for publication.

Specific Concerns and Recommendations
Group A – Fundamental Concerns Requiring Additional Experiments and Clarification
1 Provide a systematic study with varying BN core diameters and volume fractions to clarify how structural parameters affect thermal and optical performance.
2 Perform directional thermal diffusivity measurements using laser flash analysis or the 3ω method to quantify thermal anisotropy.
3 The COMSOL simulations should include interfacial thermal resistance between the BN core and SiO_2 shell; if neglected, a justification must be provided.
4 Incorporate laser irradiation experiments under pulsed mode to better simulate realistic HEL conditions and assess material response to repeated heating cycles.
5 Extend thermal stability testing to high-humidity, oxidative, or thermocyclic environments to evaluate long-term durability.
6 Include dynamic simulations or empirical validation to confirm that Raman scattering contributes meaningfully to thermal dissipation as claimed.

Group B – Moderate Issues Requiring Minor Experiments, Discussion Expansion, or Formatting Adjustments
1 Expand the literature review to cover recent advancements in anisotropic thermal materials and their applications in laser protection.
2 Clarify the multi-order Raman scattering mechanism with more intuitive spectral decomposition or schematic support.
3 Improve image quality for SEM/IR figures and ensure scale bars are clearly labeled and consistent.
4 Move detailed COMSOL parameters and repeatability data to the supplementary section to improve manuscript clarity.
5 Provide more detailed instrumentation parameters for characterization techniques (e.g., XPS, SAXS, Raman).
6 Standardize citation formatting and correct inconsistencies in the reference list.

Reviewer #2

(Remarks to the Author)
This manuscript presents a SiO2@BN core–shell nanofibrous membrane that combines ultrahigh optical reflectivity with anisotropic thermal management for high-energy laser protection. The work is innovative in its material design, fabrication process, and multidimensional characterization. In particular, the integration of BN crystalline cores with SiO2 shells via multi-channel electrospinning and staged calcination is technically impressive. The experimental results convincingly demonstrate outstanding optical scattering, directional heat conduction, and mechanical strength, with extensive comparisons to existing materials. Overall, the study is original, has significant potential for applications, and fits well within

the scope of Nature Communications. Nevertheless, there are several important issues that need to be addressed before the manuscript can be considered for publication.
1. The thermal conductivity and stability of BN are strongly affected by crystallinity and defects. While XRD and XPS are provided, the absence of quantitative Raman analysis or high-resolution TEM defect characterization leaves the microstructural basis for the reported performance insufficiently supported.
2. The unique anisotropic thermal conduction is emphasized, but discussion of the interfacial thermal resistance between BN cores and SiO2 shells is limited. Currently, only simulations and macroscopic experimental data are available, lacking direct characterization or a more in-depth theoretical explanation.
3. The COMSOL simulations only consider heat conduction, ignoring the inevitable radiation and convection effects under laser irradiation.
4. The study focuses on single or short-duration laser exposures. Considering practical scenarios, cyclic laser shock tests or long-term thermal shock stability experiments are necessary to evaluate durability under realistic service conditions.
5. The conclusion that radial conduction is dominated by BN cores is not fully substantiated. Additional evidence is needed to demonstrate that heat preferentially flows through the BN core rather than the SiO2 shell.
6. The analysis of tensile and compressive behavior is largely descriptive based on stress-strain curves. A more detailed mechanistic explanation of the cooperative role of core and shell during failure would strengthen the discussion.
7. It is recommended that the conclusion section more clearly highlight the advantages of this material over existing BN aerogels and ZrO2-based materials in practical protection applications. The paper should also acknowledge the potential limitations of this process in terms of cost, scalable production, or complex environments.
8. In Figure 5b, several benchmark materials are drawn from older reports. More recent advances in nanoporous ceramics and metal-based composite coatings should be incorporated to provide a fairer and more convincing comparison.
9. The Supplementary Information contains essential experimental details, yet these are not sufficiently cross-referenced in the main text. Clearer integration (e.g., “see Supplementary Fig. S3 for FTIR evolution”) would improve readability and transparency.

Reviewer #3

(Remarks to the Author)
The author developed a three-layer co-spun electrospinning method and successfully prepared core-shell structured nanofibers. The structural design of SiO2-BN nanofibers is novel, which provides a new idea for the design of thermal protection materials.

1. From tri-layer core-shell structure to SiO2@BN core-shell nanofibers, what is the diameter contraction ratio of the fiber? How to control the discharge of gas during PVP decomposition in the core is very critical. Especially in the process of B2O3 to BN, how to control NH3 to enter the interior through the surface pore without reacting with the surface?

2. In anisotropic SiO2@BN core-shell nanofibrous membrane, how to ensure that the distribution of BN in a single fiber is continuous? If there is an interruption, it will produce a thermal resistance effect.

3. The fiber membrane is composed of a combination of single fibers interwoven longitudinally and transversely. The laser action and the surface will produce strong diffuse reflection and fiber surface reflection, which exists in both SF and SBF fiber membranes. This is related to the surface state of the fiber membrane and the fiber overlapping structure, and seems to have no direct relationship with BN.

4. In Figure6b, does the length of the fiber change under compression? SiO2@BN is an inorganic fiber and has a large brittleness. Why does a single fiber deform greatly? In Figure6f, how to obtain mechanical and thermal performance results at 1500℃ and 1800℃?

5. Please explain why SBF has a higher ability to withstand high power laser than SF and BF? Why does SBF not show breakdown phenomenon when surface melting occurs?

Reviewer #4

(Remarks to the Author)
The manuscript reports the preparation of a SiO2@BN core–shell nanofibrous membrane by ingenious tri-layer electrospinning and controlled calcination, followed by achieving ultrahigh optical reflectivity and superior thermal insulation for high-energy laser protection. This work represents a significant advance in high-temperature thermal protection especially for flexible ceramic continuous nanofibers. The study is intriguing and is expected to be valuable for applications that extend well beyond high-energy laser protection. It is clearly articulated and logically structured, and the characterization techniques employed are state-of-the-art. I therefore anticipate broad reader interest and recommend publication on Nature Communications, provided the following issues are addressed.

1. The recent work by Pan et al. (Science Bulletin, 2025, 70, 722-732) on BN/SiO2 core–shell nanofiber membranes has not been cited or discussed. The novelty of the present SiO2@BN architecture relative to the previously reported BN/SiO2 arrangement must be explicitly clarified, preferably in the introduction, so that the incremental advance is unambiguously established.
2. The core–shell morphology remains insufficiently substantiated. Figure S2 mapping result shows no nitrogen, while the boron also distributed in the shell instead. Additional high-resolution line scans or phase maps are therefore required to confirm that the core is indeed BN and that the shell is SiO2.

3. The assertion that CTAB generates a nanoporous shell is currently based on TEM images alone; nitrogen absorption isotherms should be acquired to quantify porosity and to validate the templating mechanism.
4. The axial/radial terminology appears to have been reversed. The abstract states that heat is "rapidly dissipated radially while insulated axially"; however, for a fiber geometry the converse is expected. This inconsistency should be rectified throughout the text.
5. The thermal simulation was run for 300 s, whereas the actual laser irradiation experiment lasted only 30 s. The rationale for this discrepancy must be provided to avoid a questionable model.
6. The possibility of elemental boron or boron-rich non-crystalline phases forming within the shell has not been excluded. More results are required to verify the exclusive presence of h-BN and to assess the thermal stability contributions of any amorphous boron in the shell.
7. The compression micrographs schematically in Figure 6b suggest a marked diameter reduction, which is atypical for ceramic fibers in my opinion.
8. This manuscript would benefit from a brief demonstration of how the SiO2@BN core–shell ratio can be tuned and how such tunability affects laser damage thresholds and thermal insulation performance.

Version 1:

Reviewer comments:

Reviewer #1

(Remarks to the Author)
The authors have substantially revised their manuscript entitled “Anisotropic SiO_2@BN Core–Shell Nanofibrous Membrane with Ultrahigh Optical Reflectivity and Superior Thermal Insulation for High-Energy Laser Protection.”
All major concerns raised in the first review—regarding structural parameter optimization, thermal-diffusivity measurements, interfacial thermal resistance justification, pulsed- and cyclic-laser tests, environmental durability, and Raman-related thermal dissipation—have been addressed through additional experiments and clarifications. The revised manuscript now presents a comprehensive and coherent dataset that convincingly supports the claimed multifunctional performance of the SiO_2@BN core–shell system. Figures, image quality, and citation consistency have also been notably improved.
Assessment of Revisions
The newly added results, including laser-flash thermal diffusivity measurements, parametric studies of core–shell ratios, and cyclic irradiation tests, provide direct and quantitative support for the anisotropic thermal management concept.
The justification for neglecting interfacial thermal resistance through Kapitza-length estimation is physically reasonable, and the extended environmental-stability tests further validate material reliability. The overall narrative—from structural design to optical scattering, anisotropic heat conduction, mechanical integrity, and laser-shielding performance—is now logically continuous and experimentally substantiated.
The manuscript is significantly strengthened and, in my view, meets the scientific and technical standards of Nature Communications.
Only several clarifications or minor refinements would further enhance completeness.
Minor Comments and Suggestions
1. The discussion of the Raman scattering contribution remains qualitative. A simple energy-balance estimate or reference-based evaluation of photon-to-phonon conversion efficiency would make this mechanism more convincing.
2. Please add a brief comment on whether the “radial-conduction/axial-insulation” principle could be extended to other oxide/nitride systems (e.g., SiO_2@AlN, ZrO_2@BN).
3. Some newly added figures (R4–R5 and related SI data) could be more clearly linked in the main text by transitional sentences specifying which property each test validates.
4. Consider including one sentence quantifying achievable membrane area or electrospinning throughput, to contextualize potential for large-scale fabrication.

Reviewer #4

(Remarks to the Author)
The revised manuscript has addressed most of my concerns and demonstrates further improvement in quality. That said, while the authors clarified in their response that “the terms 'radial' and 'axial' in our manuscript are defined with respect to the macroscopic membrane geometry, not individual fibers,” Figure 1c still appears to use the orientation of a single fiber as reference. Moreover, since the key innovation of this work is fundamentally based on individual fiber design, I strongly recommend that the take full attentions to this point throughout the manuscript. There is no inherent contradiction or complexity between thermal conduction at the single-fiber level and that of the fibrous membrane as a whole. However, I believe the authors have not adequately addressed this relationship. This is particularly evident in the abstract, where they state: “By virtue of dual-channel electrospinning and precise calcination, the membrane structure achieves ultrahigh reflectivity (~100% at 1064 nm) while enabling anisotropic thermal conduction—rapidly dissipating heat radially across the membrane plane while insulating axially through its thickness.” Such a description deviates from conventional fiber science and is likely to cause significant confusion among readers especially in fiber area. Alternatively, the membrane should at least be clearly labeled with 'radial' and 'axial'in the main text figure to clearly distinguish the difference.

It is unclear whether the EDS analysis (Figure S5) was performed after the "preliminary calcination" or the final "high-temperature calcination" step. This distinction is critical for interpreting the results: If it is after the preliminary calcination, one might expect to observe a boron gradient or a diffuse interface due to the transitional state of the core-shell formation. The

presence of a sharp interface, as shown, would therefore be unexpected and require explanation. If it is after the high-temperature calcination, a significant discrepancy arises between the core-shell ratio evident in the EDS mapping (Figure S5) and that shown in the TEM image of Figure 2e. This inconsistency in the observed core-to-shell dimensions needs to be clarified.

Besides,a noticeable inconsistency exists in the reported fiber diameters between the SEM and TEM images. The SEM images in Figures 2f and 2g suggest fiber diameters on the order of several hundred nanometers. In contrast, the TEM images in Figures 2c and 2e, based on their scale bars, indicate diameters in the micrometer range. The authors should verify the accuracy of all scale bars and provide a coherent explanation for this apparent discrepancy.

I believe the authors have adequately addressed Reviewer 3's concerns.

Reviewer #5

(Remarks to the Author)

Version 2:

Reviewer comments:

Reviewer #1

(Remarks to the Author)
The revisions to the manuscript fully address my concerns and meet my expectations. I am pleased to recommend its acceptance in the current form.

Reviewer #4

(Remarks to the Author)
My concerns have been addressed in the revisions.

Open Access This Peer Review File is licensed under a Creative Commons Attribution 4.0 International License, which permits use, sharing, adaptation, distribution and reproduction in any medium or format, as long as you give appropriate credit to the original author(s) and the source, provide a link to the Creative Commons license, and indicate if changes were made.
In cases where reviewers are anonymous, credit should be given to 'Anonymous Referee' and the source.
The images or other third party material in this Peer Review File are included in the article’s Creative Commons license, unless indicated otherwise in a credit line to the material. If material is not included in the article’s Creative Commons license and your intended use is not permitted by statutory regulation or exceeds the permitted use, you will need to obtain permission directly from the copyright holder.
To view a copy of this license, visit https://creativecommons.org/licenses/by/4.0/

Responses to the Reviewers' comments

We thank the Reviewers for their careful review of our manuscript and for providing constructive comments and suggestions to improve the quality of the manuscript. Our point-by-point responses to these comments are given below. The corresponding contents were incorporated into the manuscript and highlighted in blue.

Reviewer: #1

Comments: *This manuscript presents a novel core–shell nanofibrous membrane composed of a mesoporous SiO_2 shell and a crystalline BN core, developed via tri-axial electrospinning and multi-step calcination. The authors claim that the resulting material exhibits near-perfect optical reflectivity, anisotropic thermal conductivity, and superior mechanical robustness, offering a new paradigm for high-energy laser (HEL) protection materials. While* ***the concept is innovative and the initial results are promising****, there are several critical issues that must be addressed before the manuscript can be considered for publication.*

Response: We appreciate the Reviewer's positive comments on our manuscript, such as "*the concept is innovative and the initial results are promising*". However, the Reviewer has several concerns about the manuscript. Our addresses to these concerns are as follows:

Specific Concerns and Recommendations

Group A – Fundamental Concerns Requiring Additional Experiments and Clarification

Comment #1.1: *Provide a systematic study with varying BN core diameters and volume fractions to clarify how structural parameters affect thermal and optical performance.*

Response/Action: We thank the Reviewer for raising the important point about the effect of structural parameters on thermal and optical performance. In response to Reviewer's comment, we have conducted an additional study how the BN core diameter and SiO_2 shell thickness affect the material performance. This was achieved by controlling the core–shell ratio through the feed rates of the inner BN (abbreviated as B) and outer SiO_2 (S) precursor solutions during

electrospinning. A higher relative feed rate for the SiO_2 precursor results in a larger shell thickness relative to the core diameter.

The key results are summarized in **Figure R1**, which plots the hemispherical reflectivity, thermal conductivity, and laser damage threshold against the S:B precursor feed-rate ratio. The data reveal that the hemispherical reflectivity exhibits a strong dependence on shell thickness, increasing progressively from ~85% to nearly 100% as the SiO_2 proportion increases. The reflectivity reaches a maximum and plateaus at a S:B precursor ratio of 0.5:0.7, suggesting that the SiO_2 shell forms a continuous, highly reflective layer at this optimal ratio. The measurements of directional thermal conductivity clearly demonstrate that the thermal anisotropy is most pronounced at a S:B ratio of 0.6:0.4. At this specific ratio, the radial thermal conductivity reaches its maximum while maintaining low axial conductivity, achieving the most favorable conditions for in-plane heat spreading combined with effective through-plane insulation. The laser-induced damage threshold reveals an interesting transition in protection mechanism. At lower power densities (< 20 kW/cm²), samples with thicker SiO_2 shells (S:B = 0.5:0.7) exhibit superior performance due to enhanced reflectivity. However, under high-power conditions (> 30 kW/cm²), the optimal S:B ratio of 0.6:0.4 provides the highest damage tolerance, as the balanced core-shell structure enables simultaneous reflective protection and efficient heat dissipation. **These results confirm that the samples in our original manuscript (prepared at S:B = 0.6:0.4) represents the optimal composition that balances multiple performance requirements for practical high-power laser protection applications.**

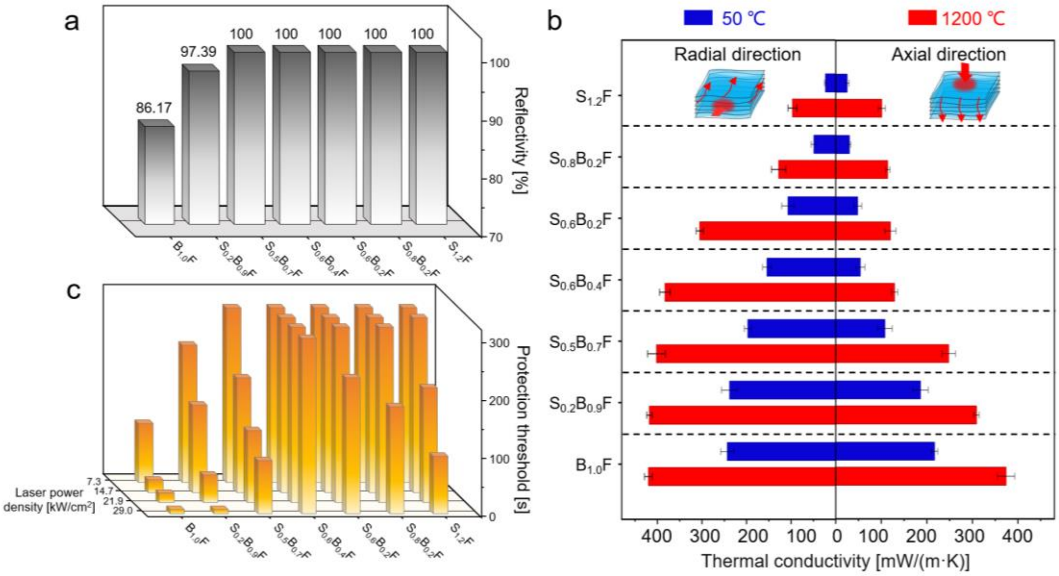

Figure R1. (a) Laser reflectivity, (b) thermal conductivity measured along the axial and radial directions, and (c) protection threshold of the fiber membrane under different laser output powers for core–shell fibers with various of outer SiO_2 (S) to inner BN (B) precursors. Insets illustrate the two measurement orientations of thermal conductivity.

The above discussion about the effect of structural parameters on thermal and optical performance was incorporated into the revised manuscript. **(Page 27, Lines 5-22; Page 28, Lines 1-8; Page S28, Figure S14 of the SI)**

Comment #1.2: *Perform directional thermal diffusivity measurements using laser flash analysis or the 3ω method to quantify thermal anisotropy.*

Response/Action: We thank the Reviewer's suggestion about the directional thermal diffusivity measurements for the thermal anisotropy. In response, we have performed the laser flash analysis (LFA) to determine the thermal diffusivity (α) in both radial and axial directions. As shown in Figure R2, the radial thermal diffusivity of SBF increases at high temperature, while those of SF and BF decrease, demonstrating the superior radial heat dissipation capability of SBF under thermal loading. Additionally, the axial thermal diffusivity values are lower than the radial ones across all samples, originating from the interlayer porosity for serious hinders of in-plane heat transfer.

The thermal diffusivity (α) is combined with specific heat capacity (C_p) and density (ρ) to derive the thermal conductivity values through using the relationship $\kappa = \alpha \cdot C_p \cdot \rho$, which are presented in Figure 4e of our revised manuscript. The excellent agreement between the calculated thermal conductivity and our previously reported data confirms the reliability of our thermal characterization.

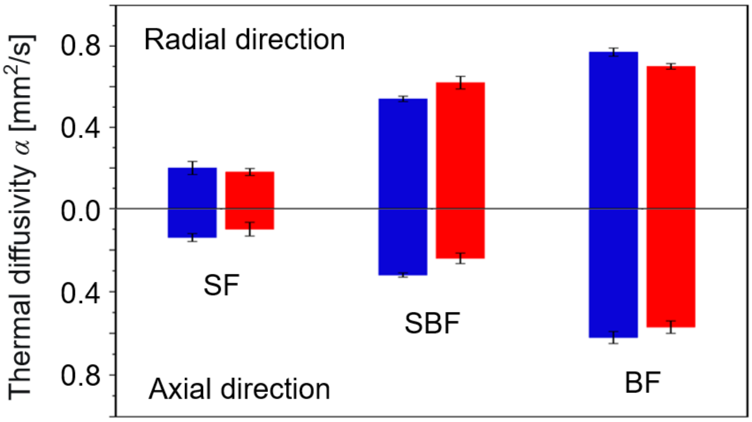

Figure R2. Radial and axial thermal diffusivity of SF, SBF, and BF samples.

The above discussion about the directional thermal diffusivity measurements was incorporated into the revised manuscript. **(Page 22, Lines 5-10; Page S25, Figure S11 of the SI)**

Comment #1.3: *The COMSOL simulations should include interfacial thermal resistance between the BN core and SiO_2 shell; if neglected, a justification must be provided.*

Response/Action: Thank you for raising this critical point regarding the interfacial thermal resistance (ITR) in our COMSOL simulations. In our current model, the ITR between the BN core and the SiO_2 shell was indeed not explicitly included. This simplification is based on a scale analysis of the Kapitza length.

Our justification is as follows: Reported thermal boundary resistances for h-BN/SiO_2, measured by time-domain thermoreflectance or similar techniques, are on the order of R_{int}~1.6×10^{-8}-3.4×10^{-8} $m^2 \cdot K \cdot W^{-1}$ [*Int. J. Heat Mass Transf.* 2023, 212, 124242; *Macromol. Rapid Commun.* 2025, 46, 2500078; *Ceram. Int.* 2024, 50, 9441–9452]. Given the relatively low intrinsic thermal conductivity of our mesoporous SiO_2 shell ($k\approx$ 0.1–1 $W \cdot m^{-1} \cdot K^{-1}$), the

resulting Kapitza length defined as $L_K=k\times R_{int}$ is only a few tens of nanometers. This length scale is over two orders of magnitude smaller than the characteristic dimensions of our core-shell fibers, where both the BN core radius and the SiO_2 shell thickness are on the micrometer scale. Consequently, the drop of interfacial temperature can be negligible compared to the temperature gradient across the shell, and the heat flow is dominated by the conduction effect through the porous SiO_2 matrix and by the radiative transfer at elevated temperature.

The above clarification for the interfacial thermal resistance was incorporated into the revised manuscript. **(Page 7, Lines 18-22; Page 8, Lines 1-2; Page S13, Lines 4-8 of the SI)**

Comment #1.4: *Incorporate laser irradiation experiments under pulsed mode to better simulate realistic HEL conditions and assess material response to repeated heating cycles.*

Response/Action: We thank the Reviewer for the insightful suggestion to evaluate our material under pulsed-mode laser irradiation and repeated thermal cycles, which better capture realistic high-energy laser (HEL) conditions. In response, we have conducted both pulsed laser tests and multiple thermal cycling experiments (120 second continuous-wave laser irradiation at 30 MW/m^2 followed by natural cooling).

As summarized in **Figure R3**, the SBF membrane exhibits outstanding resistance to pulsed laser irradiation. Under this more destructive mode, the BFs are penetrated at 1600 W, while the SFs withstand the irradiation up to 2500 W. Remarkably, our core-shell SBFs demonstrate only a ~2.4 mm deep damage crater at 3000 W and require 3600 W for full penetration, highlighting its superior defensive capability against intense attacks of pulsed laser.

As shown in **Figure R4**, the cyclic teats of laser irradiation further reveal the excellent durability and functional stability of SBF. Throughout the repeated thermal cycles, the SBF sample maintains its original high reflectivity, confirming the structural integrity of the silica shell (**Figure R4a**). The thermal transport properties evolve favorably: the radial thermal conductivity at 1000 °C increases gradually (**Figure R4b**), while the axial value remains stable, thereby enhancing the overall thermal anisotropy—a beneficial trait for in-plane heat spreading under repeated thermal loading. A degradation in compressive properties is observed after repeated laser cycling, arising from the accelerated exacerbation of structural rigidity defects

likely caused by boron nitride crystal degradation at high temperature (**Figure R4c**). Notably, despite the progressive increase in laser-induced damage depth with each irradiation cycle, the absolute depth remains comparatively low overall, confirming that the material retains excellent laser protection performance even after repeated exposure (**Figure R4d**).

Taken together, these additional experiments provide robust evidence that our SiO_2@BN core-shell membrane maintains critical functional properties—including high reflectivity, strong thermal anisotropy, and reliable laser protection—under pulsed and cyclic thermal conditions that closely mimic real-world HEL operation. These findings strongly support the practical potential of our material for advanced laser protection applications.

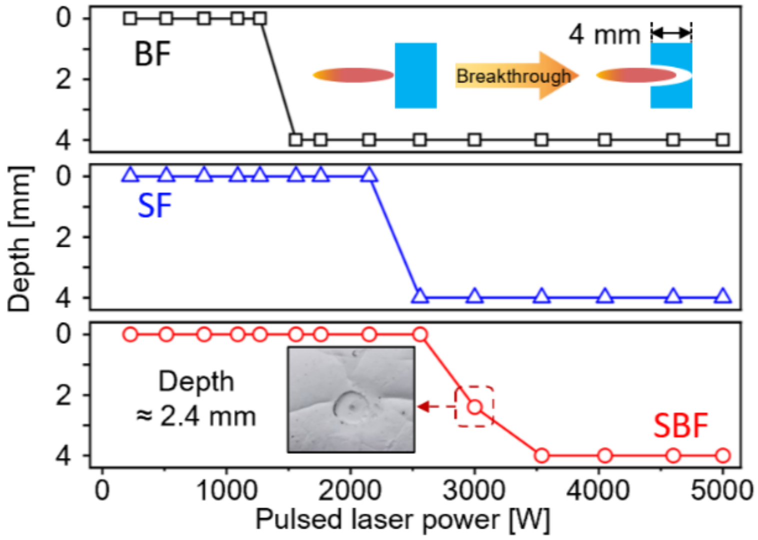

Figure R3. Damage assessment of BF, SF and SBF samples under the pulsed laser irradiation at varying power levels. The inset is a schematic illustration of the ablation depth induced by high-energy laser irradiation and the surface morphology of SBFs after irradiation.

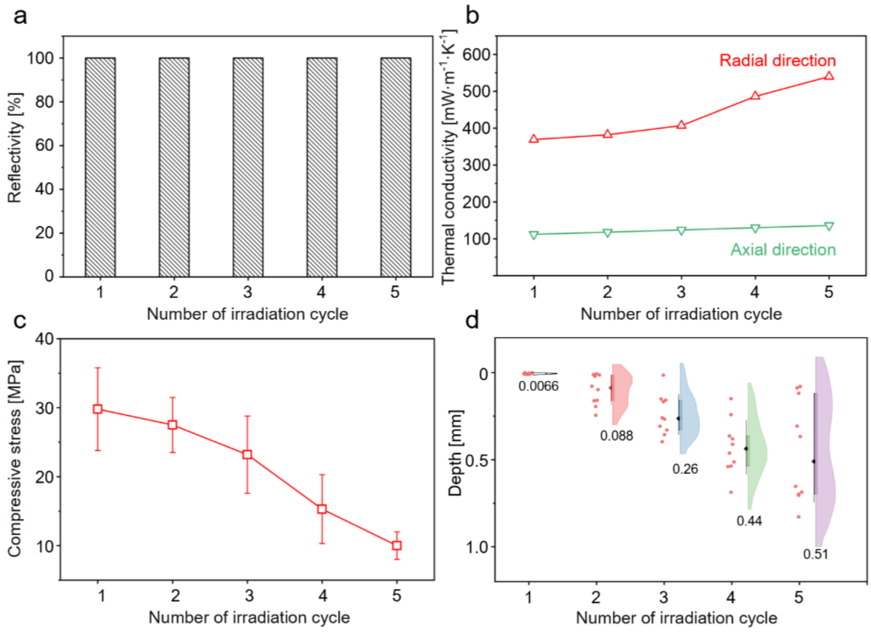

Figure R4. Characterization of the SBF sample after multiple cycles of high-energy laser irradiation: (a) reflectivity, (b) thermal conductivity along two principal directions, (c) compressive stress, and (d) depth of laser-induced damage.

The above results and discussion on pulsed-mode laser tests and cyclic irradiation performance were incorporated into the revised manuscript. **(Page 26, Lines 17-22; Page 27, Lines 1-4; Page 31, Lines 10-15; Page 32, Lines 1-11; Page 31, Figure 7 of the revised manuscript)**

Comment #1.5: *Extend thermal stability testing to high-humidity, oxidative, or thermocyclic environments to evaluate long-term durability.*

Response/Action: We appreciate the Reviewer's insightful recommendation regarding environmental reliability assessment. Accordingly, we have performed the accelerated aging tests under three demanding conditions: high-humidity aging (95% relative humidity, 35 days), oxidative aging (~99% O_2, 35 days), and thermal cycling (800 °C, 5 cycles).

As shown in Figure R5, the SBF membranes possess remarkable stability across all tested environments. The reflectivity of the SBF membrane remains unchanged at nearly 100% after prolonged exposure to high humidity, a high-oxygen atmosphere, and multiple thermal cycles (**Figure R5a**). This stability is attributed to the inherent chemical inertness of the silica

shell. The thermal conductivity of the SBF membrane at 50 °C shows no significant change after aging in humid or oxidative environments (**Figure R5b**). Conversely, a slight increase is observed following five thermal cycles at 800 °C, which is likely due to a phase transformation of the boron nitride crystals at high temperature. The compressive stress of the SBF membrane is unaffected by high humidity and oxidation (**Figure R5c**). However, a minor reduction is noted after the thermal cycling, possibly linked to alterations in the boron nitride crystal structure. Crucially, even after extended exposure to these demanding conditions, the SBF membrane remains intact and without damage subjected to high-energy laser irradiation at 30 MW/m² (**Figure R5d**). These results demonstrate the material's resilience and long-term functional reliability under demanding environments.

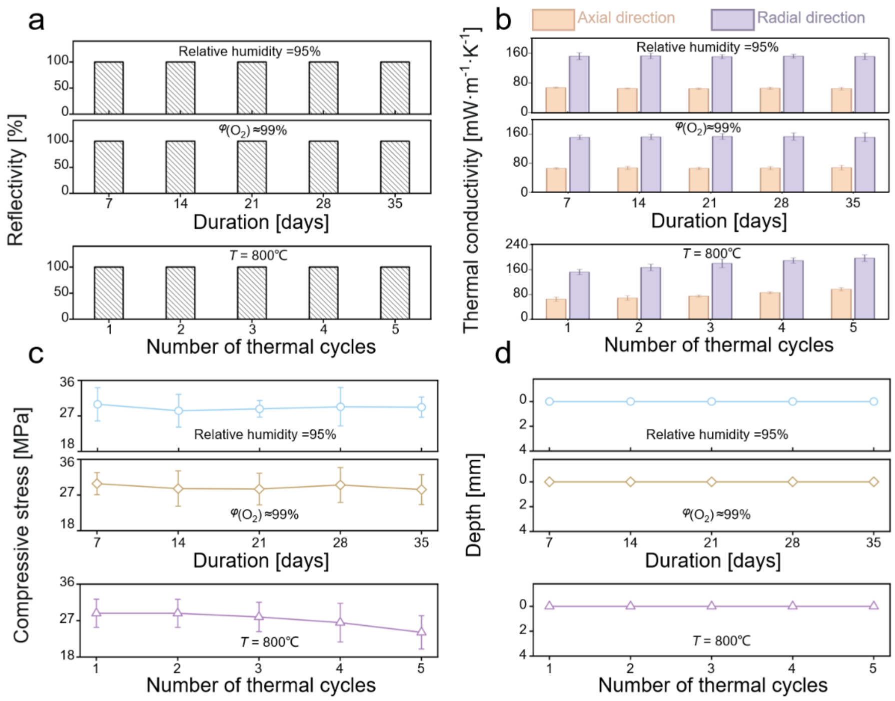

Figure R5. Evolution of SBF sample's properties under high-humidity, high-oxygen, and thermal cycling conditions: (a) reflectivity, (b) axial and radial thermal conductivity, (c) compressive stress, and (d) laser-induced damage depth.

The above discussion about environmental reliability assessment was incorporated into the revised manuscript. **(Page 32, Lines 12-22; Page 33, Lines 1-12; Page S30, Figure S16 of the SI)**

Comment #1.6: *Include dynamic simulations or empirical validation to confirm that Raman scattering contributes meaningfully to thermal dissipation as claimed.*

Response/Action: We thank the Reviewer for highlighting the importance of validating Raman scattering's contribution to thermal dissipation. Our experimental design provides empirical evidence through the comparison between Raman-inactive BF and Raman-active SF/SBF samples. As shown in Figure 4a, the lower front-surface temperature of SF and SBF under identical irradiation conditions demonstrates the enhanced thermal dissipation enabled by the SiO_2 shell's Raman activity. This temperature reduction corresponds to the Raman scattering mechanism that converts absorbed photon energy into lattice vibrations, thereby enhancing thermal radiation to the environment. The stronger Raman scattering in SF/SBF facilitates more efficient heat rejection compared to BF, distinct from the BN core's thermal conduction pathway shown in Figure 4d. The performance difference between these designed samples confirms that enhanced Raman scattering promotes better heat dissipation to the surroundings in our core-shell system.

The additional clarification regarding the relationship between enhanced Raman scattering and improved heat dissipation has been incorporated into the revised manuscript. **(Page 18, Lines 4-14; Page 20, Lines 9-12)**

Group B – Moderate Issues Requiring Minor Experiments, Discussion Expansion, or Formatting Adjustments

Comment #1.7: *Expand the literature review to cover recent advancements in anisotropic thermal materials and their applications in laser protection.*

Response/Action: We thank the Reviewer for this valuable suggestion to better contextualize our work within recent advancements. In response, we have significantly expanded the literature review in the *Introduction* section to include a discussion (**Page 4, Paragraph 2**) on the latest developments in anisotropic thermal management materials. This new paragraph specifically covers emerging material systems such as oriented polymer composites, and boron nitride-based scaffolds. We have incorporated 5 recent key references (References 26-30) that

critically examine both fundamental material advances and their practical implementation in thermal management systems. These additions provide a state-of-the-art perspective that properly frames the innovative aspects of our core-shell fiber approach within the current research landscape.

Comment #1.8: *Clarify the multi-order Raman scattering mechanism with more intuitive spectral decomposition or schematic support.*

Response/Action: We thank the Reviewer for suggesting improvements to the presentation of the multi-order Raman scattering mechanism. The revised Figure 3f now provides a clearer demonstration of this process by showing how the experimentally detected wavelengths in the scattering spectrum correspond to calculated Raman transitions. Specifically, the 932 nm wavelength observed in the first-order Raman scattering spectrum serves as the input for calculating the second-order Stokes and anti-Stokes shifts, yielding the 906 nm wavelength that is also experimentally verified. This progressive calculation-experiment correlation continues through the third and fourth order transitions, with all theoretically predicted wavelengths being consistently detected in the laser-irradiated scattering spectrum, thereby validating the multi-order Raman scattering process.

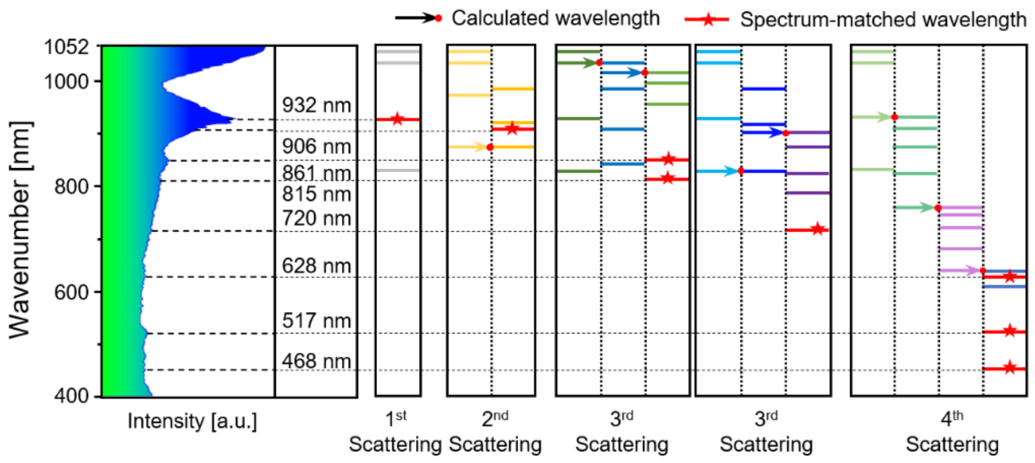

Figure R6. Spectral analysis and wavelength shift calculations for the SBF membrane under laser irradiation.

Comment #1.9: *Improve image quality for SEM/IR figures and ensure scale bars are clearly*

labeled and consistent.

Response/Action: We thank the Reviewer for this important suggestion. We have enhanced all SEM images and IR plots by re-acquiring micrographs under optimized conditions, standardizing scale bars with clear labeling, and optimizing contrast parameters. As a typical example, **Figure R7** shows the improved SEM micrographs of SF, SBF and BF membranes after high-energy laser irradiation, providing clearer morphological details with consistent dimensional reference across all panels. These enhancements have been applied throughout Figures 2, 5, and S3 in the revised manuscript.

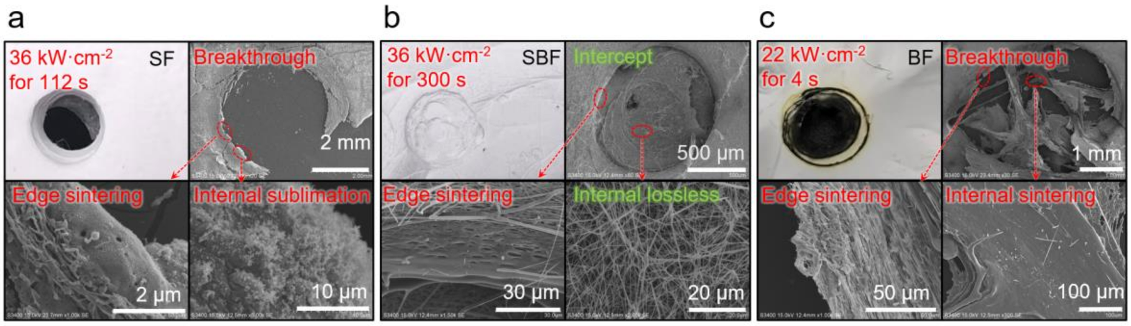

Figure R7. SEM micrographs showing microscopic damage morphology of (a) SF, (b) SBF, and (c) BF membranes after high-energy laser irradiation.

Comment #1.10: *Move detailed COMSOL parameters and repeatability data to the supplementary section to improve manuscript clarity.*

Response/Action: We thank the Reviewer for this constructive suggestion to improve manuscript clarity. In response, we have relocated the comprehensive COMSOL simulation parameters and detailed repeatability data to the Supplementary Information **(Section II. *Instrumentation and measurements* - *Heat transfer simulation*; Page S15, Figure S1 of the SI)**. The main text now retains only the essential simulation conditions and key results, accompanied by explicit cross-references to the supplementary materials.

Comment #1.11: *Provide more detailed instrumentation parameters for characterization techniques (e.g., XPS, SAXS, Raman).*

Response/Action: We thank the Reviewer for this valuable suggestion to improve the methodological rigor of our manuscript. In response, we have now expanded the *Methods*

section to include detailed instrumentation parameters and operational procedures for all characterization techniques. Specifically, we have added: (i) details on the X-ray spot size (400 μm) and the acquisition time for high-resolution scans (typically 5 minutes per region); (ii) details on the SAXS experimental setup, including the monochromator configuration, beam size, flux, and detector pixel size; and (iii) details on the laser power (10 mW), objective lens (100×, NA = 0.9), spectral resolution (2 cm^{-1}), grating type (600 grooves/mm), and the accumulation process for spectra (3 acquisitions). **(Page S6, Lines 6-11; Page S7, Lines 26-29; Page S8, Lines 1-4; Page S9, Lines 1-7)**

Comment #1.12: *Standardize citation formatting and correct inconsistencies in the reference list.*

Response/Action: We thank the Reviewer for pointing out this important detail. We have now thoroughly checked and standardized all citation formats throughout the manuscript.

Reviewer: #2

Comments: *This manuscript presents a SiO_2@BN core–shell nanofibrous membrane that combines ultrahigh optical reflectivity with anisotropic thermal management for high-energy laser protection.* ***The work is innovative in its material design, fabrication process, and multidimensional characterization.*** *In particular, the integration of BN crystalline cores with SiO_2 shells via multi-channel electrospinning and staged calcination is* ***technically impressive.*** *The experimental results convincingly demonstrate outstanding optical scattering, directional heat conduction, and mechanical strength, with extensive comparisons to existing materials.* ***Overall, the study is original, has significant potential for applications, and fits well within the scope of Nature Communications.*** *Nevertheless, there are several important issues that need to be addressed before the manuscript can be considered for publication.*

Response: We sincerely appreciate the Reviewer's positive assessment of our work as "*innovative in its material design*", "*technically impressive*" and having "*significant potential for applications*". However, the Reviewer has several concerns about the manuscript. Our

addresses to these concerns are as follows:

Comment #2.1: *The thermal conductivity and stability of BN are strongly affected by crystallinity and defects. While XRD and XPS are provided, the absence of quantitative Raman analysis or high-resolution TEM defect characterization leaves the microstructural basis for the reported performance insufficiently supported.*

Response/Action: We thank the Reviewer for raising this critical point regarding the need for microstructural evidence of thermal performance. To provide definitive experimental support, we have conducted high-resolution TEM (HRTEM) and selected-area electron diffraction (SAED) characterization. Cross-sectional HRTEM imaging of the SBF fiber clearly reveals its well-defined core-shell structure (**Figure R8a**). A magnified view of the core region (**Figure R8b**) shows regularly aligned lattice fringes, indicating the presence of a highly crystalline phase. Further magnification (**Figure R8c**) enables the measurement of an interplanar spacing of ~0.33 nm, which corresponds to the (0002) lattice plane of hexagonal boron nitride (hBN). The symmetric diffraction spots in the selected-area electron diffraction pattern (**Figure R8d**) further confirm the high crystallinity and structural integrity of the hBN core. These results provide direct evidence that the high crystalline quality and minimal defects in the BN cores underpin the outstanding thermal conductivity and stability reported in our work.

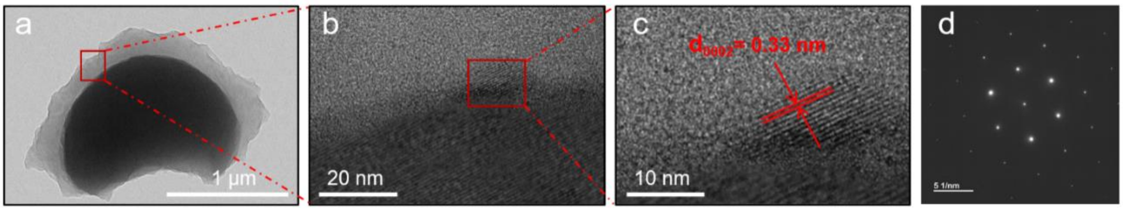

Figure R8. (a-c) High-resolution transmission electron microscopy images of the core-shell fiber at different magnifications and (d) corresponding selected-area electron diffraction pattern.

The above microstructural evidence from HRTEM and SAED characterization was incorporated into the revised manuscript. **(Page 12, Lines 17-22; Page 13, Lines 1-10; Page S19, Figure S5 of the SI)**

Comment #2.2: *The unique anisotropic thermal conduction is emphasized, but discussion of the interfacial thermal resistance between BN cores and SiO_2 shells is limited. Currently, only simulations and macroscopic experimental data are available, lacking direct characterization or a more in-depth theoretical explanation.*

Response/Action: We thank the Reviewer for raising this fundamental question regarding the interfacial thermal resistance (ITR) between the BN core and SiO_2 shell. To address the need for theoretical substantiation, we have conducted a detailed analysis based on the concept of Kapitza length.

Our calculations reveal that using reported thermal boundary resistance values for h-BN/SiO_2 interfaces (R_{int}~1.6×10^{-8}-3.4×10^{-8} $m^2\cdot K\cdot W^{-1}$) and the thermal conductivity of our mesoporous SiO_2 shell (k~0.1-1 $W\cdot m^{-1}\cdot K^{-1}$), the resulting Kapitza length ($L_K=k\times R_{int}$) is merely several to tens of nanometers — over two orders of magnitude smaller than the actual fiber dimensions. As a result, the interfacial temperature drop can be negligible compared to the temperature gradient across the SiO_2 shell, validating that heat transfer is dominated by the porous SiO_2 pathway. A more detailed discussion of this theoretical analysis can be found in our **Response of Comment #1.3**.

The above discussion about the theoretical justification of interfacial thermal resistance was incorporated into the revised manuscript. **(Page 7, Lines 18-22; Page S13, Lines 4-8 of the SI)**

Comment #2.3: *The COMSOL simulations only consider heat conduction, ignoring the inevitable radiation and convection effects under laser irradiation.*

Response/Action: We thank the Reviewer for this insightful comment regarding the heat transfer modes in our simulation. In the revised manuscript, we have improved our thermal modeling description to provide a more detailed physical representation, addressing both radiation and convection effects

Thermal Radiation: Our COMSOL model incorporates the surface-to-ambient radiation as a boundary condition, defined by the Stefan-Boltzmann law with a surface emissivity tied to

the temperature-dependent reflectivity of the material. Thus, the thermal radiation effect is actually considered in the COMSOL model.

Thermal Convection: Our analysis confirms that convective effects are negligible at the microscale of individual fibers. This conclusion is justified by the combined influence of the Knudsen effect and low Rayleigh number in our confined pore structures, which collectively suppress natural convection [*Processes*, 2023, 11, 2769; *Adv. Mater.*, 2025, 34, 202510796]. Therefore, neglecting the convection effect represents a valid simplification that allows us to focus on the fundamental conduction-radiation mechanisms within the core-shell structure. It is noteworthy that convective cooling at the membrane level remains crucial for overall performance, which will be addressed in our future work on system-level applications.

The above clarification regarding the heat transfer modes in our simulation was incorporated into the revised manuscript. **(Page 7, Lines 18-22; Page S13, Lines 10-15 of the SI)**

Comment #2.4: *The study focuses on single or short-duration laser exposures. Considering practical scenarios, cyclic laser shock tests or long-term thermal shock stability experiments are necessary to evaluate durability under realistic service conditions.*

Response/Action: We thank the Reviewer for emphasizing the importance of evaluating material durability under realistic service conditions. In response, we have conducted cyclic laser shock tests and long-term thermal stability evaluations. The membranes were subjected to multiple cycles of 120-second continuous-wave laser irradiation at 30 MW/m^2 followed by natural cooling to room temperature. The results demonstrate exceptional durability of our SBF membranes under these demanding conditions.

As detailed in our **Response to Comment #1.4**, the membranes maintained their structural integrity and functional performance throughout the testing. The SBF samples preserved near-perfect reflectivity after repeated thermal cycles, indicating the stability of the silica shell under thermal shocks. While the radial thermal conductivity showed a gradual enhancement with cycling, improving the thermal anisotropy, the compressive properties exhibited only minor degradation. Most importantly, the laser protection capability remained excellent, with only a

moderate increase in damage depth after multiple exposure cycles. These results validate the material's exceptional durability under realistic operating conditions of high-energy laser, confirming its ability to maintain protection performance through repeated thermal shocks.

The above discussion about the material durability under realistic service conditions was incorporated into the revised manuscript. **(Page 26, Lines 17-22; Page 27, Lines 1-4; Page 31, Lines 10-15; Page 32, Lines 1-11; Page 31, Figure 7 of the revised manuscript)**

Comment #2.5: *The conclusion that radial conduction is dominated by BN cores is not fully substantiated. Additional evidence is needed to demonstrate that heat preferentially flows through the BN core rather than the* SiO_2 *shell.*

Response/Action: We thank the Reviewer for raising the important point about radial conduction along the BN core. To address this concern, we have conducted additional experiments using fibers with varying core-shell ratios and performed thermal characterization.

Our investigation reveals three key findings that demonstrate the dominant role of BN cores in radial heat transfer: (1) The back-side temperature under laser irradiation decreases progressively with increasing BN core diameter (**Figure R9**), reaching a minimum at the optimal Si:B ratio of 0.6:0.4, where heat is most effectively guided radially through the BN network. As the BN core diminishes, the back temperature gradually increases due to weakened radial conduction. (2) The radial thermal conductivity shows significant enhancement with larger BN cores (**Figure R1b** in **Response of Comments #1.1**), while the axial conductivity remains unchanged. (3) The laser protection threshold demonstrates optimal performance at the 0.6:0.4 ratio under high-power conditions (**Figure R1c**). These evidences clearly demonstrate that the heat preferentially flows through the continuous BN core pathway rather than the SiO_2 shell, thereby validating our conclusion of the dominant role of BN cores in radial heat conduction.

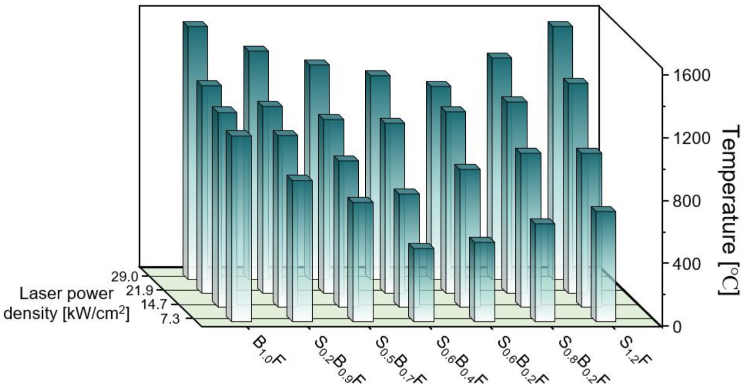

Figure R9. Back-side temperature of 2-mm-thick core-shell membranes under different laser power densities with various of outer SiO_2 (S) to inner BN (B) precursors.

The above experimental evidence demonstrating BN-core-dominated radial heat conduction was incorporated into the revised manuscript. **(Page 27, Lines 5-22; Page 28, Lines 1-8; Page S28, Figure S14 of the SI)**

Comment #2.6: *The analysis of tensile and compressive behavior is largely descriptive based on stress-strain curves. A more detailed mechanistic explanation of the cooperative role of core and shell during failure would strengthen the discussion.*

Response/Action: We thank the Reviewer for this valuable suggestion to strengthen the mechanistic understanding of the core-shell cooperation during mechanical failure. We have expanded the *Results and Discussion* section to provide a more detailed explanation of the failure mechanisms.

The added text reads: *Compression tests reveal that a 4 mm-thick SBF membrane exhibits exceptional compressibility, sustaining up to 95% strain with complete thickness recovery upon unloading (Figure 6a, inset). This behavior originates from the synergistic interaction between the core and shell components. In the initial linear elastic regime (up to ~40% strain), the porous amorphous SiO_2 shell acts as a compliant matrix that absorbs and distributes the compressive load [Ceram. Int. 2016, 42, 5504–5511]. Simultaneously, the continuous crystalline BN core functions as an internal scaffold, progressively bearing load during densification and providing the restoring force necessary for elastic recovery [Phys. Rev. B 2013,* ***87****, 045201]. This core–shell synergy yields exceptional tolerance to extreme strain*

*[Ceram. Int. 2024, **50**, 9441–9452; Adv. Funct. Mater. 2025, **35**, 2504706]. Unlike monolithic crystalline fibers, the SBF sustains ~95% compression without fracture, a behavior attributed to the rigid core which provides robust mechanical support and enables elastic recovery of the original morphology. The precise balance between silica's compliance and BN's rigidity is crucial: excessive BN content leads to brittle fracture, while insufficient BN compromises structural support (Figure 6c).*

*Under tensile loading, the SBF membrane demonstrates a distinctive two-stage failure process that enhances its damage tolerance (Figure 6d). The initial stress drop at ~20% strain corresponds to the sequential fracture of the rigid BN core filaments, while the subsequent stress recovery plateau (~20 MPa) results from effective load transfer to the continuous SiO_2 shell network [Compos. Part B Eng. 2017, **119**, 114–124]. This staged failure mechanism, analogous to fiber bridging in composite materials, allows the membrane to maintain structural integrity and dissipate energy progressively through shell deformation and fiber pull-out, ultimately achieving ~25% failure strain [J. Mater. Sci. Technol. 2026, **244**, 70–79]. The coordinated response of core and shell during tensile deformation significantly prolongs the load-bearing phase and improves the effective toughness compared to homogeneous ceramic materials.*

Comment #2.7: *It is recommended that the conclusion section more clearly highlight the advantages of this material over existing BN aerogels and ZrO_2-based materials in practical protection applications. The paper should also acknowledge the potential limitations of this process in terms of cost, scalable production, or complex environments.*

Response/Action: We appreciate the Reviewer's valuable suggestion to better contextualize our work's practical significance. We have revised the *Conclusion* section to more clearly articulate the specific advantages of our SiO_2@BN core-shell membranes over existing protection materials.

The revised text now explicitly states: "Compared to BN aerogels that suffer from poor mechanical stability and ZrO_2-based materials limited by their isotropic thermal conduction, our SiO_2@BN core-shell membranes offer a unique combination of near-perfect reflectivity

(>99.5%), exceptional thermal anisotropy (radial/axial conductivity ratio >15), and mechanical robustness (withstanding 95% compressive strain with full recovery) — addressing the critical challenge of simultaneously managing optical, thermal, and mechanical demands in practical HEL protection."

Furthermore, we have added a balanced discussion of current limitations: "While our electrospinning approach provides excellent control over fiber architecture, scaling to industrial production requires optimization of precursor costs and processing throughput. Additionally, performance validation in complex multi-physics environments combining thermal, mechanical, and humidity stresses represents an important direction for future development."

The above discussion about the advantages and limitations of our SiO_2@BN core-shell membranes was incorporated into the revised manuscript. **(Page 33, Line 22; Page 34, Lines 1-10)**

Comment #2.8: *In Figure 5b, several benchmark materials are drawn from older reports. More recent advances in nanoporous ceramics and metal-based composite coatings should be incorporated to provide a fairer and more convincing comparison.*

Response/Action: We thank the Reviewer for this constructive suggestion. We have updated Figure 5b (also **Figure R10**) to include recent advancements in both nanoporous ceramics and metal-based composite coatings, providing a more current and comprehensive comparison. Specifically, we have added performance data from recent high-performance materials including: (1) a 3D carbon fiber-reinforced ZrB_2-SiC composite with a porous ceramic matrix [*Corros. Sci.* 2024, 236, 112263]; (2) a $RE_2Ti_2O_7$ (RE = Y, La) ceramic coating with high reflectivity and low thermal conductivity [*J. Am. Ceram. Soc.* 2025, 108, e20599]; (3) an Al-based composite coating reinforced with Al_3BC particles [*Appl. Surf. Sci.* 2025, 697, 163045]. It is demonstrated that our SiO_2@BN core-shell membranes maintain competitive advantages in both reflectivity and thermal management capabilities.

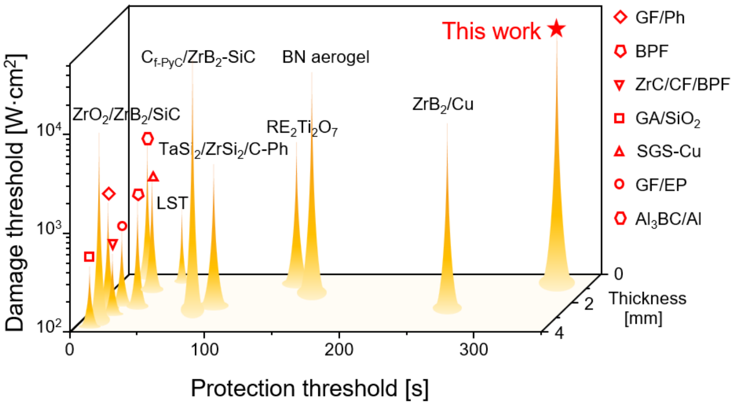

Figure R10. Comparative analysis of laser protection thresholds between our study and previously reported materials at different membrane thicknesses.

The above discussion about the comparison with other materials was incorporated into the revised manuscript. **(Page 24, Lines 5-8; Page 23, Figure 5b of the revised manuscript)**

Comment #2.9: *The Supplementary Information contains essential experimental details, yet these are not sufficiently cross-referenced in the main text. Clearer integration (e.g., "see Supplementary Fig. S3 for FTIR evolution") would improve readability and transparency.*

Response/Action: We thank the Reviewer for this constructive suggestion to improve the integration between the main text and Supplementary Information. We have revised the manuscript to include explicit cross-references to all supplementary figures, tables, and methods.

Reviewer: #3

Comments: *The author developed a three-layer co-spun electrospinning method and successfully prepared core-shell structured nanofibers.* ***The structural design of SiO2-BN nanofibers is novel, which provides a new idea for the design of thermal protection materials.***

Response: We sincerely appreciate the Reviewer's positive assessment of our work as " *The structural design of SiO2-BN nanofibers is novel, which provides a new idea for the design of thermal protection materials*". However, the Reviewer has several concerns about the

manuscript. Our addresses to these concerns are as follows:

Comment #3.1: ***(i)*** *From tri-layer core-shell structure to SiO_2@BN core-shell nanofibers, what is the diameter contraction ratio of the fiber?* ***(ii)*** *How to control the discharge of gas during PVP decomposition in the core is very critical. Especially in the process of B_2O_3 to BN, how to control NH_3 to enter the interior through the surface pore without reacting with the surface?*

Response/Action: We thank the Reviewer for raising these important questions regarding **(i)** fiber diameter contraction and **(ii)** gas transport management during thermal processing. Our addresses to those concerns are as follows:

(i) Regarding the diameter contraction, the fiber shrinkage can be directly quantified from the SEM images of the fibers at different stages, which are provided in the original manuscript (e.g., Figure 2b-e). Based on these images, we calculated the diameter contraction ratio to be ~15–20% during the calcination process. This controlled shrinkage, resulting from organic removal and shell densification, has been explicitly stated in the *Results and Discussion* section of revised manuscript. **(Page 12, Lines 11-13)**

(ii) Concerning the issue of gas transport management, we have expanded the discussion in the *Methods* section to provide a more detailed explanation of our thermal schedule. The key is the carefully designed calcination profile, which employs controlled, low ramp rates and strategic dwell stages. This protocol ensures the complete and gradual decomposition of PVP and other organics, allowing the gaseous products to diffuse out through the mesoporous shell before it undergoes significant densification and pore closure. Subsequently, during the B_2O_3-to-BN nitridation step, the same mesoporous network remains accessible and allows NH_3 to diffuse inward to the core. The SiO_2 shell is chemically inert to NH_3 under our processing conditions, which prevents the parasitic surface reaction. Furthermore, by controlling the NH_3 flow rate and partial pressure, we promote this through-thickness diffusion to the core. The absence of BN signatures on the outer fiber surfaces in our existing EDS mappings (**Figure R11**) supports the success of this core-localized nitridation strategy.

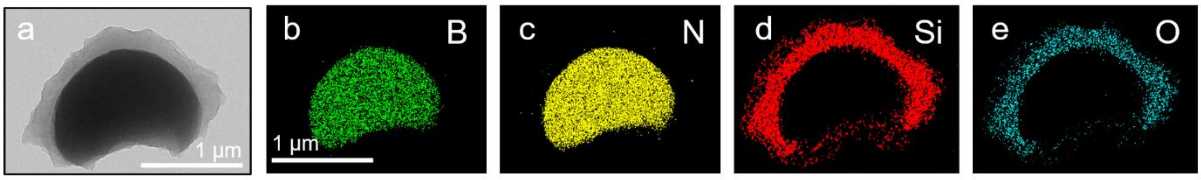

Figure R11. EDS elemental maps of the core－shell nanofiber showing the spatial distribution of B, N, Si, and O.

The above clarifications regarding the processing parameters were incorporated into the revised manuscript. **(Page 11, Lines 6-8; Page 12, Lines 17-22; Page 13, Lines 1-10; Page S19, Figure S5 of the revised manuscript)**

Comment #3.2: *In anisotropic SiO_2@BN core-shell nanofibrous membrane, how to ensure that the distribution of BN in a single fiber is continuous? If there is an interruption, it will produce a thermal resistance effect.*

Response/Action: We thank the Reviewer for raising this critical question regarding the continuity of BN core distribution within individual fibers. We have addressed this fundamental issue through both processing optimization and systematic characterization.

To maximize the continuity of BN cores, we precisely controlled the core-shell ratio through tri-axial electrospinning parameters, particularly the feed rates of core and shell precursors. Meantime, the rheological matching between phases and stable core jet maintenance are essential for forming continuous BN pathways prior to calcination.

Given the difficulty in directly visualizing the continuity along centimeter-scale fibers through microscopic techniques, we employed an indirect but robust approach by examining the correlation between core-shell ratio and thermal transport properties. Three key indicators of performance are used to demonstrate continuous pathways of BN cores: the radial thermal conductivity shows monotonic enhancement with increasing BN core diameter (**Figure R1b** in **Response of Comments #1.1**), the laser damage threshold reaches maximum at the optimal core-shell ratio (**Figure R1c**), and the thermal insulation performance exhibits corresponding improvement (**Figure R9** in **Response of Comments #2.5**). These coordinated enhancements across multiple thermal and optical metrics indicate continuous BN pathways rather than

interrupted segments, because discontinuous cores would produce inconsistent or degraded performance trends due to additional interfacial resistances.

The above discussion regarding the continuity of BN core distribution was incorporated into the revised manuscript. **(Page 27, Lines 5-22; Page 28, Lines 1-8; Page S28, Figure S14 of the SI)**

Comment #3.3: *The fiber membrane is composed of a combination of single fibers interwoven longitudinally and transversely. The laser action and the surface will produce strong diffuse reflection and fiber surface reflection, which exists in both SF and SBF fiber membranes. This is related to the surface state of the fiber membrane and the fiber overlapping structure, and seems to have no direct relationship with BN.*

Response/Action: We thank the Reviewer for this insightful comment regarding the origin of reflection in our fiber membranes. We fully agree that the strong diffuse and surface reflection primarily arises from the membrane's surface state and interwoven fiber topology—a characteristic shared by both the SF and SBF membranes.

This observation also highlights the innovative aspect of our core-shell design. The SiO_2 shell and fibrous architecture governs the optical reflection, while the strategically incorporated BN cores provide complementary thermal management functionality. This deliberate functional separation enables both components to achieve their respective optimal performance—the SiO_2 shell maintaining near-perfect reflectivity while the BN core facilitating efficient anisotropic heat dissipation. The resulting synergistic effect produces a material system that simultaneously excels in both optical and thermal management capabilities, ultimately enabling the exceptional laser protection performance demonstrated in our work.

The above discussion about the core-shell architecture was incorporated into the revised manuscript. **(Page 18, Lines 4-14)**

Comment #3.4: ***(i)*** *In Figure6b, does the length of the fiber change under compression? SiO_2@BN is an inorganic fiber and has a large brittleness.* ***(ii)*** *Why does a single fiber deform greatly?* ***(iii)*** *In Figure6f, how to obtain mechanical and thermal performance results at 1500℃*

and 1800°C?

Response/Action: We thank the Reviewer for these insightful questions regarding **(i)** fiber length changes during compression, **(ii)** mechanism behind macroscopic deformation, and **(iii)** methodology for high-temperature measurements. Our addresses to those concerns are as follows:

(i) Fiber length changes during compression: The apparent macroscopic strain does not arise from elastic stretching of individual inorganic fibers. Our closer examination indicates that the compression is primarily accommodated through pore-spacing reduction and local rearrangement at fiber junctions, while individual fibers remain essentially inextensible and brittle.

(ii) Mechanism behind macroscopic deformation: The observed macroscopic deformation primarily results from network-level structural rearrangement rather than substantial elastic deformation of individual SiO_2@BN fibers. As illustrated in **Figure R12**, compression is achieved through pore closure and slippage at fiber crossovers, with minimal change in individual fiber length. This deformation mechanism enables the core-shell structure to sustain ~95% compression without fracture, attributed to the rigid BN core providing mechanical support and enabling recovery through synergistic core-shell interaction.

(iii) Methodology for high-temperature measurements: The mechanical results at 1500°C were obtained using a high-temperature universal testing machine (CMT4204, Shenzhen Xinsansi Material Testing Co., Ltd., China). The thermal insulation performance data up to 1800°C represent front-back temperature differences derived from IR thermal imaging under laser irradiation conditions.

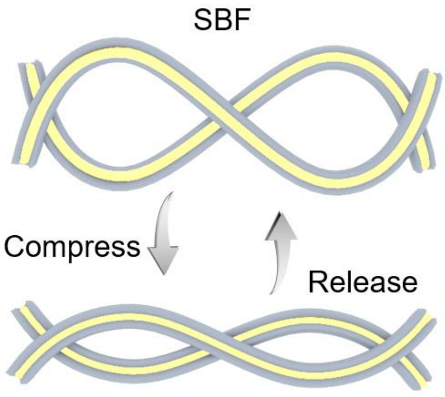

Figure R12. Schematic illustration of morphological change of core–shell structured fibers

under compression.

The above discussion about the material's mechanical behavior was incorporated into the revised manuscript. **(Page 29, Lines 15-21; Page 28, Figure 6b of the revised manuscript)**

Comment #3.5: ***(i)*** *Please explain why SBF has a higher ability to withstand high power laser than SF and BF?* ***(ii)*** *Why does SBF not show breakdown phenomenon when surface melting occurs?*

Response/Action: We thank the Reviewer for these insightful questions regarding **(i)** the origin of superior laser protection capability of SBF and **(ii)** the mechanism preventing catastrophic breakdown under surface melting conditions. Our addresses to those concerns are as follows:

(i) Superior laser protection capability of SBF: This enhanced capability stems from the synergistic combination of three complementary mechanisms within our core-shell architecture. First, the porous SiO_2 shell provides exceptional optical shielding through strong broadband reflection, effectively minimizing energy absorption. Secondly, the continuous BN core enables rapid in-plane heat spreading, efficiently dissipating residual energy and preventing localized hot-spot formation. Thirdly, the hierarchical fiber network offers mechanical buffering capacity, accommodating thermal strain while maintaining structural integrity. This multi-level protection strategy enables SBF to outperform both SF (lacking efficient heat spreading) and BF (lacking effective optical shielding).

(ii) Mechanism preventing catastrophic breakdown under surface melting conditions: This remarkable behavior results from the functional redundancy in our core-shell design. When surface melting occurs in the SiO_2 shell under extreme thermal load, the BN core remains structurally intact and continues to provide efficient thermal transport. The softened SiO_2 layer actually serves as a self-sealing barrier that smoothens surface irregularities while preserving the continuous thermal pathways maintained by the underlying BN network. This design prevents thermal runaway and maintains both structural integrity and thermal management capability under surface melting conditions, which would cause conventional materials to fail catastrophically.

The above discussion about the protection and failure-resistant mechanisms was incorporated into the revised manuscript. **(Page 25, Lines 20-22; Page 26, Lines 1-7)**

Reviewer: #4

Comments: *The manuscript reports the preparation of a SiO_2@BN core–shell nanofibrous membrane by ingenious tri-layer electrospinning and controlled calcination, followed by achieving ultrahigh optical reflectivity and superior thermal insulation for high-energy laser protection. This work represents a significant advance in high-temperature thermal protection especially for flexible ceramic continuous nanofibers.* ***The study is intriguing and is expected to be valuable for applications that extend well beyond high-energy laser protection.*** *It is clearly articulated and logically structured, and the characterization techniques employed are state-of-the-art. I therefore* ***anticipate broad reader interest and recommend publication on Nature Communications****, provided the following issues are addressed.*

Response: We sincerely appreciate the Reviewer's positive assessment of our work as " *The study is intriguing and is expected to be valuable for applications that extend well beyond high-energy laser protection*" and having "*broad reader interest and recommend publication on Nature Communications*". However, the Reviewer has several concerns about the manuscript. Our addresses to these concerns are as follows:

Comment #4.1: *The recent work by Pan et al. (Science Bulletin, 2025, 70, 722-732) on BN/SiO_2 core–shell nanofiber membranes has not been cited or discussed. The novelty of the present SiO_2@BN architecture relative to the previously reported BN/SiO_2 arrangement must be explicitly clarified, preferably in the introduction, so that the incremental advance is unambiguously established.*

Response/Action: We thank the Reviewer for bringing this relevant work to our attention. We have cited and discussed the study by Pan et al. in the *Introduction* section of our revised manuscript.

The BN/SiO_2 core–shell nanofiber membrane reported by Pan et al. represents a significant

advance in fibrous ceramic materials for thermal management. Their work successfully integrated a thermally insulating SiO_2 shell with a radiative cooling BN core within a single nanofiber architecture using coaxial electrospinning and controlled calcination. This structural design achieved a dual-functional thermal management, combining ultra-low thermal conductivity with high reflectivity and emissivity. The material also demonstrated excellent flame retardancy, maintaining structural integrity after direct flame exposure, along with good flexibility and chemical stability in various corrosive environments. This demonstration of multifunctional integration established an important architectural paradigm for advanced thermal protection materials.

Inspired by this concept, our study explores a specifically optimized SiO_2@BN configuration for ultrahigh-energy laser protection. We employ tri-axial electrospinning to ensure BN core continuity for enhanced anisotropic heat dissipation, while utilizing the SiO_2 shell as a continuous reflective barrier. This represents a focused architectural refinement and application-driven specialization of the core–shell principle established by Pan et al.

The above discussion about the contribution of Pan et al. has been incorporated into the revised manuscript. **(Page 5, Lines 6-10)**

Comment #4.2: *The core–shell morphology remains insufficiently substantiated. Figure S2 mapping result shows no nitrogen, while the boron also distributed in the shell instead. Additional high-resolution line scans or phase maps are therefore required to confirm that the core is indeed BN and that the shell is* SiO_2.

Response/Action: We thank the Reviewer for raising this critical point regarding the need for more definitive evidence of the core-shell morphology. To address this concern, we have conducted high-resolution characterization using HRTEM, SAED, and EDS to confirm the BN-core/SiO_2-shell architecture (**Figure R13**).

Cross-sectional HRTEM imaging of the SBF clearly reveals its well-defined core-shell structure (**Figure R13a**). A magnified view of the core region (**Figure R13b**) shows regularly aligned lattice fringes, indicating the presence of a highly crystalline phase. Further magnification (**Figure R13c**) enables the measurement of an interplanar spacing of

approximately 0.33 nm, which corresponds to the (0002) lattice plane of hexagonal boron nitride (hBN). The symmetric diffraction spots in the selected-area electron diffraction (**Figure R13d**) pattern further confirm the high crystallinity and structural integrity of the hBN core. EDS elemental mapping on the fiber cross-section clearly reveals the core-shell architecture. The core region is predominantly composed of boron (B) and nitrogen (N), confirming the presence of a BN-rich phase, while the shell exhibits strong signals for silicon (Si) and oxygen (O), consistent with SiO_2. Notably, no detectable B or N signals are observed within the shell region, indicating a sharp compositional transition and the high purity of the SiO_2 shell. **These results provide direct evidence of the well-defined BN-core/SiO_2-shell structure.**

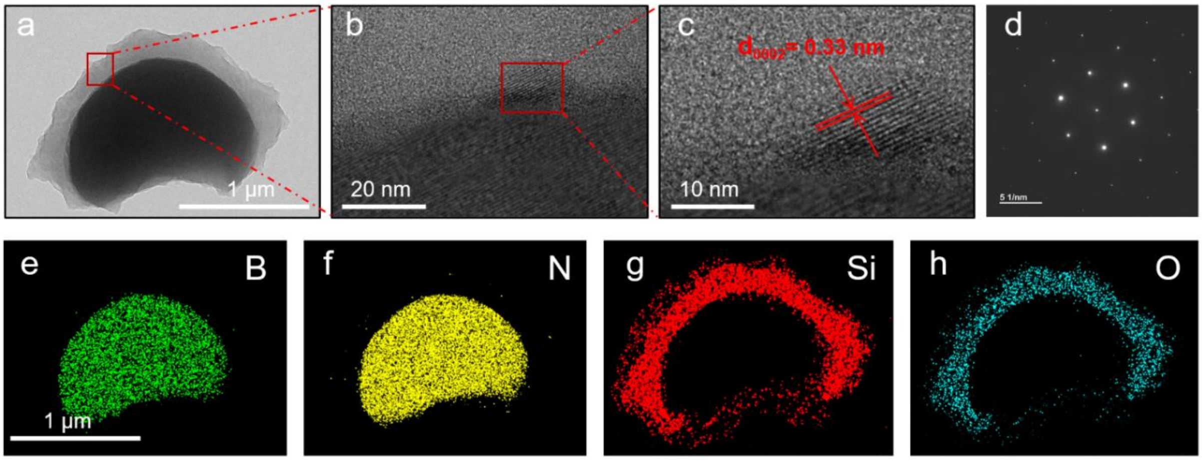

Figure R13. (a-c) High-resolution transmission electron microscopy images of the core-shell fiber at different magnifications. (d) Corresponding selected-area electron diffraction pattern. (e-h) EDS elemental maps of the core-shell nanofiber showing the spatial distribution of B, N, Si, and O.

The above results of the core-shell morphology were incorporated into the revised manuscript. **(Page 12, Lines 17-22; Page 13, Lines 1-10; Page S19, Figure S5 of the SI)**

Comment #4.3: *The assertion that CTAB generates a nanoporous shell is currently based on TEM images alone; nitrogen absorption isotherms should be acquired to quantify porosity and to validate the templating mechanism.*

Response/Action: We thank the Reviewer for highlighting the need for quantitative porosity

characterization. To address this concern, we have performed N_2 physisorption measurements to quantitatively analyze the pore structure evolution.

Figure R14 shows the evolution of pore structure throughout the multi-stage calcination process of SBF membranes. The adsorption-desorption isotherms and corresponding BJH pore-size distribution curves clearly show the development of well-defined mesopores (1-3 nm) following CTAB removal, with a high specific surface area of approximately 400 m²/g. Most significantly, these mesopores disappear after the final densification step, demonstrating the successful closure of the templated porosity and confirming CTAB's role as an effective pore-forming agent. **The quantitative data establish a clear correlation between the template removal and mesopore formation, with subsequent densification eliminating the porosity as designed.**

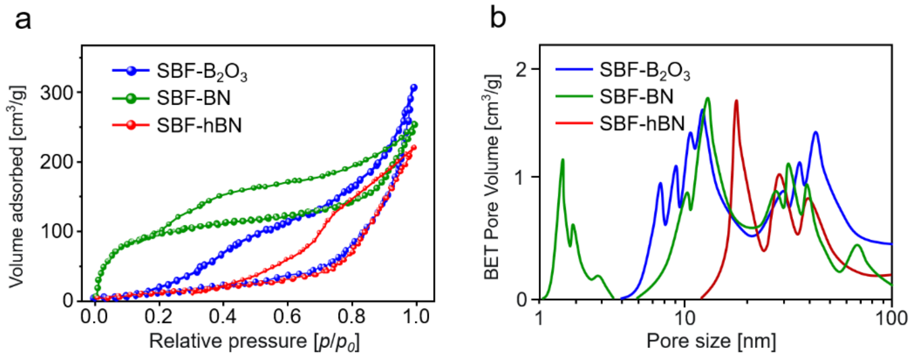

Figure R14. (a) N_2 adsorption-desorption isotherms and (b) corresponding pore-size distribution curves of core-shell fibrous membranes at three key stages of fabrication process.

The above results about the porosity characterization were incorporated into the revised manuscript. **(Page 12, Lines 5-9; Page S4, Figure S4 of the SI)**

Comment #4.4: *The axial/radial terminology appears to have been reversed. The abstract states that heat is "rapidly dissipated radially while insulated axially"; however, for a fiber geometry the converse is expected. This inconsistency should be rectified throughout the text.*

Response/Action: We thank the reviewer for this insightful observation. The terms "radial" and "axial" in our manuscript are defined with respect to the macroscopic membrane geometry, not individual fibers. We have clarified this distinction in the abstract and throughout the text

to prevent any potential misunderstanding.

Comment #4.5: *The thermal simulation was run for 300 s, whereas the actual laser irradiation experiment lasted only 30 s. The rationale for this discrepancy must be provided to avoid a questionable model.*

Response/Action: We thank the Reviewer for their careful attention to this detail. We would like to clarify that the experimental laser irradiation tests were indeed conducted for 300 s, as stated in our *Results and Discussion* section and supported by the supplementary video for the 36 kW·cm^{-2} irradiation condition. The simulation time of 300 s was therefore selected to be consistent with the experimental duration, allowing for a direct comparison between the modeled and experimental thermal response as the system approaches a near-steady state. We have further clarified this alignment in the *Methods* section of the revised manuscript. **(Page 36, Lines 1-2)**

Comment #4.6: *The possibility of elemental boron or boron-rich non-crystalline phases forming within the shell has not been excluded. More results are required to verify the exclusive presence of h-BN and to assess the thermal stability contributions of any amorphous boron in the shell.*

Response/Action: We thank the Reviewer for raising this important concern regarding potential boron-rich phases in the shell region. To address this issue, we have conducted high-spatial-resolution cross-sectional HRTEM-EDS analysis and complementary thermal property characterization.

Our high-spatial-resolution cross-sectional HRTEM-EDS analysis (**Figure R13**) provides definitive evidence that boron (B) and nitrogen (N) signals are exclusively co-localized within the core region, with no detectable B signal in the SiO_2 shell area. This clear spatial separation strongly indicates the absence of elemental boron or boron-rich phases in the shell. Additional characterization details are provided in our **Response to Comment #2.2**. Furthermore, the measured thermal transport properties provide additional validation ─ the high radial thermal conductivity observed in our samples would be significantly compromised by the presence of

amorphous boron in the shell, which would introduce strong phonon scattering centers and degrade heat transport performance.

The above discussion about potential boron-rich phases was incorporated into the revised manuscript. **(Page 7, Lines 18-22; Page 8, Lines 1-2; Page S13, Lines 4-8 of the SI; Page 12, Lines 197-22; Page 13, Lines 1-10; Page S19, Figure S5 of the SI)**

Comment #4.7: *The compression micrographs schematically in Figure 6b suggest a marked diameter reduction, which is atypical for ceramic fibers in my opinion.*

Response/Action: We thank the Reviewer for raising this important point regarding the compression behavior of the ceramic fibers. We have conducted further analysis to clarify the underlying mechanism of the apparent diameter reduction observed during compression.

Our investigation reveals that the apparent macroscopic strain is primarily accommodated through structural reorganization at the network level rather than through actual diameter reduction of individual ceramic fibers. As demonstrated in **Figure R12** of the **Response to Comment #3.4**, the compression process involves pore closure and rearrangement at fiber junctions, while the individual SiO_2@BN fibers maintain their structural integrity without significant diameter change. **This network-level deformation mechanism, combining pore collapse and fiber reorientation, enables the membrane to achieve exceptional compressibility (up to 95% strain) while preserving the intrinsic mechanical properties of the ceramic components.**

The above explanation about the deformation behavior was incorporated into the revised manuscript. **(Page 29, Lines 15-21; Page 28, Figure 6b of the revised manuscript)**

Comment #4.8: *This manuscript would benefit from a brief demonstration of how the SiO_2@BN core–shell ratio can be tuned and how such tunability affects laser damage thresholds and thermal insulation performance.*

Response/Action: We thank the Reviewer for suggesting a demonstration of how the SiO_2@BN core-shell ratio can be tuned and how such tunability affects laser damage thresholds and thermal insulation performance. To address this comment, we have conducted additional

experiments to investigate the relationship between structural parameters and functional performance.

The core-shell ratio can be controlled by adjusting the feed rates of precursor solutions during tri-axial electrospinning, enabling the variation of BN core diameter and SiO_2 shell thickness. Our results reveal distinct optimization trends across multiple performance metrics. As shown in **Figure R1b** of **Response to Comments #1.1**, the radial thermal conductivity exhibits significant enhancement with increasing BN core diameter, confirming the crucial role of continuous BN pathways in thermal management. **Figure R1c** demonstrates that the laser damage threshold reaches maximum protection capability at the optimal Si:B ratio of 0.6:0.4 under high-power conditions. Meanwhile, **Figure R9** of **Response to Comments #2.5** shows that thermal insulation performance, characterized by back-side temperature measurements, exhibits optimal values at this same ratio where temperature rise is minimized due to efficient radial heat spreading. **These results demonstrate the fact that the change of core-shell ratio enables the fine tuning of both laser protection and thermal insulation performance.**

The above discussion about the effect of core-shell ratio was incorporated into the revised manuscript. **(Page 27, Lines 5-22; Page 28, Lines 1-8; Page S28, Figure S14 of the SI)**

(Over)

Responses to the Reviewers' comments

We thank the Reviewers for their careful review of our manuscript and for providing constructive comments and suggestions to improve the quality of the manuscript. Our point-by-point responses to these comments are given below. The corresponding contents were incorporated into the manuscript and highlighted in blue.

Reviewer: #1

Comments: *The authors have substantially revised their manuscript entitled "Anisotropic SiO_2@BN Core–Shell Nanofibrous Membrane with Ultrahigh Optical Reflectivity and Superior Thermal Insulation for High-Energy Laser Protection." All major concerns raised in the first review—regarding structural parameter optimization, thermal-diffusivity measurements, interfacial thermal resistance justification, pulsed- and cyclic-laser tests, environmental durability, and Raman-related thermal dissipation—have been addressed through additional experiments and clarifications. The revised manuscript now presents a comprehensive and coherent dataset that convincingly supports the claimed multifunctional performance of the SiO_2@BN core–shell system. Figures, image quality, and citation consistency have also been notably improved.*

Assessment of Revisions

The newly added results, including laser-flash thermal diffusivity measurements, parametric studies of core–shell ratios, and cyclic irradiation tests, provide direct and quantitative support for the anisotropic thermal management concept.

The justification for neglecting interfacial thermal resistance through Kapitza-length estimation is physically reasonable, and the extended environmental-stability tests further validate material reliability. The overall narrative—from structural design to optical scattering, anisotropic heat conduction, mechanical integrity, and laser-shielding performance—is now logically continuous and experimentally substantiated.

The manuscript is significantly strengthened and, in my view, meets the scientific and technical standards of Nature Communications.

Response: We sincerely thank the Reviewer for their thoughtful and constructive assessment of our revised manuscript, and for their positive overall conclusion that "*The manuscript is significantly strengthened and, in my view, meets the scientific and technical standards of Nature Communications*". We also gratefully acknowledge the Reviewer's remaining comments and suggestions, which have provided us with an opportunity to further refine the presentation and discussion of our work. Below, we address each point in detail, and corresponding edits have been incorporated into the revised manuscript.

Comment #1.1: *The discussion of the Raman scattering contribution remains qualitative. A simple energy-balance estimate or reference-based evaluation of photon-to-phonon conversion efficiency would make this mechanism more convincing.*

Response/Action: We thank the Reviewer for the suggestion to quantify the contribution of Raman scattering. To provide a more quantitative basis, we have performed an estimation based on the well-established Raman scattering cross-section of silica (SiO_2), which is on the order of 10^{-30} $cm^2 \cdot sr^{-1} \cdot molecule^{-1}$ as confirmed by experimental measurements in applied spectroscopy [*J. Appl. Phys.* 1997; 82 (7): 3499–3507].

In our work, the high surface area and mesoporous network of the SiO_2 shell provide a large effective scattering volume. Based on the relative intensities of the pump (1064 nm) and the first-order Stokes (~932 nm) peaks in our recorded spectra (Figure 3e, f) and by applying the standard relationship for Raman scattering intensity to the aforementioned cross-section and our experimental parameters, we estimate that approximately 1–5% of the incident laser energy is channeled into optical phonons of the SiO_2 network via the multi-order Raman scattering process. Although this fraction is modest in absolute terms, this inelastic, non-linear pathway actively contributes to converting incident photon energy into lattice vibrations (heat), thereby supporting the observed lateral heat diffusion.

This quantitative discussion has been added to the revised manuscript. **(Page 18, Lines 10-20)**

Comment #1.2: *Please add a brief comment on whether the "radial-conduction/axial-*

insulation" principle could be extended to other oxide/nitride systems (e.g., SiO_2@AlN, ZrO_2@BN).

Response/Action: We thank the Reviewer for this insightful suggestion. In the revised manuscript, we have added an assessment in the Conclusion section regarding the generalizability of this principle to other oxide/nitride systems.

The added text reads: *The specific applicability of this principle to new compound pairs can be inferred based on the difference in thermal conductivity between the selected oxide and nitride, as well as the chemical compatibility between the materials.*

Comment #1.3: *Some newly added figures (R4–R5 and related SI data) could be more clearly linked in the main text by transitional sentences specifying which property each test validates.*

Response/Action: We have added transitional sentences in the main text to explicitly link the newly added figures and data to the specific material properties they validate. **(Page 31, Lines 11-13; Page 32, Lines 15-17)**

Comment #1.4: *Consider including one sentence quantifying achievable membrane area or electrospinning throughput, to contextualize potential for large-scale fabrication.*

Response/Action: We have incorporated a discussion on scalability into the Conclusion section, which explicitly acknowledges the potential for scaling up the fabrication process based on the presented laboratory-scale electrospinning method.

The added text reads: *The laboratory-scale electrospinning approach possesses inherent scalability for larger-area production, as throughput can be significantly enhanced through established technical optimizations such as multi-needle arrays.*

Reviewer: #4

Comments: ***The revised manuscript has addressed most of my concerns and demonstrates further improvement in quality.***

Response: We sincerely appreciate the Reviewer's positive assessment of the revised

manuscript as "*The revised manuscript has addressed most of my concerns and demonstrates further improvement in quality*". However, the Reviewer has several concerns about the manuscript. Our addresses to these concerns are as follows:

Comment #4.1: **(i)** *That said, while the authors clarified in their response that "the terms 'radial' and 'axial' in our manuscript are defined with respect to the macroscopic membrane geometry, not individual fibers," Figure 1c still appears to use the orientation of a single fiber as reference. Moreover, since the key innovation of this work is fundamentally based on individual fiber design, I strongly recommend that the take full attentions to this point throughout the manuscript. There is no inherent contradiction or complexity between thermal conduction at the single-fiber level and that of the fibrous membrane as a whole. However, I believe the authors have not adequately addressed this relationship.* **(ii)** *This is particularly evident in the abstract, where they state: "By virtue of dual-channel electrospinning and precise calcination, the membrane structure achieves ultrahigh reflectivity (~100% at 1064 nm) while enabling anisotropic thermal conduction—rapidly dissipating heat radially across the membrane plane while insulating axially through its thickness." Such a description deviates from conventional fiber science and is likely to cause significant confusion among readers especially in fiber area.* **(iii)** *Alternatively, the membrane should at least be clearly labeled with 'radial' and 'axial'in the main text figure to clearly distinguish the difference.*

Response/Action: We thank the Reviewer for emphasizing the critical need for clarity in defining the "radial" and "axial" directions and for the opportunity to better articulate the relationship between single-fiber design and macroscopic membrane properties.

(i) We fully agree with the Reviewer that there is no inherent contradiction between the two scales, and the macroscopic anisotropy stems directly from the fiber-level architecture. In the revised manuscript, we have added a clearer explanation in the *Design rationale* section. The added text clarifies that within each layer of the membrane, the individual core-shell fibers are predominantly oriented in-plane. Consequently, the phonon transport along the high-conductivity BN core primarily occurs along the fiber axis, which collectively establishes efficient in-plane (radial) heat dissipation across the membrane. Conversely, the heat transfers

through the membrane thickness (axial direction) is impeded by the intrinsic low conductivity of the SiO_2 shell and, more significantly, by the inter-layer pores and interfaces arising from the stacked fibrous network. This combination of aligned intra-fiber conduction and inter-fiber/ inter-layer resistance is the fundamental origin of the membrane's pronounced thermal anisotropy.

(ii) We sincerely appreciate the Reviewer's strict critique on this point. The description in the original abstract may be potentially confusing. We have revised the relevant sentence to accurately reflect the conduction mechanism at the membrane level: "*By virtue of dual-channel electrospinning and precise calcination, the structure achieves ultrahigh reflectivity (~100% at 1064 nm) while enabling anisotropic thermal conduction—rapidly dissipating heat in the membrane plane while insulating through its thickness.*"

(iii) As suggested, we have explicitly labeled the "radial" (in-plane) and "axial" (through-thickness) directions directly on the membrane schematic in **Figure 1c**. Furthermore, a definitive sentence has been added in the main text caption or the corresponding description to formally establish this frame of reference for all subsequent discussions (i.e., *Throughout this work, 'radial' and 'axial' refer to directions within the macroscopic membrane plane and thickness, respectively*).

These revisions ensure precise, consistent, and conventional terminology is used throughout the manuscript, accurately linking the innovative fiber design to its macroscopic functional outcome. **(Page 2, Lines 8-11; Page 7, Lines 12-14; Page 8, Lines 4-10)**

Comment #4.2: *It is unclear whether the EDS analysis (Figure S5) was performed after the "preliminary calcination" or the final "high-temperature calcination" step. This distinction is critical for interpreting the results: If it is after the preliminary calcination, one might expect to observe a boron gradient or a diffuse interface due to the transitional state of the core-shell formation. The presence of a sharp interface, as shown, would therefore be unexpected and require explanation. If it is after the high-temperature calcination, a significant discrepancy arises between the core-shell ratio evident in the EDS mapping (Figure S5) and that shown in the TEM image of Figure 2e. This inconsistency in the observed core-to-shell dimensions needs*

to be clarified.

Response/Action: We thank the Reviewer for this meticulous observation, which allows us to clarify a technical point. The EDS analysis in Figure S5 was conducted on fibers **after the final high-temperature calcination**.

The apparent difference in the core-to-shell dimensions between Figure 2e and Figure S5 stems from the fact that these images feature fibers from two distinct samples, which were prepared with intentionally different precursor feed ratios (S:B) to serve specific analytical purposes. Specifically, Figure 2e displays a representative fiber from the performance-optimized structure (S:B = 0.6:0.4) central to the main findings of this study. In contrast, the fiber analyzed in Figure S5 was selected from a batch synthesized with a larger BN core (S:B = 0.2:0.9). This specific sample was chosen to ensure that a clear and unambiguous Selected-Area Electron Diffraction (SAED) pattern could be obtained directly from the core region, thereby confirming its crystallinity.

This variation in the core-to-shell ratio is an inherent aspect of the fabrication process. We have investigated its effect on material performance, as detailed in Supplementary Figure S14.

Comment #4.3: *Besides, a noticeable inconsistency exists in the reported fiber diameters between the SEM and TEM images. The SEM images in Figures 2f and 2g suggest fiber diameters on the order of several hundred nanometers. In contrast, the TEM images in Figures 2c and 2e, based on their scale bars, indicate diameters in the micrometer range. The authors should verify the accuracy of all scale bars and provide a coherent explanation for this apparent discrepancy.*

Response/Action: We thank the Reviewer for their meticulous observation regarding the discrepancy in fiber diameters between the SEM and TEM images. Upon careful verification, we confirm that this discrepancy originates from the inaccuracies in the scale bar annotations in the original figures.

We have corrected all scale bars in the relevant figures (**Figures 2c, 2e, 2f, 2g** and also shown in **Figure R1**) to ensure they accurately reflect the fiber diameters. The corrected images now consistently show that the fiber diameters are on the order of several hundred nanometers

across all microscopy techniques.

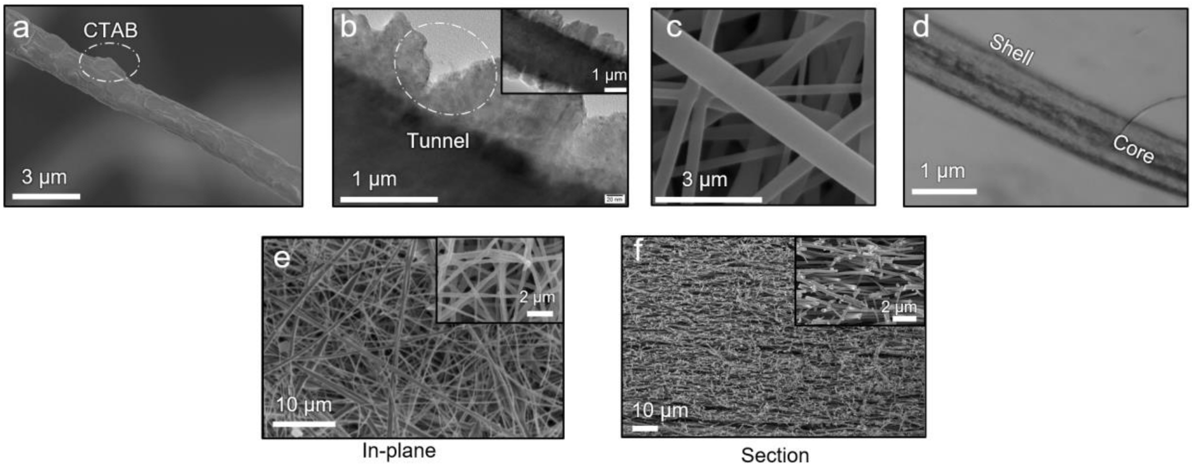

Figure R1. **a**, SEM image of the rough fiber surface prior to calcination. **b**, TEM image showing surface channels after preliminary calcination. **c**, SEM image of fiber surface following high-temperature calcination. **d**, TEM image of cross-sectioned fibers after high-temperature calcination. **e**, **f**, SEM images illustrating the in-plane and cross-sectional structures of the SBF membrane, respectively.

Comment #4.4: *I believe the authors have adequately addressed Reviewer 3's concerns.*

Response: Thank you for your positive feedback and for acknowledging our efforts in addressing the concerns raised by Reviewer #3.

(Over)

Responses to the Reviewers' comments

We sincerely thank you for your time and positive evaluations. We are thrilled that the revised manuscript has met your expectations. Below is our point-by-point response.

Reviewer: #1

Comments: *The revisions to the manuscript fully address my concerns and meet my expectations. I am pleased to recommend its acceptance in the current form.*

Response: We deeply appreciate your thorough evaluation and positive recommendation. Thank you for your constructive feedback throughout the review process.

Reviewer: #4

Comments: *My concerns have been addressed in the revisions.*

Response: We sincerely thank you for your time and for confirming that all your concerns have been successfully resolved.

(Over)
